# Rewiring phospholipid biosynthesis reveals resilience to membrane perturbations and uncovers regulators of lipid homeostasis

**DOI:** 10.15252/embj.2021109998

**Published:** 2022-02-21

**Authors:** Arun T John Peter, Sabine N S van Schie, Ngaam J Cheung, Agnès H Michel, Matthias Peter, Benoît Kornmann

**Affiliations:** ^1^ Institute of Biochemistry ETH Zürich Zürich Switzerland; ^2^ Department of Biochemistry University of Oxford Oxford UK; ^3^ Present address: Department of Biology University of Fribourg Fribourg Switzerland

**Keywords:** budding yeast, lipid homeostasis, lipid transport, organelles, transposon mutagenesis, Membranes & Trafficking, Metabolism, Organelles

## Abstract

The organelles of eukaryotic cells differ in their membrane lipid composition. This heterogeneity is achieved by the localization of lipid synthesizing and modifying enzymes to specific compartments, as well as by intracellular lipid transport that utilizes vesicular and non‐vesicular routes to ferry lipids from their place of synthesis to their destination. For instance, the major and essential phospholipids, phosphatidylethanolamine (PE) and phosphatidylcholine (PC), can be produced by multiple pathways and, in the case of PE, also at multiple locations. However, the molecular components that underlie lipid homeostasis as well as the routes allowing their distribution remain unclear. Here, we present an approach in which we simplify and rewire yeast phospholipid synthesis by redirecting PE and PC synthesis reactions to distinct subcellular locations using chimeric enzymes fused to specific organelle targeting motifs. In rewired conditions, viability is expected to depend on homeostatic adaptation to the ensuing lipostatic perturbations and on efficient interorganelle lipid transport. We therefore performed genetic screens to identify factors involved in both of these processes. Among the candidates identified, we find genes linked to transcriptional regulation of lipid homeostasis, lipid metabolism, and transport. In particular, we identify a requirement for Csf1—an uncharacterized protein harboring a Chorein‐N lipid transport motif—for survival under certain rewired conditions as well as lipidomic adaptation to cold, implicating Csf1 in interorganelle lipid transport and homeostatic adaptation.

## Graphical abstract

Systematic rerouting of membrane phospholipid biosynthesis uncovers organelle‐specific mechanisms of fatty acid metabolism.

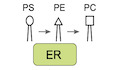

## Introduction

Establishing the characteristic membrane lipid composition of organelles is thought to be critical for their proper function. This is achieved by targeting lipid biosynthesis and modifying enzymes to specific compartments, intracellular transport of lipid molecules across these compartments, and homeostatic responses to perturbations in the environment. Surprisingly, a large fraction of the molecular components and pathways in this fundamental cellular process are still elusive. For instance, it is largely unknown which route(s) a given lipid utilizes to navigate the multiple membranes of eukaryotic cells. Lipid transport is carried out by vesicular traffic as well as by dedicated lipid transport proteins (LTPs). LTPs catalyze the transfer of lipid molecules between two membranes by shielding them from the aqueous environment. LTPs often function at regions of close apposition (10–30 nm) between organelles—termed membrane contact sites (MCSs)—found between various compartments, including membranes connected by vesicular trafficking pathways (Gatta & Levine, 2017; Reinisch & Prinz, 2021). Indeed, phospholipids and sterols are transported between the ER and the plasma membrane even after inhibition of the secretory pathway, indicating that non‐vesicular trafficking pathways contribute to lipid transfer at these sites (Vance *et al*, 1991; Schnabl *et al*, 2005; Quon *et al*, 2018).

Moreover, there is redundancy in lipid exchange routes even within non‐vesicular trafficking pathways, as exemplified with mitochondria. In the yeast *Saccharomyces cerevisiae*, two pathways have been implicated in lipid transport at mitochondria: the ER‐Mitochondria Encounter Structure (ERMES) pathway and the Mcp1‐Vps13 pathway (Kornmann *et al*, 2009; Lang *et al*, 2015; Park *et al*, 2016; John Peter *et al*, 2017; preprint: John Peter *et al*, 2021). Structural analysis revealed hydrophobic cavities, both in the SMP domains of multiple ERMES components and in Vps13, which facilitates lipid exchange *in vitro* (Jeong *et al*, 2016, 2017; AhYoung *et al*, 2017; Kumar *et al*, 2018; Li *et al*, 2020). The fact that mitochondria are also in contact with multiple organelles including the PM, peroxisomes, and lipid droplets (Elbaz‐Alon *et al*, 2014; Hönscher *et al*, 2014; Schuldiner & Bohnert, 2017; Shai *et al*, 2018) makes it plausible that these organelles could also function as potential donor and/or acceptor organelles for lipid exchange. These redundancies in lipid exchange likely exist for other organelles as well.

**Figure 1:**
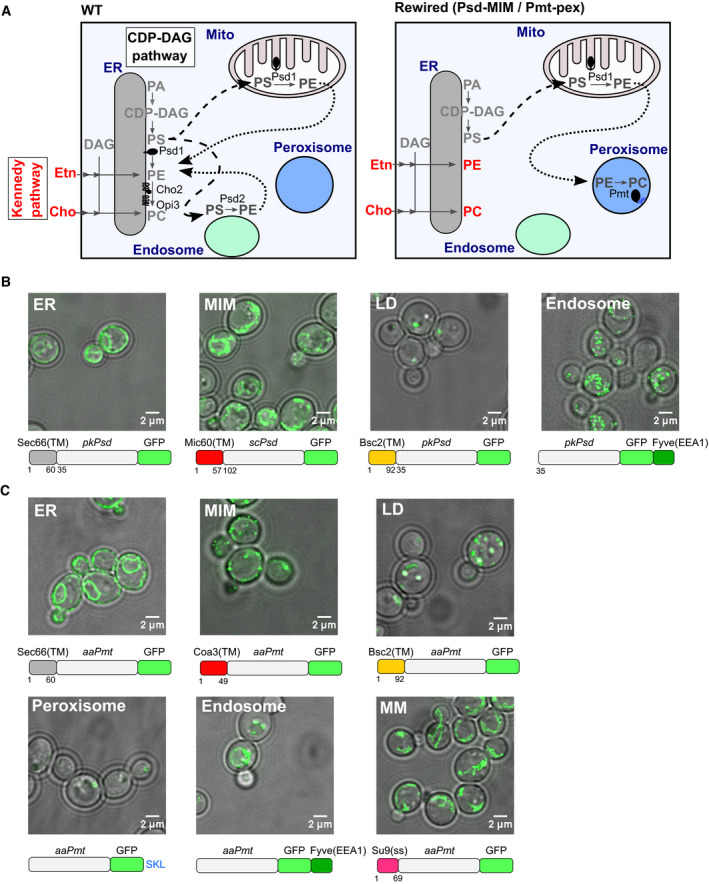
Rewiring PE and PC synthesis to expose lipid trafficking routes Scheme depicting the topology of the CDP‐DAG and Kennedy (red) pathways producing PE and PC in wild‐type (WT) yeast cells (left panel), and an example of a rewired condition in which PE synthesis is directed to the mitochondrial inner membrane (MIM) and PC synthesis to peroxisomes (pex) (right panel). Black arrows indicate PS (dashed) and PE (dotted) lipid transport that must occur to enable the sequential enzymatic reactions.Localization of the GFP‐tagged PE‐synthesizing enzyme (Psd) targeted to the indicated organelles in *choppΔ* cells, grown in SD medium supplemented with 10 mM choline. Images shown are either a single Z‐slice (ER construct) or a maximum intensity projection of several Z‐sections (other constructs); LD, lipid droplets; MM, mitochondrial matrix. A scheme depicting each chimeric Psd construct is shown under the corresponding microscopy image.Localization of the GFP‐tagged PC‐synthesizing enzyme (Pmt) targeted to the indicated organelles in *choppΔ* cells, grown in SD medium supplemented with 10 mM ethanolamine. Images shown are either a single Z‐slice (ER construct) or a maximum intensity projection of several Z‐sections (other constructs). A schematic depicting each chimeric Pmt construct is shown under the corresponding microscopy image.

An additional layer of redundancy is observed at the level of lipid synthesis; two pathways, namely the Kennedy and the cytidyl‐diphosphate‐diacylglycerol (CDP‐DAG) pathways (Fig 1A, left panel), produce phosphatidylethanolamine (PE) and phosphatidylcholine (PC). The Kennedy pathway utilizes ethanolamine and choline—primarily obtained from the medium—to convert diacylglycerol (DAG) directly to PE and PC, respectively (Fig 1A, left panel) (Kennedy & Weiss, 1956). By contrast, the CDP‐DAG pathway uses CDP‐DAG generated from phosphatidic acid (PA) and a series of enzymatic reactions to produce phosphatidylserine (PS), PE and PC in sequential order (Carman & Henry, 1999). In yeast, the dually localized PS decarboxylase Psd1 produces PE in the mitochondrial inner membrane (MIM) and ER, while Psd2 synthesizes PE at endosomes (Gulshan *et al*, 2010; Friedman *et al*, 2018). The production of PC by trimethylation of PE is performed by the ER‐localized enzymes, Cho2 and Opi3. Either pathway alone is necessary and sufficient for optimal growth, indicating that lipids are distributed throughout the cell from different locations.

Importantly, cells are likely to adapt to the absence of one of these pathways by activating homeostatic responses. Indeed, mechanisms are known for sensing lipid levels and lipid packing/saturation (Young *et al*, 2010; Covino *et al*, 2016), which are coupled to transcriptional, post‐transcriptional, or allosteric responses. A spectacular example is the adaptation to the lack of PC synthesis which involves remodeling of fatty acids in phospholipids (Bao *et al*, 2021).

Genetically uncovering lipid transport routes and homeostatic adaptive mechanisms requires removal of the multiple layers of redundancy. One way to achieve this goal is to delete endogenous enzymes and redirect synthesis of specific phospholipids to defined organelles through the expression of chimeric enzymes, as was successfully used to demonstrate PE transport from peroxisomes (Raychaudhuri & Prinz, 2008) or PS transport from non‐native organelles (Shiino *et al*, 2021). Importantly, in this strategy, data interpretation depends on the targeting accuracy of the chimeric enzymes to the intended organelles.

Here, we extended this rewiring approach (i.e., minimalizing and rerouting phospholipid synthesis) by confining PE and PC synthesis to distinct combinations of organelles. We then performed genetic screens to identify factors, the perturbation of which results in a fitness gain or loss in rewired conditions. We focus on *CSF1*, encoding a protein with a Chorein‐N lipid transport motif (Levine, 2019; Lees & Reinisch, 2020), and uncover its role in homeostatic adaptation of the lipidome.

## Results

### Rewired yeast phospholipid synthesis as a strategy to remove layers of redundancies

We postulated that by constructing yeast strains with simplified and rewired phospholipid synthesis pathways (Fig 1A, right panel), we could (i) remove layers of redundancy and (ii) expose homeostatic adaptations and trafficking routes between organelles of interest. To do this, we first generated a *cho2Δ opi3Δ psd1Δ psd2Δ* strain (hereafter referred to as *choppΔ*) that is incapable of producing PE and PC via the CDP‐DAG pathway and therefore relies on supplementation with ethanolamine and choline for growth. To rewire lipid synthesis, we engineered lipid synthesizing enzymes to make PE and PC in distinct pairs of (or in the same) organelle, by fusing them to specific targeting sequences (Fig 1). Specifically, we utilized a PS decarboxylase (Psd) lacking the transmembrane domain, either from yeast (*sc*Psd) or *Plasmodium knowlesi* (*pk*Psd) and a soluble PE methyltransferase from *Acetobacter aceti* (*aa*Pmt, hereafter referred to as Pmt) for the production of PE and PC, respectively (Hanada *et al*, 2001; Choi *et al*, 2012; Kobayashi *et al*, 2014). We directed the Psd and Pmt enzymes to either the ER, the mitochondrial inner membrane (MIM), endosomes (endo), or lipid droplets (LD), with the Pmt enzyme targeted in addition to the peroxisomal lumen (pex) or the mitochondrial matrix (MM).

We expressed the chimeric enzymes on a plasmid under the control of the TEF promoter in the *choppΔ* strain, except for *scPSD*, which was expressed from its own promoter (Friedman *et al*, 2018). Targeting of the enzymes was verified by microscopy (Fig 1B and C, Appendix Figs S1–S13). Indeed, the ER‐ and mitochondria‐targeted enzymes revealed fluorescence patterns characteristic of these organelles (Fig 1B and C, Appendix Figs S1A, S4, S5, S10 and S13). Likewise, Pmt targeted to the MIM stained the mitochondrial outline (Appendix Fig S1B and S9), but also accumulated as bright puncta, suggesting aggregation. This did not however preclude enzyme activity (see below). Both lipid droplet (LD)‐ and peroxisome lumen (pex)‐targeted enzymes co‐localized with a LD (Erg6‐mCherry) and a peroxisome marker (mCherry‐SKL), respectively (Appendix Fig S2). We targeted the endosome using a PI3P‐binding FYVE domain, in order to avoid transit of the proteins through the early secretory pathway, and potential ectopic activity. Both endosome‐targeted Psd and Pmt (Psd‐FYVE and Pmt‐FYVE) assembled in perivacuolar puncta that were positive for the endocytic tracker FM4‐64, consistent with late endosome localization. In addition, the Pmt‐FYVE construct localized to vacuoles themselves (Fig 1C, Appendix Fig S7). Thus, both engineered enzymes were successfully re‐targeted to endocytic compartments.

Since an altered lipid composition in the rewired strains could affect organelle integrity and interfere with protein targeting, we validated the localization of all enzyme constructs in the various combinations of PE and PC synthesis (Appendix Fig S4–S13) in the *choppΔ* background. As both Psd and Pmt enzymes were GFP‐tagged, we mutated the GFP of one of the enzymes to a “dark” state to verify proper targeting of the other enzyme. We did not observe gross differences in enzyme targeting in any rewired condition.

It is important to note however that we cannot exclude that some enzymatic activity is borne by a small fraction of mislocalized protein (with the exception of mitochondria matrix‐directed Pmt, as discussed below). Indeed, a minor fraction of LD‐ and peroxisome‐targeted Pmt was detected in the ER in some cells (Appendix Fig S6 and S8). Similarly, because most constructs respect the native topology of the enzymes with the active site facing the cytosol, it is possible that some of the activity might happen *in trans* on other membranes at sites of interorganelle contact.

### Rewired yeast strains are viable and produce all phospholipids

**Figure 2:**
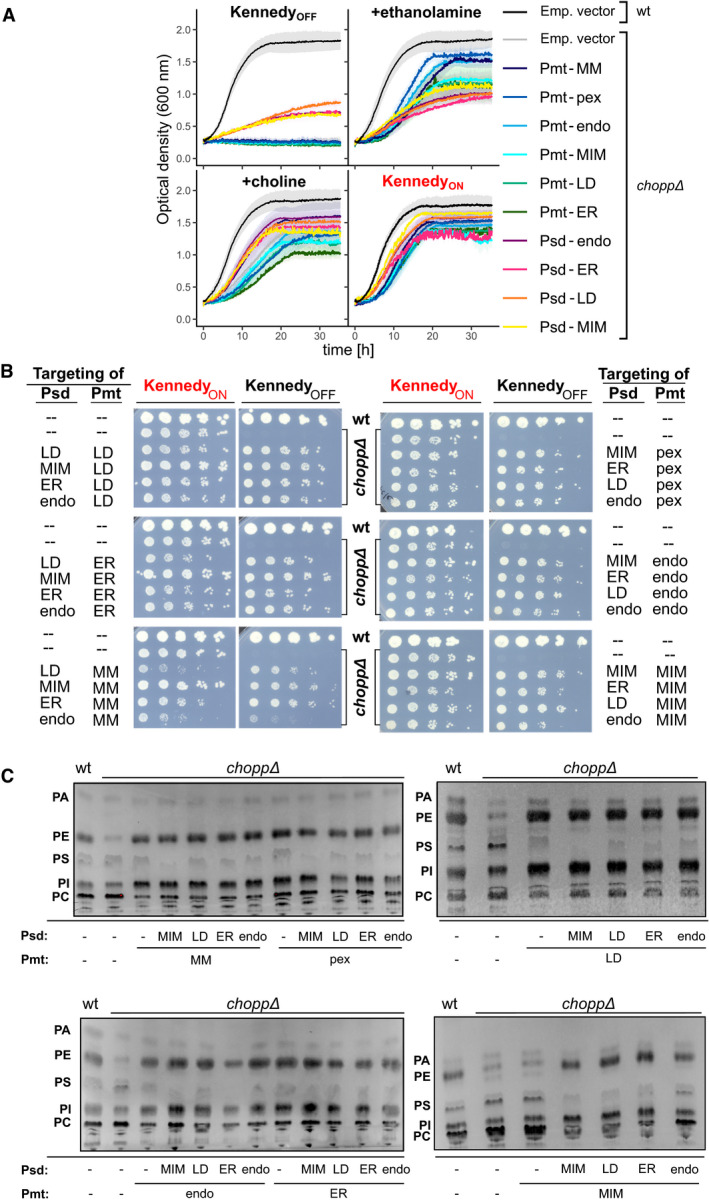
Rewired yeast cells are viable and produce all phospholipids Cells of the indicated genotypes were pre‐cultured in medium with 10mM ethanolamine and 10mM choline and subsequently in medium lacking them for 5 h, before being diluted in the indicated medium for growth curve analysis. Emp. vec. refers to cells bearing an empty vector. Data shown are the mean ±SEM of three independent replicates, except for Psd(MIM) (two experiments).Five‐fold serial dilutions of strains of the indicated genotypes (wt cells and *chopp*Δ cells harboring empty vectors or *chopp*Δ cells expressing chimeric Psd and Pmt enzymes). Cells were grown on SD‐HIS‐LEU (‐HL) medium (Kennedy_OFF_) or SD‐HL containing ethanolamine and choline (Kennedy_ON_).Thin‐layer chromatography (TLC) analysis of steady‐state phospholipid profiles of cells of the indicated genotypes. Where Psd and/or Pmt are missing (‐), *chopp*Δ cells were grown in the presence of ethanolamine and/or choline. Bands corresponding to the phospholipids PA, PS, PE, PI, and PC are indicated.

To examine whether the rewired CDP‐DAG pathway supports growth when the Kennedy pathway is inactive, we performed growth assays either in the presence or absence of exogenous ethanolamine and choline (conditions hereafter termed Kennedy_ON_ and Kennedy_OFF_, respectively). As expected, the *choppΔ* strain could not grow in the Kennedy_OFF_ conditions (Fig 2A and B). Single supplementation with ethanolamine or choline alone allowed growth of the *choppΔ* strain at various rates. Indeed, it is known that *psd1 psd2* double mutants can grow in the sole presence of choline. This is due to the fact that some PE can be synthesized from the breakdown product of long‐chain bases (Birner *et al*, 2001). This pathway is sufficient to generate a pool of PE but unable to supply enough precursor to PC biosynthesis via the CDP‐DAG pathway, explaining the auxotrophy of these strains for either ethanolamine or choline. It has also been observed that *cho2 opi3* double mutants can grow to a certain degree in the absence of choline (Summers *et al*, 1988). The basis for this residual growth is unclear but might be related to the recent finding that PC is not absolutely essential for cell function (Bao *et al*, 2021) or to impurities in growth media (Renne *et al*, 2020). In line with this, expression of any *PSD* alone led to a growth restoration equivalent to ethanolamine supplementation. However, cells expressing either Psd or Pmt targeted to any organelle grew better when supplied with both ethanolamine and choline (Fig 2A), showing the importance of both PE and PC for optimal cell growth.

We engineered a total of 24 combinations in which Psd and Pmt were targeted to various organelles. Remarkably, growth was restored in all rewired strains in the absence of ethanolamine and choline, albeit to varying extents, indicating that the chimeric enzymes bypass the need for the Kennedy pathway (Fig 2B). Growth was most robust when Psd was localized to the mitochondrial inner membrane (MIM), which is normally the major site of PE synthesis (Rosenberger *et al*, 2009). Cell growth was especially limited when Pmt was targeted to the mitochondrial matrix (MM). This was not likely due to the inability of matrix‐targeted Pmt to meet PC synthesis demand, since in some rewired conditions, cells grew even slower in the Kennedy_ON_ conditions (Fig 2B). Instead, slow growth was possibly due to lipostatic or proteostatic challenges.

To confirm that the rewired strains produced PE and PC, we used lipid thin‐layer chromatography (TLC). In agreement with their ability to grow, all rewired strains efficiently produced both lipids (Fig 2C). Differences in overall lipid profiles were nevertheless obvious. For example, phosphatidylinositol (PI) levels appear to be higher in most rewired strains. This increase could be a compensatory mechanism to deal with altered PE/PC ratios. Moreover, in the strain expressing Psd targeted to the MIM, PS levels were consistently low, suggesting that PS is most efficiently metabolized into PE in these conditions. Altogether, the growth and lipid profile of rewired yeast strains indicate that yeast cells can tolerate topological rewiring of their PE and PC biosynthesis pathways. This suggests that cells must have mechanisms to re‐distribute lipids from these non‐native locations to other membranes around the cell, or membrane tethers that might promote activity of the enzymes *in trans*, as well as mechanisms to cope with lipid imbalance.

### A transposon mutagenesis screen to identify genetic components essential for survival of yeast with rewired lipid biosynthesis

**Figure 3:**
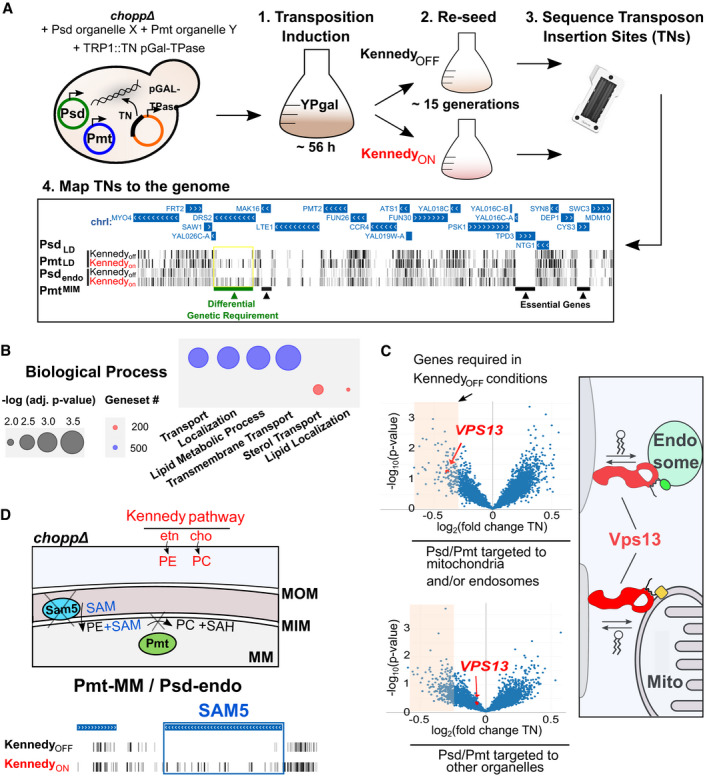
Transposon mutagenesis screens to identify genes required for adaptation and lipid trafficking in the rewired strains Outline of the SATAY screening procedure. Cells expressing plasmids containing the PE‐ and PC‐synthesizing enzymes and a plasmid harboring the galactose inducible transposase (TPase) and a transposon (TN) disrupting the *TRP1* gene were grown for ~ 56 h in galactose‐containing medium to induce transposition (step 1). Cells were inoculated in synthetic medium lacking tryptophan in either Kennedy_ON_ or _OFF_ conditions, grown for several generations (step 2) and harvested for DNA extraction and sequencing of transposon insertion sites (TNs) (step 3). TNs are mapped to the genome to identify genes that become required under certain rewired conditions (step 4). An illustrative example of TN insertions of four libraries visualized in the UCSC genome browser highlights a genetic requirement for *DRS2* (green bar) in Kennedy_OFF_ conditions.GO term enrichments in the top 200 (red) and top 500 (blue) most variable genes (defined as genes with the highest standard deviation of TN insertions across libraries) identified using the YeastMine web server. Significance was determined using Holm–Bonferroni corrected *P*‐values with a cut‐off of 0.05, the size of the circles is inversely proportional to the *P*‐values (i.e., bigger circles indicate higher significance).
*VPS13* is required in Kennedy_OFF_ conditions when chimeric enzymes are targeted to mitochondria or endosomes. Volcano plots show the fold change of number of transposon insertions per gene of libraries grown in Kennedy_OFF_ versus _ON_ conditions. This comparison included all six libraries where the enzymes are targeted to endosomes and/or mitochondria (top left panel) or six libraries with enzymes targeted to other organelles (bottom left panel). Data points for *VPS13* are highlighted in red. Schematic illustrating Vps13‐mediated lipid transport at endosomes and mitochondria contact sites (right panel).Schematic (top panel) illustrating the requirement for SAM transport across the mitochondrial inner membrane by Sam5 for PC production by Pmt in the mitochondrial matrix (MM). Lipids can be produced by the Kennedy pathway (red) independent of Sam5 activity. Transposon insertion maps generated in the UCSC genome browser for *SAM5* for the library where PC is produced in the MM in either Kennedy_ON_ or _OFF_ conditions (bottom panel). Source data are available online for this figure.

To identify the genetic components necessary to handle lipid imbalances and reroute lipid trafficking in the rewired strains, we performed genetic screens using SAturated Transposon Analysis in Yeast (SATAY, Fig 3A) (Michel *et al*, 2017; preprint: Michel *et al*, 2019). Briefly, we generated libraries consisting of millions of independent transposon insertion mutants. The transposon libraries for a given genotype were then grown for several cycles in the presence or absence of ethanolamine and choline (Kennedy_ON_ or Kennedy_OFF_) to assess the growth of the mutants in the rewired conditions. Next‐generation sequencing then allows mapping and quantifying the transposon insertion sites (TNs) across the genome. The number of transposons and associated sequencing reads in each ORF is then used to evaluate the fitness of the mutants in the given conditions.

We generated a total of 24 libraries comprising 12 rewired strains grown in both Kennedy_ON_ and _OFF_ conditions. Transposition insertion sites mapping across the entire yeast genome (SData Fig 3, dataset 1) can be viewed in the genome browser (http://genome‐euro.ucsc.edu/s/benjou/rewiring_paper, Suppl. Data 1). Additionally, we compared different sets of libraries against each other (e.g., individual libraries against all other libraries and libraries with a common condition, such as the expression of the same chimeric enzyme, or Kennedy_ON_ vs. _OFF_, against other libraries). We generated multiple interactive volcano plots available for browsing (SData Fig 3, dataset 2 and https://kornmann.bioch.ox.ac.uk/satay/rewiring) in which we plotted the fold change of the average number of transposons per gene of a test set and a reference set against a *P*‐value associated with this difference to identify genes that provide a fitness advantage or disadvantage in certain libraries.

**Figure EV1:**
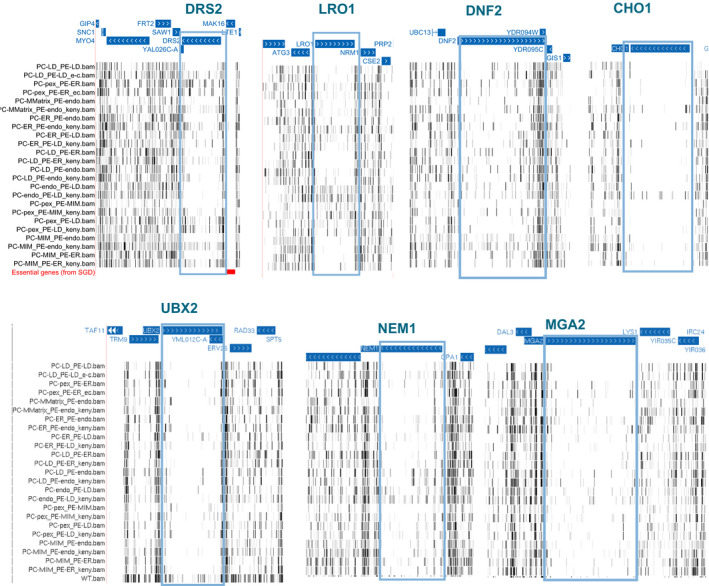
Lipid metabolism and lipid flippases genes are required for adaptation in specific rewired conditions Transposon insertion maps of the *DRS2*, *LRO1*, *DNF2*, *NEM1*, and *MGA2* genomic loci generated in the UCSC genome browser for libraries of strains expressing the chimeric Psd and Pmt enzymes to produce PE and PC at the indicated cellular locations in the *choppΔ* background. “KennedyON” refers to libraries that were grown in media supplemented with 10 mM ethanolamine and choline.

We performed hierarchical clustering of the libraries according to their pattern of transposon insertion in each gene (Appendix Fig S14). Libraries with the same genotypes, in the Kennedy_ON_ and _OFF_ conditions, cluster as nearest neighbors. Since these libraries are replicates grown under different conditions, and the number of transposons in most genes is unaffected by the activity of the Kennedy pathway, this clustering may be dominated by technical variation. Nevertheless, independent libraries generated in strains expressing the same PE‐ or PC‐producing enzymes also tended to cluster together. In some cases, the location of the PE‐producing enzyme dominates in the clustering (e.g., PE‐LD), and in other cases, it is that of the PC‐producing enzyme (e.g., PC‐pex). Yet in general, there is no pattern based on the location of one or the other enzyme. Overall, this clustering implies that the rewiring dominates in determining fitness and genetic requirements rather than the availability of choline and ethanolamine. To identify genes required to adapt to the rewired conditions, we first sought to filter for those with the most variable number of transposons across the whole dataset, using the standard deviation (SData Fig 3). This subset may omit genes that become essential in only one or very few conditions yet is an unbiased way of identifying genes that generally affect fitness in rewired conditions. We selected the top 200 and top 500 most variable genes and searched for GO term enrichment using the YeastMine web server (Balakrishnan *et al*, 2012). Remarkably, these genes were enriched for GO terms related to lipid homeostasis (lipid transport, localization, and metabolism) and other processes such as intracellular transport (Fig 3B). Particularly striking examples of variable genes are *DRS2*, *DNF2*, and *LRO1*, which are completely devoid of transposons in some conditions (Fig EV1). *DRS2* and *DNF2* both encode phospholipid translocases required for maintaining phospholipid asymmetry (Sebastian *et al*, 2012). They may be required to maintain proper lipid bilayer distribution in rewired strains. Lro1 is an acyltransferase that converts DAG to triacylglycerol (TAG) using phospholipids as acyl chain donors (Barbosa *et al*, 2019). Thus, Lro1 may be required to degrade excess membrane. Importantly, these genes had variable but non‐similar patterns of transposon coverage across libraries, indicating that it was not a common condition (e.g., Kennedy_ON_ or _OFF_) that made them more or less required.

Intriguingly, the lipid transporter Vps13, even though never completely essential, appears to be consistently less covered in Kennedy_OFF_ conditions (Appendix Fig S15) in libraries in which Psd and Pmt are targeted to mitochondria and/or endosomes, organelles to which this lipid transporter localizes (Lang *et al*, 2015; Park *et al*, 2016; John Peter *et al*, 2017) (Fig 3C, Appendix Fig S15). Together, these results suggest that Vps13 may have a more prominent role at these organelles in Kennedy_OFF_ conditions and underlines that lipid transporters could become limiting when rewiring involves their native site. Notably, we find that the inner mitochondrial S‐adenosylmethionine (SAM) transporter *SAM5* (Marobbio *et al*, 2003) is essential in Kennedy_OFF_ conditions when PC synthesis is targeted to the mitochondrial matrix (Fig 3D). SAM is the methyl group donor for the conversion of PE to PC, and thus, SAM is necessary in the matrix for PC production, making Sam5 indispensable. This finding excludes the possibility that growth of this strain is attributable to PC production by a small fraction of mistargeted enzymes. Thus, our rewiring approach can identify genes that maintain cell viability in rewired conditions and these genes are, as expected, enriched for lipid and membrane‐related processes.

### Similar transposon insertion patterns across rewired conditions reveal clusters of functionally related genes

**Figure 4:**
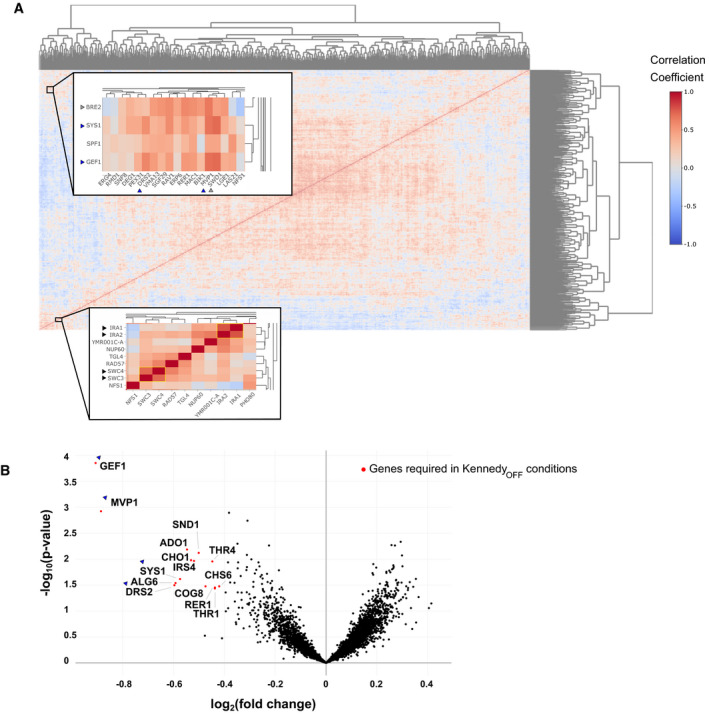
Clustering of gene correlations across libraries reveal gene groups that work together to handle rewired conditions Hierarchical clustering of the Pearson correlation coefficients of transposon insertion profiles for gene pairs. Insets show clustering of the *IRA1*/*IRA2* and *SWC3*/*SWC4* gene pairs (bottom inset, black arrows) and high correlation of the *SYS1*/*GEF1*/*SPF1* cluster with *DRS2* and *MVP1* (top inset, blue arrows).Volcano plot comparing the number of transposon insertions per gene of all 12 libraries in Kennedy_ON_ and _OFF_ conditions. Genes significantly required in Kennedy_OFF_ conditions are highlighted in red (*P*‐value < 0.05, log_2_[fold change] < −0.5). Genes highlighted in the clusterogram in (A) (top inset) are indicated with blue arrows.

Previous genome‐wide analyses have shown that genes that are required under the same set of conditions are usually part of the same biological process, pathway or protein complex (Costanzo *et al*, 2016). Therefore, we asked if we could identify genes that have similar patterns of transposon insertion across different rewired libraries. We selected the most variable genes and computed the Pearson correlation coefficient for pairs of genes across our libraries and performed hierarchical clustering on the correlation matrix (Fig 4A, SData Fig 3, dataset 3).

We find clustering of highly correlated gene pairs consistent with their known function and/or profile similarities computed in the whole‐genome genetic interaction map (Costanzo *et al*, 2016) (Fig 4A). Examples include *IRA1* and *IRA2* (Tanaka *et al*, 1990), paralogs that negatively regulate the RAS‐cAMP pathway and *SWC3* and *SWC4*, both components of the chromatin remodeling SWR Complex (Mizuguchi *et al*, 2004) (Fig 4A, black arrows). We also find correlation between *BRE2* and *SWD1* (Fig 4A, grey arrows), both members of the same histone methylation complex (COMPASS) (Krogan *et al*, 2002).

A striking example, also consistent with the Costanzo dataset (Costanzo *et al*, 2016), is the cluster containing *SYS1*, *SPF1*, *GEF1*, and *MVP1* (Fig 4A and B, blue arrows). We found that this cluster became essential in most Kennedy_OFF_ conditions, as shown by a volcano plot in which libraries grown with and without ethanolamine and choline are compared against each other (Fig 4B). This implies that these genes function together and act redundantly with the Kennedy pathway to maintain cell viability in the rewired strains. Given their known roles in vesicular trafficking and protein sorting, it is possible that the Kennedy pathway is required for efficient vesicular trafficking in rewired conditions, or that vesicular trafficking is needed to redistribute lipids in the cell. Alternatively, vesicular trafficking may affect proper localization of other factors required for lipid distribution (e.g., Drs2). These examples demonstrate that the transposon insertion patterns across libraries can reveal genes that function together to maintain cell viability upon lipostatic challenges, in an unbiased and genome‐wide fashion.

### Rewiring highlights the importance of transcriptional and post‐transcriptional responses

**Figure 5:**
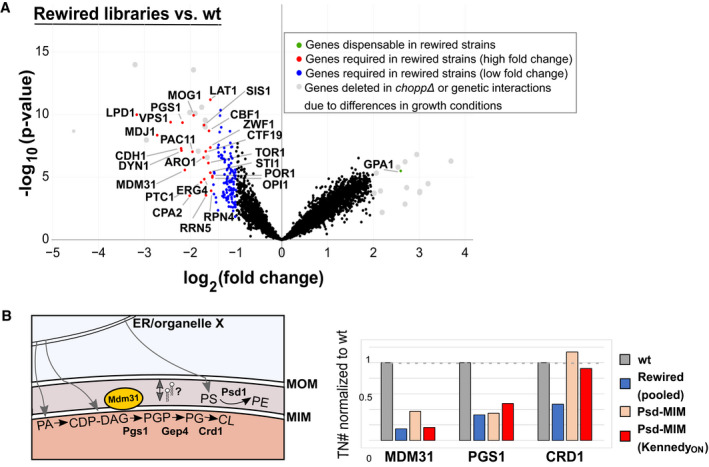
Differential genetic requirements common to rewired libraries Volcano plot comparing the number of transposon insertions per gene of all 24 rewired libraries and two wild‐type (wt) libraries. Genes that are significantly (*P*‐value < 0.05) required (red, log_2_[fold change] < −1.5; blue, log_2_[fold change] < −1) or dispensable (green, log_2_[fold change > 2]) in the rewired libraries are highlighted. Genes depicted in grey are essential due to differential growth requirements in the preparation of the two wt libraries compared to the rewired strains, or correspond to genes deleted in the rewired strains.Requirement for mitochondrial lipid transport and biosynthesis genes in rewired libraries. Left panel, schematic depicting PE and cardiolipin (CL) synthesis in mitochondria. The inner mitochondrial putative lipid transporter Mdm31 is depicted in yellow. Gray arrows indicate the need for lipid transport from the ER or other organelles to mitochondria and transport between the MOM and MIM. Right panel, transposon numbers (normalized to wt libraries) for the indicated (set of) libraries for *MDM31*, *PGS1*, and *CRD1*. Source data are available online for this figure.

**Figure EV2:**
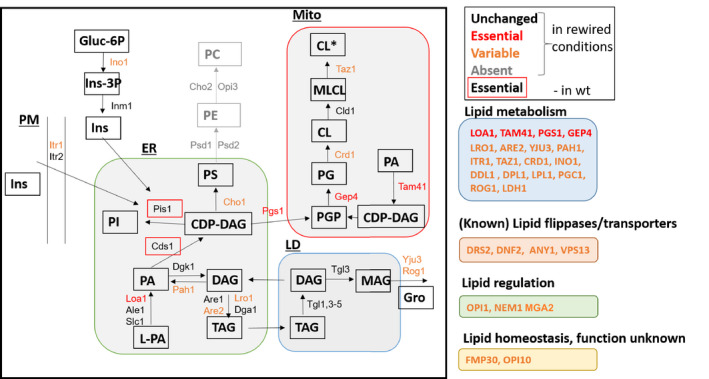
Requirement for lipid metabolic genes in rewired yeast Schematic illustration of lipid synthesis pathways in yeast. Genes that are essential or absent in all libraries are depicted in red or gray, respectively. Genes variably required in one or more rewired libraries, as assessed by manual inspection or volcano plots, are depicted in orange. Genes that are essential in wild‐type conditions are boxed in red.

Besides unique adaptations to specific conditions, we postulated that rewired strains have common strategies to deal with general phospholipid and membrane stress. To identify such genes, we compared the rewired libraries generated in this study, with two previously generated wild‐type libraries (Fig 5A) (preprint: Michel *et al*, 2019). Expected changes in transposon insertion profiles were associated with technical differences in library generation, in the present and previous studies (e.g., genes for histidine, leucine, tryptophan, and adenine prototrophy, that were used for selection) (Fig 5A, grey dots). Besides these, striking differences were found in several genes involved in transcriptional adaptation (Fig 5A, SData Fig 5). One of the best hits is *OPI1*, a transcriptional repressor for a variety of lipid biosynthesis genes under the control of the Ino2‐Ino4 transcription activation complex (e.g., *INO1*, *CHO1*, *ITR1*, genes of the Kennedy pathway) (Henry *et al*, 2012). *OPI1* is required in all rewired libraries, indicating an increased need for transcriptional regulation of phospholipid biosynthesis. Another example is the transcription factor *CBF1*, which is implicated in enhancing Ino2‐Ino4 transcriptional activation (Shetty & Lopes, 2010), mitochondrial respiration, and repressing ceramide biosynthesis (DeMille *et al*, 2019). Examples of genes required for growth only in specific libraries, include *NEM1*, a phosphatase controlling phospholipid biosynthesis (Siniossoglou *et al*, 1998), and *MGA2*, a transmembrane transcription factor involved in sensing membrane saturation (Covino *et al*, 2016) (Figs EV1 and EV2). These results suggest that distinct rewired conditions cause both common and unique membrane challenges that require a combination of adaptive responses.

In addition to transcriptional adaptation to the altered lipidome, we observed many additional changes in the genetic requirement of rewired strains (Fig 5A, SData Fig 5). These include an increased reliance on proteostasis regulators (*RPN4*, *MDJ1*), and on otherwise non‐essential lipid biosynthesis pathways, such as cardiolipin (CL) synthesis (*PGS1*, *CRD1*) and the final step of ergosterol synthesis (*ERG4*; Fig 5A). The mitochondrial inner membrane‐targeted Psd rescues the requirement for CL synthesis in the transposon screen, consistent with previously published work highlighting redundant functions for PE and CL in mitochondria (Fig 5B) (Gohil *et al*, 2005; Joshi *et al*, 2012). In contrast, synthesis of the cardiolipin precursor phosphatidylglycerol (PG) is required in all libraries (Fig 5B). Moreover, analysis of genes involved in lipid biosynthetic pathways suggest a variable requirement for the enzymes involved in the synthesis of phospholipids such as PA, PI, PE, CL, and the neutral lipid TAG (Fig EV2).

Interestingly, transposons in the gene encoding the inner mitochondrial membrane protein Mdm31 are tolerated in wild‐type libraries, but not in the rewired strains. *MDM31* genetically interacts with *PSD1* and is crucial for mitochondrial lipid homeostasis (Miyata *et al*, 2017). Notably, PE synthesis at the mitochondrial inner membrane by our chimeric construct (resembling mitochondrial localization of Psd1) only marginally rescues the number of transposons in the *chopp*Δ background (Fig 5B), implying that *mdm31* mutants are particularly sensitive to altered PE/PC levels and distribution in the absence of the endogenous CDP‐DAG pathway. Mdm31 harbors a Chorein‐N motif, a signature domain found in lipid transport proteins including Vps13 (Levine, 2019). Thus, it is tempting to speculate that the requirement for *MDM31* in the rewired conditions is due to a requirement for lipid transport across the mitochondrial intermembrane space.

### Csf1 is required for lipid adaptation in rewired conditions

**Figure 6:**
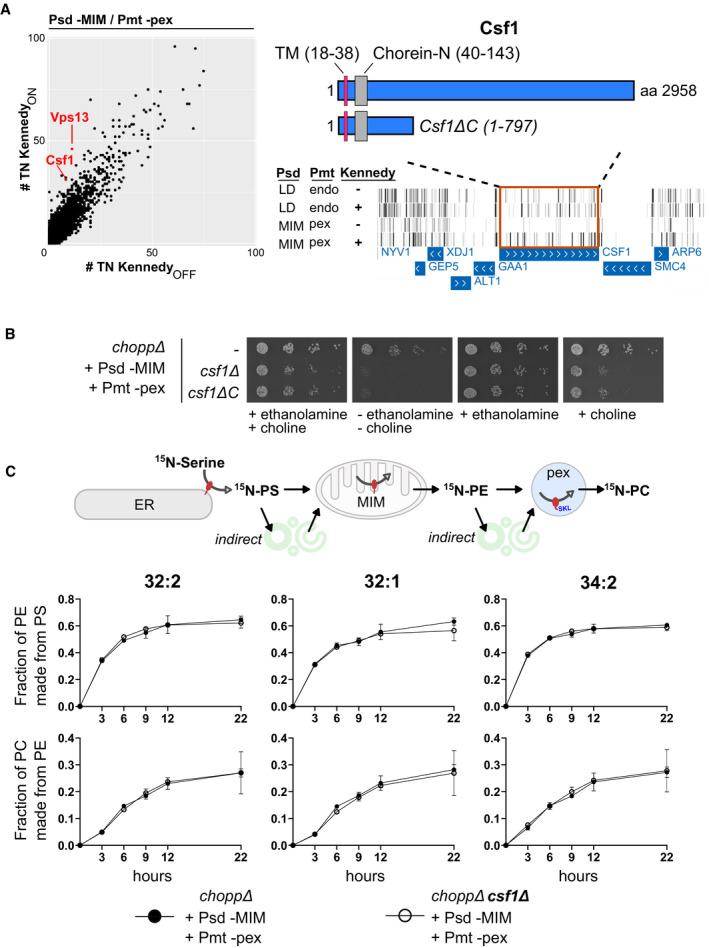
Csf1 is required specifically in the Psd‐MIM/Pmt‐pex rewired condition Left: comparison of the number of transposon insertions per gene (dots) for libraries with PE synthesis targeted to the MIM and PC synthesis targeted to peroxisomes, in Kennedy_ON_ and _OFF_ conditions. *CSF1* and *VPS13* (highlighted in red) are more required in Kennedy_OFF_ conditions. Right: Transposon insertion profile displayed in the UCSC genome browser for the indicated libraries for the genomic region of *CSF1*. Bars correspond to transposons, rows to libraries. The boundaries of the full‐length Csf1 protein, the truncated Csf1ΔC protein, the Chorein‐N motif and the transmembrane (TM) domain are indicated.Five‐fold serial dilutions of strains of the indicated genotypes grown at 30°C on SD medium supplemented with 10 mM ethanolamine and/or 10 mM choline. Growth assays are representative images of biological replicates.PS and PE transfer assay. Rewired strains were pulse labeled with ^15^N‐serine. Lipids were extracted at the indicated time points and measured by mass spectrometry. Graphs depict quantification of the appearance of labeled species of the indicated phospholipids normalized by the amount of its precursor lipid.

**Figure EV3:**
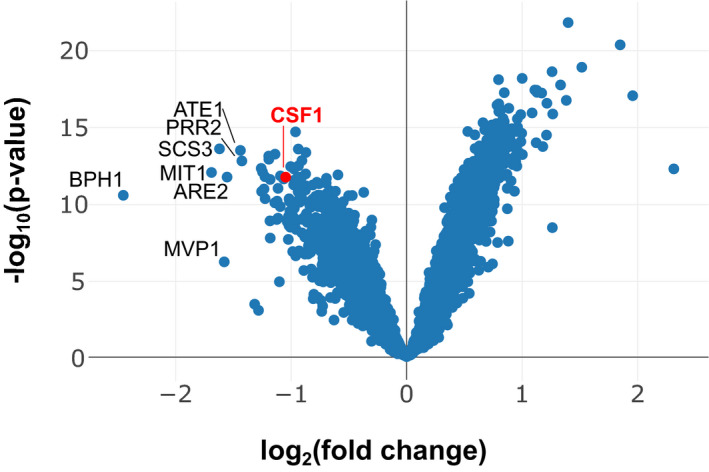
The requirement for Csf1 is specific to the Psd‐MIM/Pmt‐pex library grown in Kennedy_OFF_ conditions Volcano plot comparing the number of transposon insertions per gene in the Psd‐MIM / Pmt‐pex Kennedy_OFF_ condition versus all other libraries. *CSF1* is highlighted in red.

As we relocalized lipid synthesis to distinct compartments, we expected that LTPs might become particularly necessary to distribute lipids in specific rewired Kennedy_OFF_ conditions. Genes satisfying this condition were scrutinized based on previous literature and structural predictions to identify LTP candidates. Csf1 emerged as a promising hit, as a HHpred analysis (Zimmermann *et al*, 2018) predicts an N‐terminal Chorein‐N motif, homologous to that found in Vps13, Atg2, and other potential lipid transporters including Mdm31 (Fig 6A) (Levine, 2019; Li *et al*, 2020). Csf1 is a large protein like Vps13 and Atg2. They are respectively the 10^th^, 5^th^, and 92^nd^ largest *S cerevisiae* polypeptides. While Vps13 and Atg2 are soluble proteins, Csf1 bears an N‐terminal transmembrane domain. Transposons targeting the *CSF1* ORF were strongly depleted in Kennedy_OFF_ conditions (Figs 6A and EV3) in the strain where Psd is targeted to the MIM and Pmt production to peroxisomes (hereafter referred to as Psd‐MIM/Pmt‐pex). However, contrary to *VPS13*, which becomes required for growth in most libraries involving PE or PC synthesis at endosomes or mitochondria (Figs 3C and 6A), the requirement for *CSF1* was specific to the Psd‐MIM/Pmt‐pex conditions.

To validate the screen results, we generated a strain in which *CSF1* was deleted in the *choppΔ* Psd‐MIM/Pmt‐pex background. As expected, *CSF1* was required for growth in Kennedy_OFF_ conditions (Fig 6B), but dispensable when PE production was redirected to endosomes and PC production to lipid droplets (Appendix Fig S16). We observed variable phenotypes when *CSF1* was deleted with different gene replacement cassettes. This phenomenon is likely attributable to interference with *CSF1*'s direct neighbor, *GAA1*. GAA1 encodes an essential component of the glycosylphosphatidylinositol (GPI) anchoring machinery. Previous screens utilizing full *csf1* deletion alleles found that these strains had a phenotype related to that of mutants with defective GPI protein anchoring (Čopič *et al*, 2009; Jonikas *et al*, 2009; Costanzo *et al*, 2010). It is therefore most likely that the slow growth and the phenotype similarities are due to inhibition of *GAA1* by *CSF1* deletion. To avoid interference with neighboring genes, we truncated (rather than deleting) *CSF1* after amino acid 797. This allele (Csf1∆C) recapitulated the growth phenotype of the *CSF1* deletion in the rewired Kennedy_OFF_ condition and was therefore used for further loss‐of‐function experiments.

**Figure EV4:**
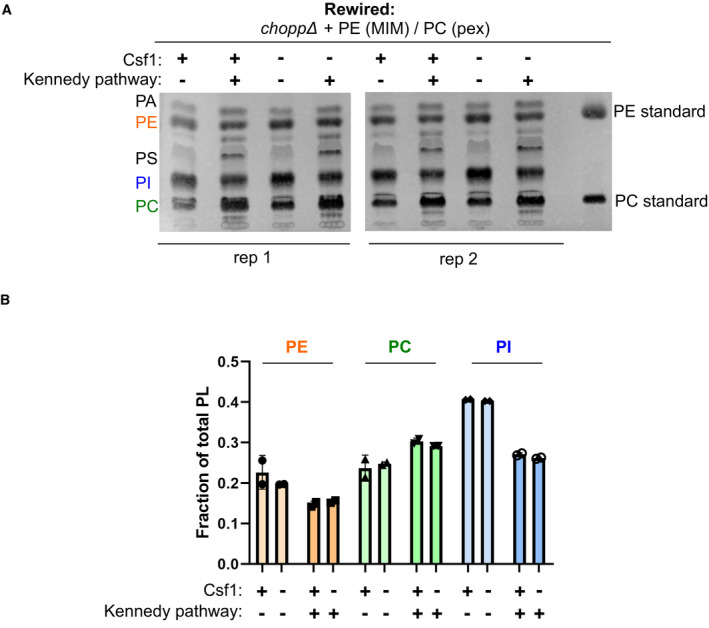
The absence of Csf1 in rewired conditions does not affect steady state PE or PC production Thin‐layer chromatography (TLC) analysis of the steady‐state lipid profiles of the indicated strains grown in Kennedy_OFF_ or Kennedy_ON_ conditions (+10 mM ethanolamine and choline).Quantification of the fraction of PE, PC, and PI of total phospholipids measured by TLC analysis shown in A. Quantification was done using Fiji/ImageJ software as described in the Materials and Methods.

**Figure EV5:**
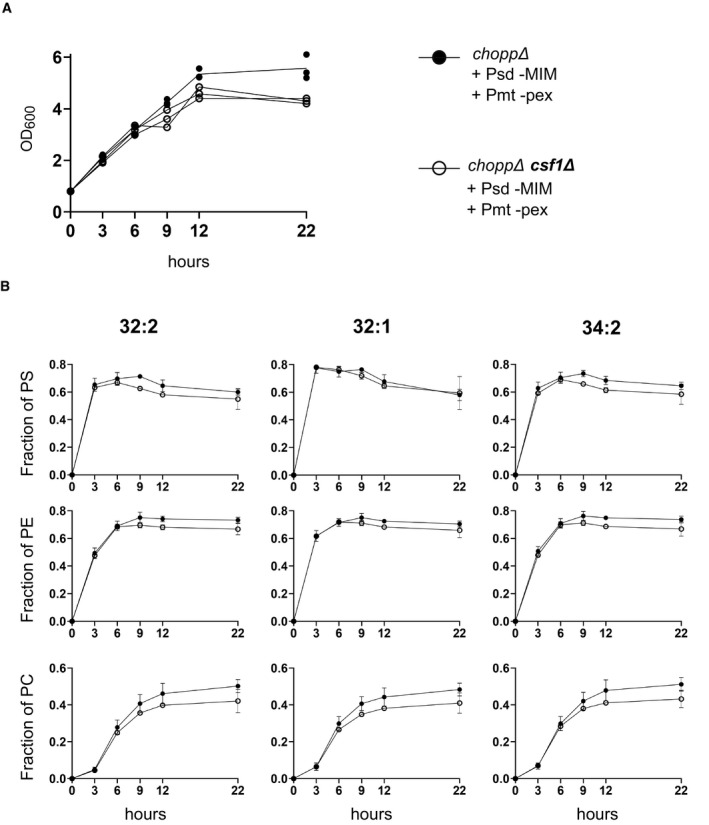
Rate of growth and ^15^N incorporation in PS, PE, and PC during the experiment shown in Fig 6C OD_600_ measurements of the indicated genotypes at different time points taken during the ^15^N‐serine labeling experiment.Line plots depict quantification of the amount of indicated ^15^N‐labeled species normalized by the amount of its corresponding unlabeled species. Source data are available online for this figure.

We found that ethanolamine supplementation promotes growth of rewired *csf1* mutants better than choline (Fig 6B), indicating that homeostasis of PE, and not PC, likely underlies the growth defect of the *csf1* mutant in the rewired conditions. Yet, no detectable change in phospholipid abundance could be uncovered by thin‐layer chromatography of whole cell lipid extracts of *csf1∆C* cells (Fig EV4). The defect of *csf1* mutants in one specific rewired condition suggested that these cells had a defect in transporting PE made in the mitochondria to the peroxisome for conversion to PC. This transport could be direct or involve any number of steps. To assess the flux of PE made by the MIM‐targeted Psd, and converted to PC by the peroxisome‐targeted Pmt, we pulse‐labeled the rewired strain with ^15^N‐serine and monitored the production of PS, PE, and PC over time. The appearance of ^15^N‐labeled PE and PC should reflect on the activity of the Psd and Pmt enzymes as well as on the lipid transport rates between them. We observed that the presence of ethanolamine and choline likely inhibited the CDP‐DAG pathway, preventing acquisition of meaningful data. At the same time, *csf1* mutants do not grow well in the absence of these compounds. To circumvent these limitations, ^15^N‐Serine labeling was performed immediately after withdrawal of ethanolamine and choline. Growth of the mutants was only slightly affected at later time points, presumably when ethanolamine and choline became limiting (Fig EV5A). This slower growth was reflected in a slightly slower ^15^N incorporation in PS, PE, and PC (Fig EV5B, SData Fig EV5). Surprisingly, the synthesis rates of ^15^N‐PE and ^15^N‐PC, when normalized to the abundance of their precursors (*i.e*., ^15^N‐PS for ^15^N‐PE and ^15^N‐PE for ^15^N‐PC) were identical in wild type and *csf1∆C* cells, indicating that the growth defect of the rewired strain in the absence of Csf1 is not likely due to decreased direct or indirect transport of PE from its site of synthesis to its site of conversion to PC (Fig 6C).

**Figure 7:**
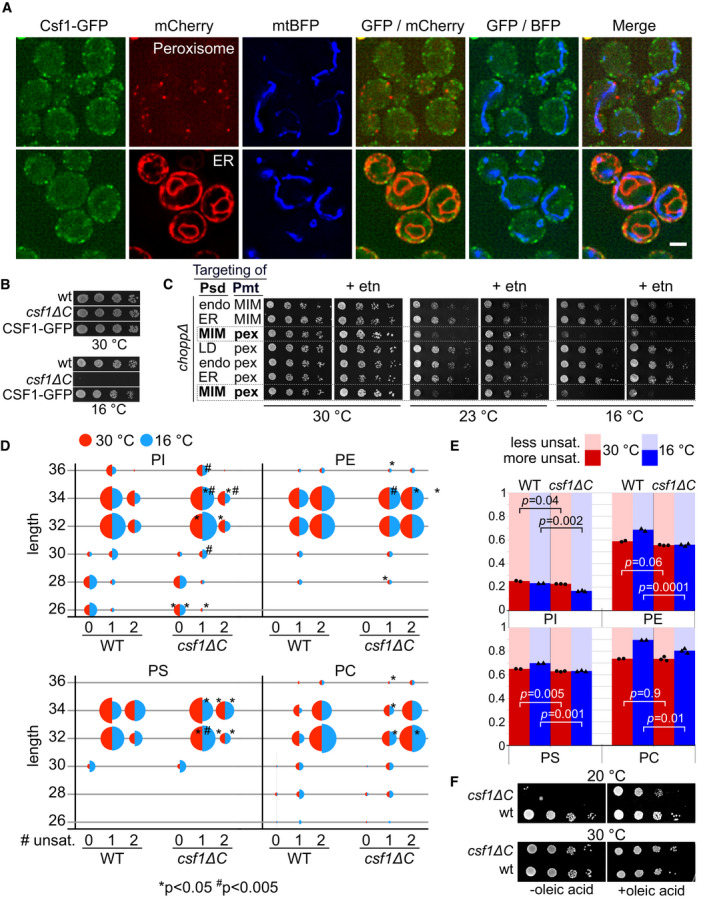
Csf1 is involved in phospholipid remodeling during cold stress Top: Co‐localization of Csf1‐GFP cells in log phase with genomically integrated Pex10‐mCherry (peroxisome marker) and mtBFP plasmid (mito marker). Bottom: Co‐localization of log growing Csf1‐GFP cells with mCherry‐Ubc6 (tail‐anchor) (ER marker) and mtBFP plasmid (mito marker). Scale bar, 2 µm.Five‐fold serial dilutions of strains of the indicated genotypes grown on YPD plates at 30°C or at 16°C. Growth assays are representative images of biological replicates.Five‐fold serial dilutions of strains of the indicated genotypes grown at indicated temperatures, in the presence or absence of 10 mM ethanolamine (etn).Bubble plots depicting the changes in abundance for each phospholipid class, classified based on the fatty acid length and degree of unsaturation, in wild‐type (WT) and *csf1ΔC* cells grown at 30°C (red‐half) or at 16°C for 14.5 h (blue‐half). Significant changes for WT versus *csf1ΔC* at 16°C or at 30°C are indicated (*P < 0.05, ^#^ *P* < 0.005). *P*‐values were calculated with the two‐sample *t*‐test.Bar graphs depicting the fraction of more saturated species (defined as fatty acid chains with one double bond for ≤30 carbon chains or with two double bonds for ≥32), and less unsaturated species (defined as fatty acid chains with zero double bond for ≤30 carbon chains or with one double bond for ≥32) for each phospholipid class with different head groups. Bars represent the means of two or three biological replicates (individual measurements are shown as points and triangles). *P*‐values are calculated with a two‐sample *t*‐test.Ten‐fold serial dilutions of indicated strains on YPD plates with or without 0.1% oleic acid at 20°C or at 30°C. Source data are available online for this figure.

To examine Csf1's subcellular localization in wild‐type cells, we genomically tagged its C terminus with GFP. To test whether the Csf1‐GFP allele is functional, we took advantage of the cold‐sensitive phenotype of *csf1* mutants (Tokai *et al*, 2000). While *csf1∆C* cells showed a severe growth phenotype at 16°C, cells expressing Csf1‐GFP grew indistinguishably from wild‐type cells, indicating that the GFP‐tagged protein was functional (Fig 7B). Csf1‐GFP localized to puncta that co‐localized with an ER marker and are predominantly found near the cell periphery (Fig 7A). This localization is consistent with the presence of a short (21 amino acids) transmembrane domain at the N terminus of the protein (Sharpe *et al*, 2010). We found no evidence for obvious co‐localization with either mitochondria or peroxisomes and no indication of localization at contact sites between the ER and these organelles (Fig 7A). Given that Csf1 affects neither steady‐state lipid class abundance nor transport to mitochondria and peroxisomes and that Csf1 does not appear to localize at these organelles, we conclude that Csf1 function is likely affecting lipid homeostasis in more indirect fashions, which may particularly affect strains with PE made in mitochondria and PC made in peroxisomes.

Cold sensitivity is the only well‐documented phenotype of *csf1* mutants (Tokai *et al*, 2000). While most cold‐sensitive mutations affect lipid‐independent processes like RNA metabolism (Noble & Guthrie, 1996), yeast cells also need to adapt their lipidome in cold conditions to maintain membrane fluidity (Klose *et al*, 2012). This raised the possibility that the Psd‐MIM/Pmt‐pex rewired condition may yield a lipidome that is ill‐adapted to cold and that this inadequacy is synergistic with the effect of *csf1* mutation. Indeed, we find that Psd‐MIM/Pmt‐pex rewiring causes a cold sensitivity at both 23 and 16°C and the phenotype at 23°C is rescued by ethanolamine supplementation (Fig 7C, dashed boxes). This cold‐sensitive lipidome of Psd‐MIM/Pmt‐pex cells may therefore explain the synthetic lethality with *CSF1*.

We thus wondered if loss of *CSF1* interferes with lipidomic adaptation to cold in cells with normal PE and PC biosynthesis. We quantified the levels of different phospholipid species by mass spectrometry in cells grown at 16°C for 14.5 h. We found that cells adapt their lipidome upon cold treatment by generally shortening the acyl chains and increasing the level of unsaturation (Fig 7D, SData Fig 7), consistent with previous knowledge (Klose *et al*, 2012). Interestingly, this adaptive response was blunted in *csf1∆C* cells. Quantifying the degree of unsaturation (0 vs 1 double bond for lipids with ≤ 30 carbons, or 1 vs. 2 double bonds for lipids with ≥ 32 carbons) revealed that while wild‐type cells undergo fatty acid remodeling, *csf1* mutants failed to do so (Fig 7E). These data suggest that a defect in lipid remodeling from saturated to mono‐ and mono‐ to di‐unsaturated species underlies the cold‐sensitive phenotype of *csf1* mutants. To test this hypothesis, we reasoned that manipulating the level of unsaturation in *csf1* mutants might rescue their cold sensitivity. Increased lipid unsaturation can be achieved by feeding cells with oleic acid (C18:1) in the culture medium (Grillitsch *et al*, 2011). While oleic acid supplementation was not able to restore the growth of *csf1* mutants at 15°C (Appendix Fig S17A), a substantial growth rescue was observed at an intermediate temperature of 20°C (Fig 7F, Appendix Fig S17A and B, SData Appendix Fig S17). Thus, feeding oleic acid partially rescues the cold sensitivity of *csf1* mutant cells, indicating that lipidomic defects underlie this phenotype.

## Discussion

Here, we show that synthesis of the phospholipids PE and PC can be re‐localized to almost any combination of organelles, supporting yeast growth without the need for the Kennedy pathway. Importantly, conclusions of this study are contingent on the proper targeting of the lipid‐modifying enzymes. While we have observed incomplete targeting of some constructs (peroxisome‐ and LD‐targeted Pmt), in the case of the matrix‐targeted Pmt, growth restoration is dependent on the mitochondrial SAM transporter Sam5, indicating that growth restoration is due to the activity of the properly targeted enzyme. Moreover, the uniqueness of transposon insertion profiles indicates that the localization of the enzymes matter and therefore that the targeted fraction dominates over any mistargeted enzymes.

The viability of all rewired strains suggests bidirectional transport of lipids between the organelles assayed here, which include mitochondria, ER, peroxisomes, lipid droplets, and endosomes. Bidirectional lipid transport could be direct (between the two organelles), indirect (using one or more intermediary organelle(s)), or a combination thereof. Regardless of the precise route(s) taken, our data suggest that the lipid transport network is complex and organelles considered to be mere lipid importers might also be capable of export.

What molecular components allow yeast cells to survive rewiring and adapt to changes in organelle membrane composition? We identified genes required for survival by mapping genome‐wide differential genetic requirements in the rewired strains using transposon mutagenesis. The enrichment for genes involved in lipid metabolism, lipid trafficking, proteostasis, and vesicular trafficking supports an important role for these pathways in maintaining functional membranes and organelles upon perturbations of the lipidome. We anticipated that cellular adaptation to the rewiring conditions necessitates sensors of membrane composition and stress. Membrane‐associated proteins have been identified that sense and respond to lipid packing (Covino *et al*, 2016) and phospholipid composition (Henry *et al*, 2012). We find the ER lipid packing sensor Mga2 to be required in some of the rewired conditions, implying that the location of phospholipid synthesis can alter the properties of the ER membrane, thus necessitating Mga2 activation. On the other hand, Opi1, which senses the amount and protonation state of PA, is required in all rewired strains. It has been observed that PA protonation does depend not only on intracellular pH but also on membrane composition, and in particular on the presence of PE head groups (Kooijman *et al*, 2005; Young *et al*, 2010). Possibly, Opi1 can sense altered PA levels or PE/PC ratios via changes in PA protonation.

In addition, genes encoding proteins with putative lipid transport domains became essential in the rewired conditions. In particular, we identified three Chorein‐N motif genes, *MDM31*, *VPS13*, and *CSF1*, that each became required under specific conditions. *VPS13*, which is implicated in lipid transport at mitochondria and endosomes, appeared to be particularly important in Kennedy_OFF_ conditions in libraries where PE and PC synthesis are redirected to either of these organelles. It is therefore likely that lipid shuttling by Vps13 allows cells to maintain lipid homeostasis and distribute lipids at these organelles.

Csf1, required in a specific rewired condition (Psd‐MIM/Pmt‐pex), was neither directly influencing the global lipidome nor lipid transport rates between the PE‐ and PC‐producing enzymes. Instead, our analyses reveal a connection between Csf1 and adaptation to cold. *csf1* mutants are unable to remodel their lipidome in cold stress by increasing the levels of fatty acid unsaturation in phospholipids. Importantly, Psd‐MIM/Pmt‐pex cells, like *csf1Δ*, were cold‐sensitive, and both their cold sensitivity and synthetic lethality with *csf1* were rescued by ethanolamine supplementation. Moreover, oleic acid supplementation, a treatment that affects the level of lipid unsaturation, substantially rescued the cold sensitivity of *csf1* mutants. Together, these findings indicate that defects in lipid homeostasis underlie *csf1* phenotypes.

Csf1 has homology to other LTPs like Vps13. Although the mechanism of Csf1's action on the lipidome remains to be investigated, our finding of a putative LTP involved in homeoviscous membrane adaptation opens a new avenue for exploring the relationship between lipid transport and membrane remodeling. An appealing speculation is that, in the cold, Csf1 might be required for lipid transport from other organelles to the ER to allow their remodeling via ER‐localized phospholipases and acyltransferases. Csf1 homologues in higher organisms are implicated in lipid storage (lpd‐3, *Caenorhabditis* *elegans*) (McKay *et al*, 2003) and endosomal trafficking (*tweek* and KIA1109, *Drosophila melanogaster* and *Homo Sapiens*, respectively) (Verstreken *et al*, 2009; Kane *et al*, 2019). Moreover, mutations in KIA1109 are associated with the Alkuraya‐Kučinskas syndrome, a severe neurological malformation disorder (Kane *et al*, 2019). Therefore, it will be important to understand whether and how Csf1 is involved in lipid transport.

Taken together, the extensive genetic interaction network reported here uncovers genes required to handle lipid imbalances and fulfill transport needs generated by specific rewiring conditions. The data will serve as a rich resource for future studies. They are accessible in the genome browser and can be interrogated via interactive plots that reveal significant differential genetic requirements. Correlation of the transposon insertion profiles allows identification of functionally related genes, and the conditions in which these are essential. We have shown that the data provide insight into general genetic requirements for handling membrane stress and can identify gene products that are essential in specific rewired conditions. Specifically, we uncovered a role for the conserved Chorein‐N motif‐containing protein Csf1 in lipidome remodeling upon cold treatment. This finding highlights a previously unappreciated role for Csf1 in lipid homeostasis and provides a promising starting point to elucidate its function and regulation in yeast and other organisms.

## Materials and Methods

### Yeast strains and plasmids

Yeast strains, plasmids, and primers used in this study are listed in Appendix Table [Link], [Link], respectively. Media recipes are in Appendix Supplementary Methods. Gene deletions were performed using genomic integration of PCR fragments (using oligos in Appendix Table S3) by homologous recombination following common procedures. We either used gene substitution by a selection marker using the Janke and Longtine toolboxes, as described previously (Longtine *et al*, 1998; Janke *et al*, 2004), or CRISPR‐Cas9‐mediated marker‐less deletion of the ORF (Laughery *et al*, 2015). For CRISPR‐Cas9‐mediated gene deletions, we supplied the cells simultaneously with three DNA fragments: (i) a Swa1‐linearized guide RNA (gRNA) expression vector (ii) annealed gRNA oligos with homology to the vector (iii) a repair DNA template to delete the ORF. The DNA template was generated by amplifying ~ 500 bp up‐ and downstream of the ORF and annealing the two fragments using extension overlap PCR as in (Walter *et al*, 2019). Colonies that had repaired the gRNA expression vector were selected. All gene deletions were confirmed by colony PCR.

Plasmids were generated using yeast gap‐repair cloning. For *PSD* and *PMT* plasmids, overlapping DNA fragments were transformed together with linearized pRS415‐TEFpr (Mumberg *et al*, 1995) using *Xba*I and *Xho*I. The pk*PSD* and aa*PMT* DNA sequences were ordered as gBlocks^®^ from IDT. The GFP sequence was amplified from pYM25 (Janke *et al*, 2004). Targeting signals were PCR‐amplified directly from yeast genomic DNA, except for peroxisomes and endosomes, where the signal was incorporated in the primer sequence and PCR‐amplified from plasmid pBK196, respectively. The *LEU2* markers of PSD plasmids were exchanged with *HIS3* (from pRS413) and/or *URA3* (from pRS416) to allow simultaneous expression of *PSD* and *PMT* (and other) plasmids. Briefly, pRS415‐based *PSD* plasmids were digested with *Age*I and transformed into yeast with *HIS3* or *URA3* PCR fragments with overlapping sequences to the vector. To generate the peroxisome marker, pRS416‐TEFpr (Mumberg *et al*, 1995) was digested with *Xba*I and *Xho*I and transformed with a mCherry‐SKL PCR product with overlapping sequences to the linearized vector. To generate the Lipid Droplet marker, the pRS413 vector was digested with *Sac*I and transformed along with two overlapping PCR fragments containing the *ERG6* ORF with its native promoter, and mCherry DNA sequences.

### Growth assays

Cells were grown to mid‐log phase in the appropriate synthetic defined (SD) drop‐out medium (to select for the plasmids), diluted, and spotted on appropriate plates. Five‐fold dilution series were used, with the first spot in each row corresponding to 2.5 µl of 0.2 OD_600_. In Kennedy_OFF_ conditions, plates were supplemented with 10 mM ethanolamine and choline. Cells were allowed to grow at 30°C (unless noted otherwise) for 2–3 days before imaging. Oleate (0.1%) was supplemented in solid medium in the presence of Tween80 (0.2%) by dropwise addition of a 10× stock solution in melted medium with constant stirring.

### Transposition library generation

Briefly, yeast strains were transformed with pBK626, the plasmid that contains a Maize Ac/Ds transposase under the control of a galactose promoter, TRP1 ORF disrupted by the miniDS transposon, and 600 bp sequence overlapping with TRP1 to facilitate homology‐directed repair of TRP1 after transposition. Individual clones were streaked on SC–HIS–URA–LEU (SC–HUL) and used to inoculate a pre‐culture in SC –HUL + 2% Raffinose + 0.2% Glucose at an OD_600_ of 0.2, which was grown until saturation. Saturated pre‐cultures were diluted to OD_600_ 0.2 in 400–800 ml YP + 2% Galactose medium and grown for ~ 56 h to induce transposition. After transposition induction, cultures were washed twice with fresh SC medium, inoculated at an OD_600_ ranging between 0.3 and 1 in 2 l of SC–LEU–TRP–HIS (SC –LTH) + 2% Glucose medium with and without supplemented ethanolamine and choline (10mM each) and grown until saturation. Cells were reseeded for a second growth round by diluting the saturated cultures in 300 ml of the appropriate medium and allowed to grow from an OD_600_ of 0.2 till saturation. Cells were harvested and frozen at −20°C until processed for DNA extraction and sequencing. To estimate the number of transposed clones, appropriate dilutions were plated on SC‐TRP and SC‐URA media at *t* = 0 and *t* = 56 h. Colony count at *t*_0_ served to estimate the background of TRP1^+^ clones due to spontaneous recombination of pBK626. The number of cycles that cells underwent after transposition was determined by counting the TRP1+ cells at the start and end of each re‐seed by plating appropriate dilutions on SC–TRP plates. Typically, we estimated between ~ 2 × 10^6^ and ~ 15 × 10^6^ individual transposition events per library and cells underwent between 13 and 15 cycles of growth in SC medium in Kennedy_ON_ and _OFF_ conditions.

### Sequencing

DNA was extracted from 0.5 g yeast pellets and processed for sequencing as described previously (Michel *et al*, 2017). Briefly, 2 µg of extracted DNA was processed by restriction digestion with *Nla*III and *Dpn*II, circularization, and PCR amplification. A distinct 8 bp illumina barcode was introduced in the PCR reverse primer for each library. Barcoded libraries were pooled and sequenced using the Nextseq High Output kit (Illumina) according to the manufacturer's guidelines.

### Sequencing data analysis

Reads were aligned to the yeast genome and processed further to detect individual transposon insertion sites as described previously (Michel *et al*, 2017). The *bed* and *wig* output files were uploaded to the UCSC genome browser to display transposition events and read counts, respectively. Transposon and read counts per gene were normalized to the total number of transposons/reads mapped for each library for subsequent analyses.

Hierarchical clustering of the libraries was performed in R (version 4.0.4) on the normalized transposon count per gene and clustered using the hclust() function using the ward.D method. The heatmap was visualized using the heatmap.2() function, using the dendrogram generated by hclust() to order the libraries and using a color scheme depicting the log_2_ fold change (TN count) for each gene in a library with respect to the mean TN count for that gene across libraries.

To identify genes that were variable across libraries, the StdDev was calculated for the normalized transposon count per gene across libraries. For GO term analysis, the 200 and 500 most variable genes (based on StdDev) were entered in the YeastMine webserver (Balakrishnan *et al*, 2012) and Holm–Bonferroni corrected GO term enrichments were computed.

Correlation clusterogram was computed as follows. Pearson correlation coefficients were calculated for the normalized transposon count per gene for most variable genes (determined by the StdDev as described above) using the cor() function in R (version 4.0.4). The resulting correlation matrix was clustered in R using the heatmaply_cor() function of the heatmaply package (Moreland, 2009). A subset comprising of variable genes was used to reduce noise generated by small, biologically meaningless fluctuations in transposon number for less variable genes.

### Homology searches

Hidden Markov model homology searches that revealed the Chorein‐N motif in Csf1 were performed using HHpred server from the MPI Bioinformatics Toolkit (Zimmermann *et al*, 2018).

### Fluorescence microscopy

Cells were grown to mid‐log phase in synthetic dextrose (SD) medium using the appropriate amino acid drop‐out mix for the selection of the plasmids. Cells were supplemented with 10 mM ethanolamine and 10 mM choline for activation of the Kennedy pathway. Images were acquired using a DeltaVision MPX microscope (Applied Precision) equipped with a 100× 1.40 NA oil UplanS‐Apo objective lens (Olympus), a multicolor illumination light source, and a CoolSNAPHQ2 camera (Roper Scientific). Image acquisition was done at RT. Images were deconvolved with SoftWoRx software using the manufacturer's parameters. Images were processed further using the Fiji ImageJ bundle. Z‐projections using MAX intensities are depicted in the figures unless mentioned otherwise.

### FM4‐64 staining

For vacuolar staining, cells were pulse labeled with FM4‐64 (Molecular Probes) at a concentration of 5 µg/ml for 20 min in the dark at 30°C. Cells were washed twice with ice‐cold media without FM4‐64 and imaged subsequently.

### Construction of “dark” GFP plasmids

“Dark” (G65T/G67A‐GFP) GFP variants were created by Quikchange mutagenesis using the original *PSD* and *PMT* plasmids as backbone.

### Thin‐layer chromatography

25 OD_600_ units of cells from a culture grown to mid‐log phase were harvested, snap‐frozen in liquid nitrogen and stored at −80°C until lipid extraction. Lipids were extracted as described previously with minor modifications (da Silveira dos Santos *et al*, 2014). Briefly, cells were washed in ice‐cold water and subsequently resuspended in 2 ml of extraction solvent containing 95% ethanol, water, diethyl ether, pyridine, and 4.2 N ammonium hydroxide (v/v 15:15:5:1:0.18). After the addition of 300 µl glass beads, samples were vortexed vigorously for 5 min and incubated at 60°C for 20 min. Cell debris were pelleted by centrifugation at 1,800 *g* for 10 min and the supernatant was dried under a stream of nitrogen. The dried extract was resuspended in 2 ml of water‐saturated butanol and sonicated for 5 min using a water bath sonicator. 1 ml of water was added and vortexed further for 2 min. After centrifugation, 250 µl of the upper butanol phase was collected, dried under a stream of nitrogen and reconstituted in 25 µl of chloroform:methanol (2:1, vol/vol) and loaded on a TLC plate (Sigma‐Aldrich Cat #1118450001). The plate was activated using 1.8% (w/v) boric acid in 100% ethanol and placed in a pre‐equilibrated TLC chamber containing the mobile phase (chloroform/ethanol/water/triethylamine (30/35/7/35, v/v)). The solvent was allowed to run till the mobile phase was ~ 2 cm before the end of the plate. After drying, lipids were visualized by spraying the plate with a primuline solution (5 mg in 100 ml acetone/water [80/20 v/v]). The plate was imaged using the ethidium bromide program on the Bio‐Rad ChemiDoc^TM^ MP Imaging System.

### Quantification of lipid signal on TLC

Images of TLC plates were quantified using the Fiji ImageJ bundle. The ImageJ gels analysis tool was used to plot the lane profile for each sample and the peak area corresponding to individual lipid species was quantified. The amount of lipid species was expressed as the fraction of the total lipid species quantified in the sample/lane. Two biological replicates were used for quantification.

### ^15^N‐serine pulse labeling

Precultures in synthetic minimal medium lacking serine were diluted to 0.05 OD_600_/ml in 25 ml and grown overnight in the presence of 10 mM ethanolamine and choline. Next day, when the cultures reached an OD_600_ of ~3, they were pelleted, washed in minimal medium without serine, ethanolamine, and choline, and diluted to 0.8 OD_600_/ml in 20 ml. To start the pulse labeling, 3 mM ^15^N‐serine (98% purity, Cambridge Isotope Laboratories) was added. At the indicated time points, 8 OD_600_ of cells were pelleted, snap‐frozen, and stored at −80°C.

### Cold stress experiment and lipidomics analysis

Cultures of wild‐type or *csf1ΔC* cells were grown until mid‐log phase at 30°C (*t* = 0). Subsequently, cultures were diluted to OD_600_ 0.3 and grown for an additional 14.5 h at 16°C. At *t* = 0 and *t* = 14.5 h, 8 OD_600_ of cells were pelleted, snap‐frozen, and stored at −80°C until further analysis. The experiment was performed using two (wt) or three (*csf1ΔC*) biological replicates.

Lipids were extracted as described for the TLC analysis and resuspended in 50% methanol. LC analysis was performed as described previously with several modifications (Castro‐Perez *et al*, 2010). Phospholipids were separated on a nanoAcquity UPLC (Waters) equipped with a HSS T3 capillary column (150 m × 30 mm, 1.8 m particle size, Waters), applying a 10‐min linear gradient of buffer A (5 mM ammonium acetate in acetonitrile/water 60:40) and B (5 mM ammonium acetate in isopropanol/acetonitrile 90:10) from 10% B to 100% B. Conditions were kept at 100% B for the next 7 min, followed by a 8 min re‐equilibration to 10% B. The injection volume was 1 µl. The flow rate was constant at 2.5 µl/min.

The UPLC was coupled to QExactive mass spectrometer (Thermo) by a nanoESI source (New Objective Digital PicoView^®^ 550). The source was operated with a spray voltage of 2.9 kV in positive mode and 2.5 kV in negative mode. Sheath gas flow rate was set to 25 and 20 for positive and negative mode, respectively. MS data were acquired using either positive or negative polarization, alternating between full MS and all ion fragmentation (AIF) scans. Full‐scan MS spectra were acquired in profile mode from 107–1,600 *m*/*z* with an automatic gain control target of 1e6, an Orbitrap resolution of 70,000, and a maximum injection time of 200 ms. AIF spectra were acquired from 107–1,600 *m*/*z* with an automatic gain control value of 5e4, a resolution of 17,500, a maximum injection time of 50 ms and fragmented with a normalized collision energy of 20, 30 and 40 (arbitrary units). Generated fragment ions were scanned in the linear trap. Positive‐ion‐mode was employed for monitoring PC and negative‐ion‐mode was used for monitoring PS and PE. Lipid species were identified based on their *m*/*z* and elution time. We used a standard mixture comprising PS 10:0/10:0, PE 17:0/17:0, and PC 17:0/17:0 for deriving an estimate of specific elution times. Lipid intensities were quantified using the Skyline software (Adams *et al*, 2020).

### Statistical analysis

In Fig 2B, the standard error of the mean (SEM) is computed as the standard deviation divided by the square root of the number of replicates. To generate volcano plots (Figs 3C, 4B, 5A and EV3), two sets of libraries were defined: a test set and a reference set. For both sets, the mean of normalized transposon counts or read counts per gene were computed. The fold change for each gene was calculated as the mean of the test set divided by that of the reference set and a *P*‐value associated with this difference was computed using a Student's *t*‐test. In figure D, *P*‐values are computed using an unpaired two‐sided Student's *t*‐test.

## Author contributions

**Arun T John Peter:** Conceptualization; Data curation; Formal analysis; Funding acquisition; Validation; Investigation; Visualization; Methodology; Writing—original draft; Writing—review and editing. **Sabine N van Schie:** Conceptualization; Data curation; Formal analysis; Investigation; Visualization; Methodology; Writing—original draft; Writing—review and editing. **Ngaam J Cheung:** Software; Visualization. **Agnes H Michel:** Resources; Investigation. **Matthias Peter:** Supervision; Funding acquisition; Project administration. **Benoit Kornmann:** Conceptualization; Data curation; Formal analysis; Supervision; Funding acquisition; Visualization; Writing—original draft; Project administration; Writing—review and editing.

In addition to the CRediT author contributions listed above, the contributions in detail are:

ATJP, SNSvS, and BK conceived the study. ATJP and SNSvS designed and performed the experiments under the supervision of MP and BK. ATJP, SNSvS, and BK analyzed data. SNSvS and NJC performed bioinformatics analyses. NJC carried out work on making the SATAY data available on a web interface. AHM designed and constructed the plasmid for the SATAY screens. SNSvS, ATJP, and BK wrote the paper with input from all the authors.

## Supporting information



Appendix

Expanded View Figures PDF

Source Data for Expanded View/Appendix

Source Data for Figure 3

Source Data for Figure 5

Source Data for Figure 7

## Data Availability

Sequencing data are deposited in the European Nucleotide Archive (ENA) under accession number PRJEB48777 (http://www.ebi.ac.uk/ena/data/view/PRJEB48777). Data analyses are available in the supplementary data, at http://genome‐euro.ucsc.edu/s/benjou/rewiring_paper and https://kornmann.bioch.ox.ac.uk/satay/rewiring. Lipidomics data related to this study are provided in SData Fig EV5 and SData Fig 7.
