# Supplementary material for: Rewiring phospholipid biosynthesis reveals resilience to membrane perturbations and uncovers regulators of lipid homeostasis
Source: EMBO J. 2022 Feb 21;41(7):e109998. doi: 10.15252/embj.2021109998 (PMC8982615; doi:10.15252/embj.2021109998)
Supplement: Supplementary file 1 — Appendix [file EMBJ-41-e109998-s007.pdf]

Appendix

Rewiring Phospholipid Biosynthesis Reveals Resilience to Membrane Perturbations and Uncovers Regulators of Lipid Homeostasis

Table of contents

Appendix Methods

Appendix Figure S1 Co-localization of the Pmt mitochondrial constructs with a mitochondrial marker

Appendix Figure S2 Co-localization of chimeric constructs with a LD and peroxisome marker

Appendix Figure S3 Co-localization of FYVE-domain containing constructs with the endocytic compartment dye FM4-64

Appendix Figure S4 Localization of Pmt (MM) in different rewired strains

Appendix Figure S5 Localization of Pmt (ER) in different rewired strains

Appendix Figure S6 Localization of Pmt (peroxisome) in different rewired strains

Appendix Figure S7 Localization of Pmt (endosome) in different rewired strains

Appendix Figure S8 Localization of Pmt (LD) in different rewired strains

Appendix Figure S9 Localization of Pmt (MIM) in different rewired strains

Appendix Figure S10 Localization of Psd (MIM) in different rewired strains

Appendix Figure S11 Localization of Psd (LD) in different rewired strains

Appendix Figure S12 Localization of Psd (endosome) in different rewired strains

Appendix Figure S13 Localization of Psd (ER) in different rewired strains

Appendix Figure S14 Hierarchical clustering of gene transposon insertions profiles for all libraries

Appendix Figure S15 Vps13 is required in libraries where lipid synthesis occurs at its native locations

Appendix Figure S16 *CSF1* is not required when PE is targeted to endosomes and PC is targeted to LD.

Appendix Figure S17 Oleic acid rescues the cold sensitive phenotype of *csf1ΔC* mutants.

Appendix Table S1 Yeast strain used in this study

Appendix Table S2 Plasmids used in this study

Appendix Table S3 Primers used in this study

References

Appendix Methods

Yeast media composition

10x Amino acid mix: L-Isoleucine (Sigma-Aldrich I2752) 0.3 g/L, L-Valine (Roth, 4879.3) 3 g/L, Adenine Hemisulfate salt (Sigma-Aldrich, A9126) 0.4 g/L, L-Arginine monohydrochloride (Sigma-Aldrich, A5131) 0.2 g/L, L-Histidine monohydrochloride monohydrate (Sigma-Aldrich, H8251) 0.2 g/L, L-Leucine (Sigma-Aldrich, L8000) 1 g/L, L-Lysine monohydrochloride (Sigma-Aldrich, L5626) 0.3 g/L, L-Methionine (Sigma-Aldrich, M9625) 0.2 g/L, L-Phenylalanine(Sigma-Aldrich, P2126) 0.5 g/L, L-Threonine (Sigma-Aldrich, T8625) 2 g/L, L-Tryptophan (Sigma-Aldrich, T0254) 0.4 g/L, L-Tyrosine (Sigma-Aldrich, T3754) 0.3 g/L, Uracil (Sigma-Aldrich, U0750) 0.2 g/L, L-Glutamic Acid monosodium salt hydrate (Sigma-Aldrich, G1626) 1 g/L, L-Aspartic Acid sodium salt monohydrate (Sigma-Aldrich, 11195) 1 g/L. Any amino acid can be dropped out at will.

4x S: 6.8 g/L Yeast Nitrogen base without Amino Acid and Ammonium Sulfate (Difco, 233520), 20 g/L Ammonium Sulfate (Sigma-Aldrich, A4418).

SC medium: 1x amino acid mix, 1x S, carbon source (glucose, galactose) 2%.

YPD medium: 20 g BactoPeptone (Difco), 10 g Yeast Extract (Difco).
For solid media, 2% Agar (Difco, 214530) is added.

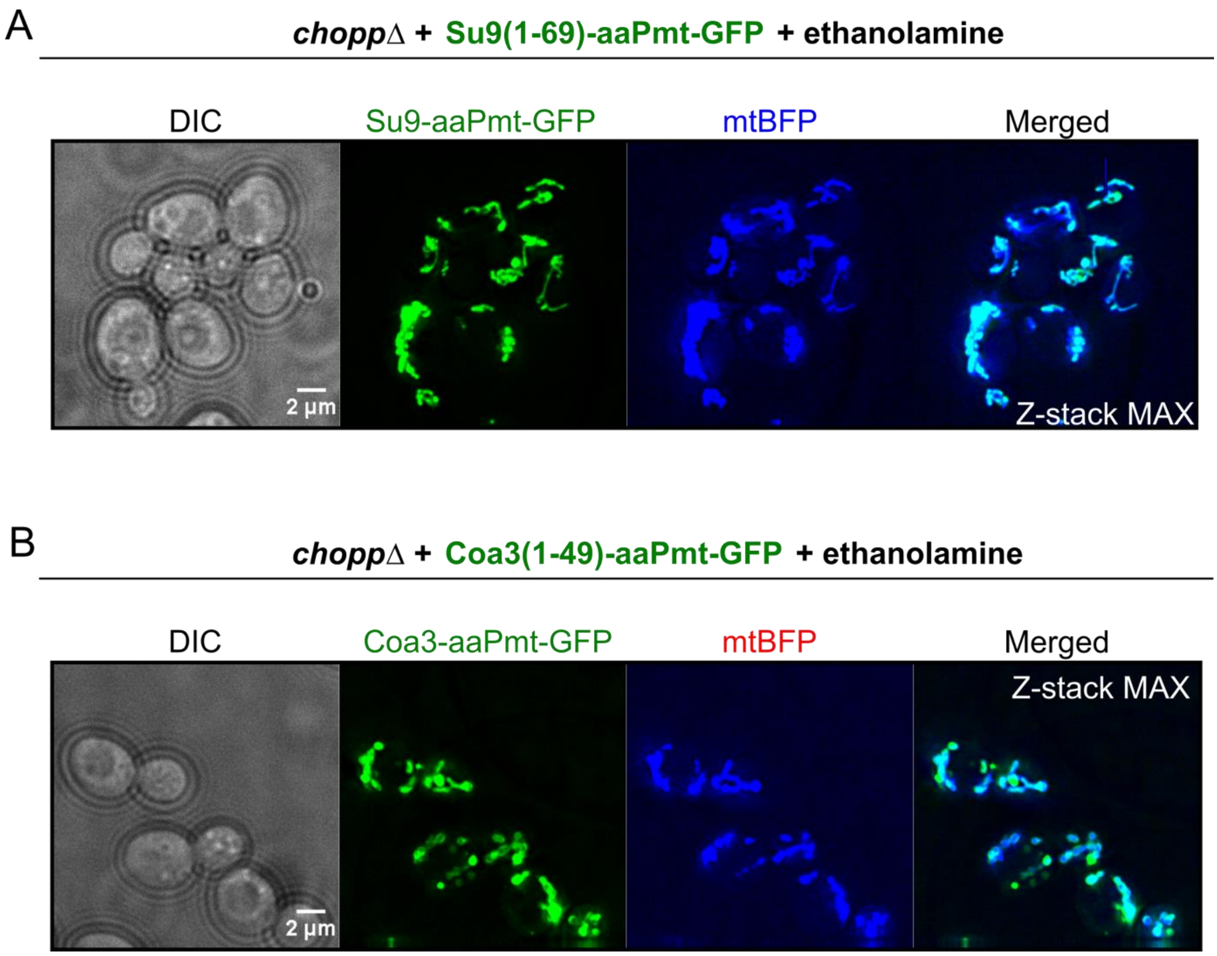

Appendix Figure S1. Co-localization of the Pmt mitochondrial constructs with a mitochondrial marker.

A) Co-localization of Su9-aaPmt-GFP (MM) with mtBFP (MM marker) expressed in *choppΔ* cells, grown in SD medium supplemented with 10 mM ethanolamine. Images shown are maximum intensity projections of several Z-sections.

B) Co-localization of Coa3-aaPmt-GFP (MIM) with mtBFP (MM marker) expressed in *choppΔ* cells, grown in SD medium supplemented with 10 mM ethanolamine. Images shown are maximum intensity projections of several Z-sections.

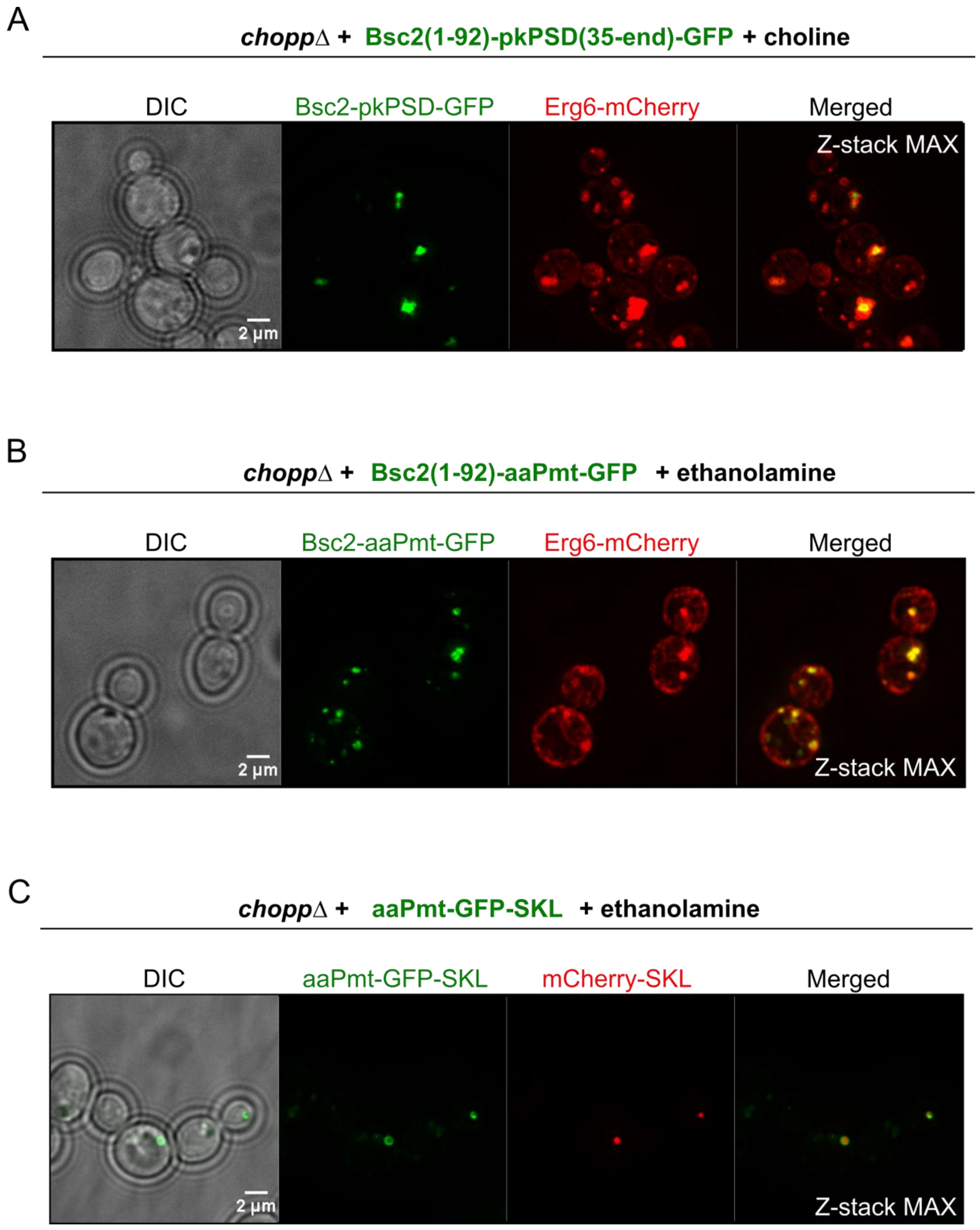

Appendix Figure S2. Co-localization of chimeric constructs with a LD and peroxisome marker.

A) Co-localization of Bsc2-pkPsd-GFP with Erg6-mCherry (LD marker) expressed in *choppΔ* cells, grown in SD medium supplemented with 10 mM choline. Images shown are maximum intensity projections of several Z-sections.

B) Co-localization of Bsc2-aaPmt-GFP with Erg6-mCherry (LD marker) expressed in *choppΔ* cells, grown in SD medium supplemented with 10 mM ethanolamine. Images shown are maximum intensity projections of several Z-sections.

C) Co-localization of aaPmt-GFP-SKL with mCherry-SKL (peroxisome marker) expressed in *choppΔ* cells, grown in SD medium supplemented with 10 mM ethanolamine. Images shown are maximum intensity projections of several Z-sections.

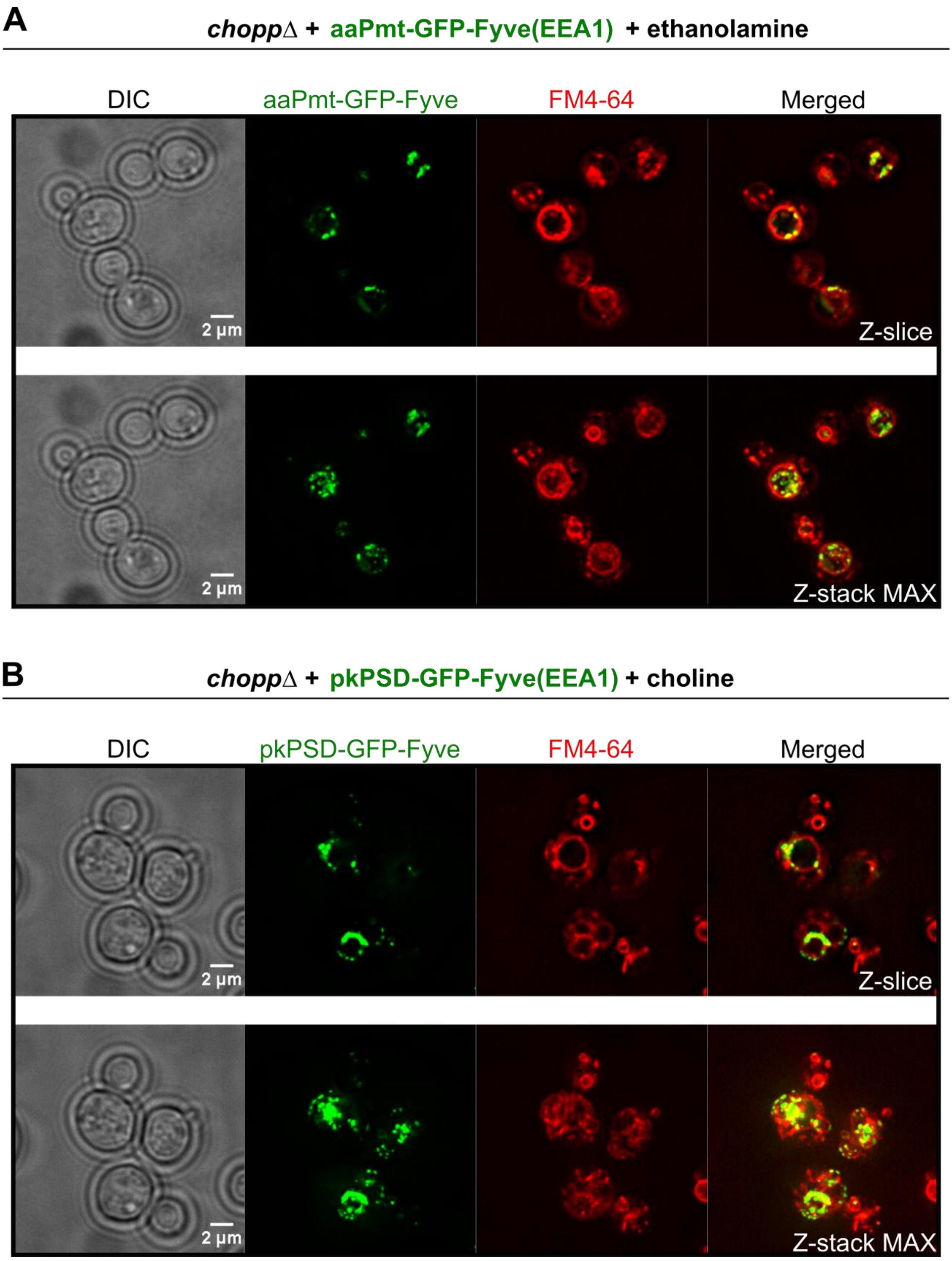

Appendix Figure S3. Co-localization of FYVE-domain containing constructs with the endocytic compartment dye FM4-64.

A) Co-localization of aaPmt -GFP-Fyve expressed in *choppΔ* cells with FM4-64, grown in SD medium supplemented with 10 mM ethanolamine. Images shown are either a single z-slice or maximum intensity projections of several Z-sections, as indicated.

B) Co-localization of pkPsd -GFP-Fyve expressed in *choppΔ* cells with FM4-64, grown in SD medium supplemented with 10 mM choline. Images shown are either a single z-slice or maximum intensity projections of several Z-sections, as indicated.

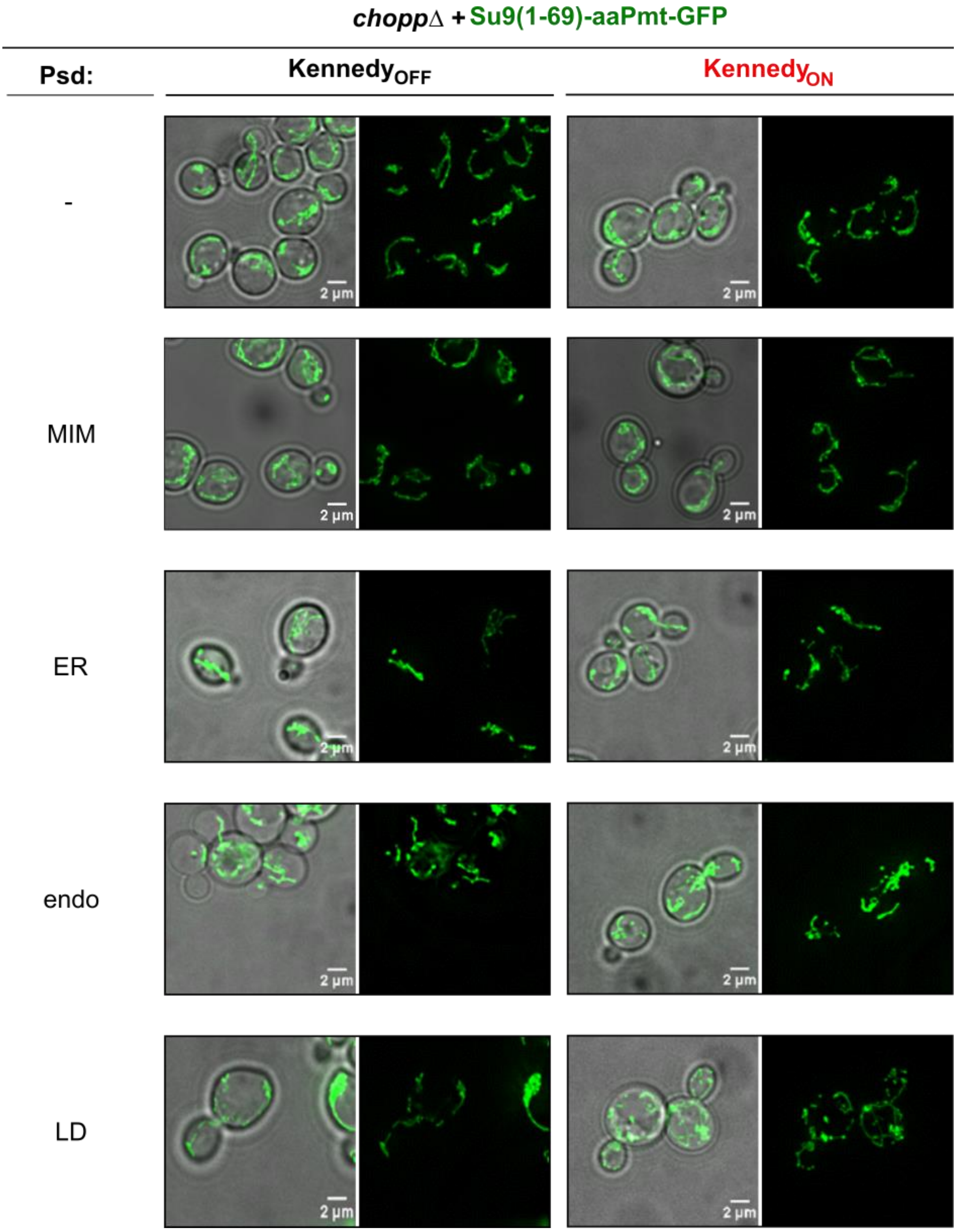

Appendix Figure S4. Localization of Pmt (MM) in different rewired strains.

Localization of the Su9(ss)-aaPmt-GFP (Pmt-MM) construct expressed in *choppΔ* cells with PE produced either by the Kennedy pathway (+10 mM ethanolamine) or a 'dark' version of one of the Psd constructs, as indicated. Images shown are maximum intensity projections of several Z-sections.

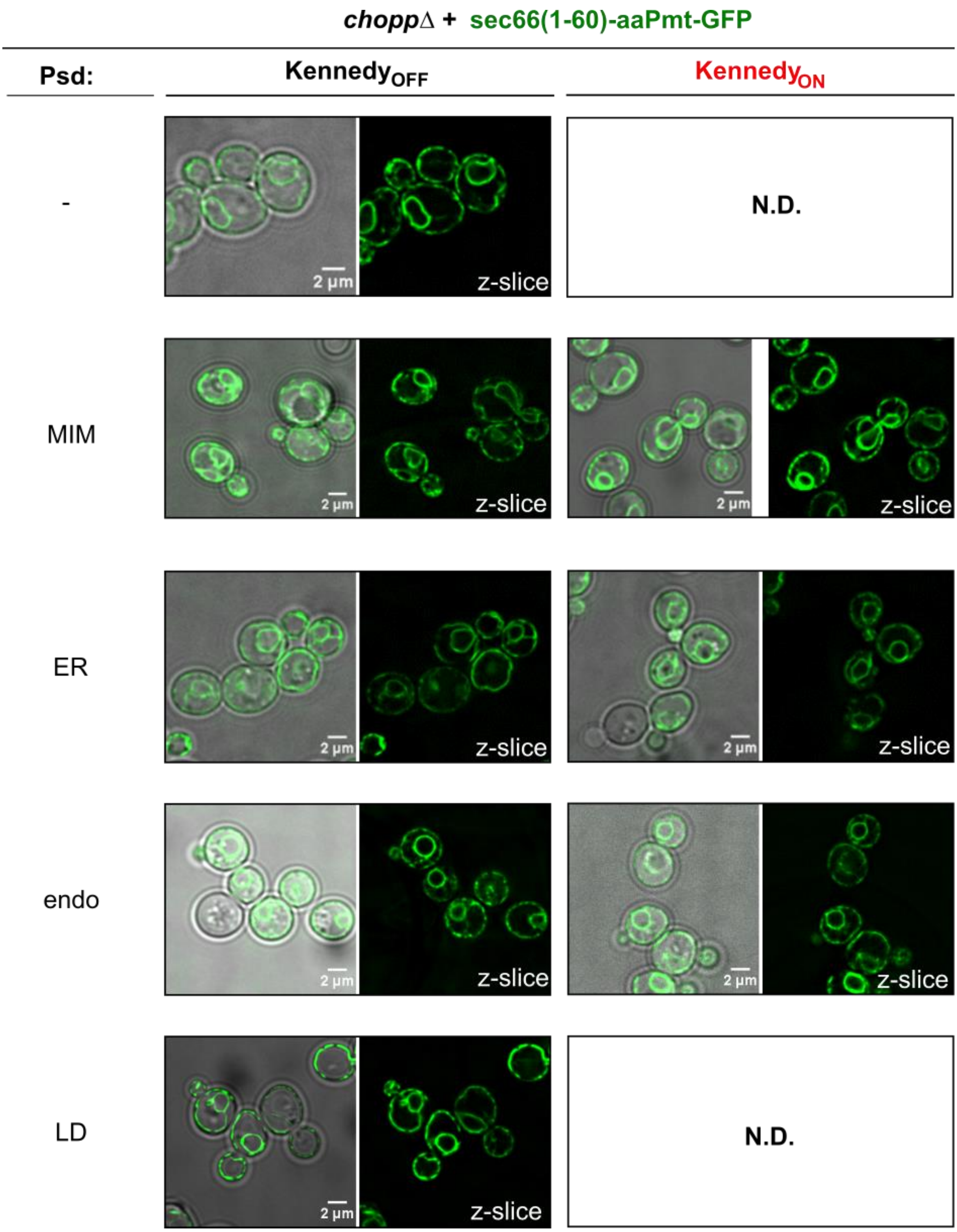

Appendix Figure S5. Localization of Pmt (ER) in different rewired strains.

Localization of sec66(1-60)-aaPmt-GFP (Pmt-ER) construct expressed in *choppΔ* cells with PE produced either by the Kennedy pathway (+10 mM ethanolamine) or a 'dark' version of one of the Psd constructs, as indicated. Images shown represent one Z-section.

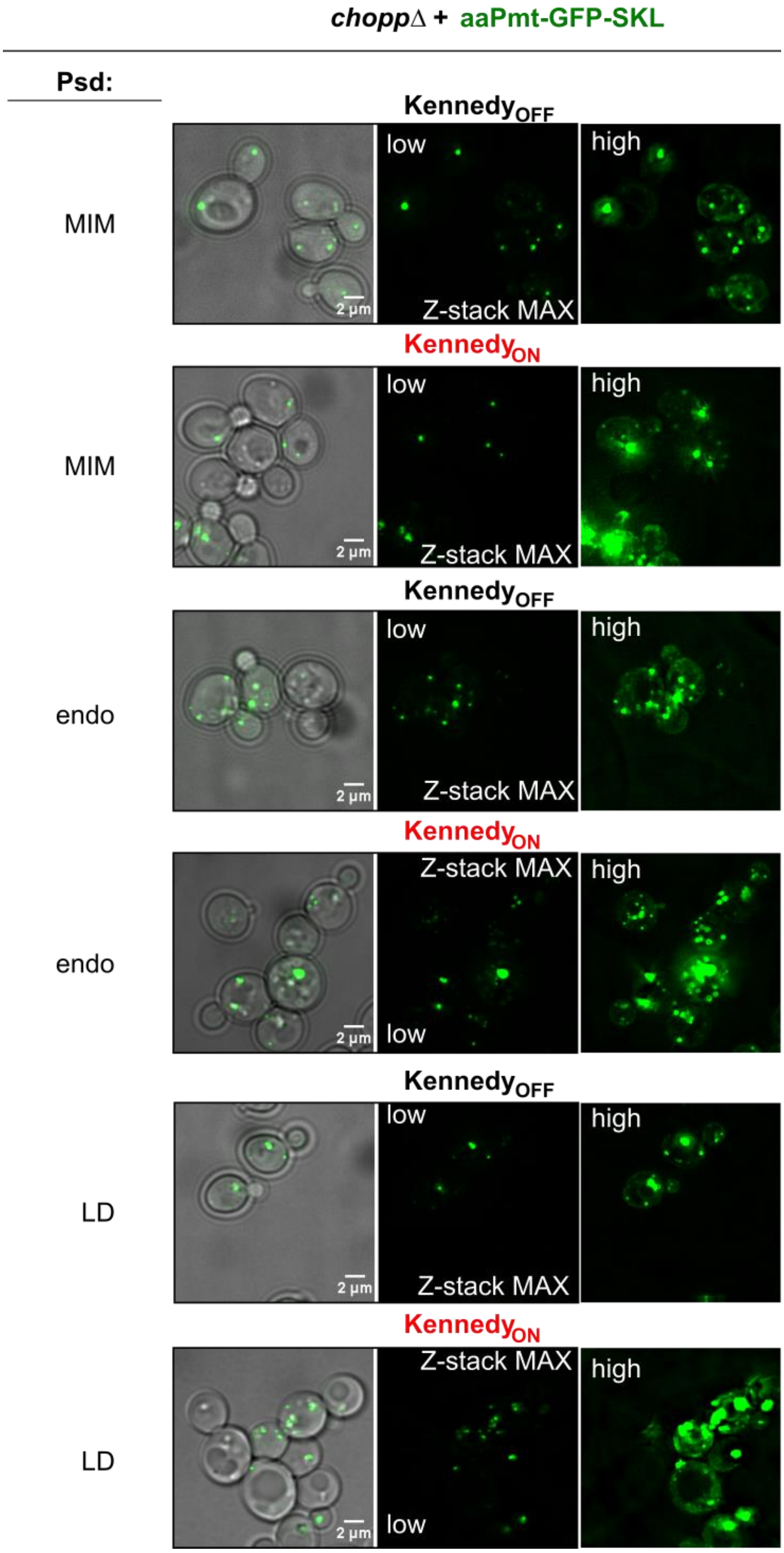

Appendix Figure S6. Localization of Pmt (peroxisome) in different rewired strains.

Localization of the aaPmt-GFP-SKL (Pmt-pex) construct expressed in *choppΔ* cells together with a 'dark' version of one of the Psd constructs, as indicated. Images shown are maximum intensity projections of several Z-sections.

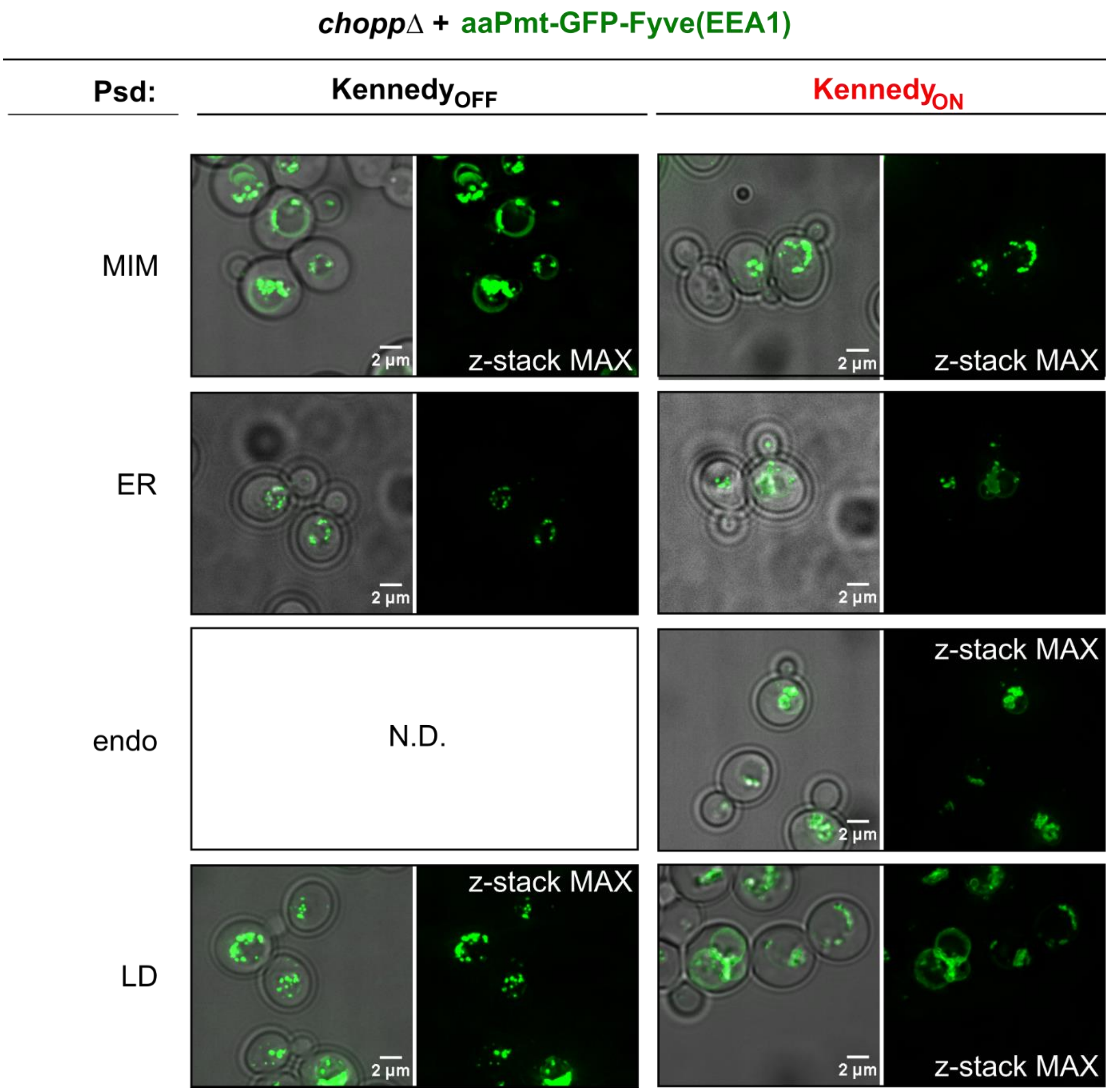

Appendix Figure S7. Localization of Pmt (endosome) in different rewired strains.

Localization of the aaPmt-GFP-Fyve (Pmt-endo) construct expressed in *choppΔ* cells together with a 'dark' version of one of the Psd constructs, as indicated. Images shown are maximum intensity projections of several Z-sections.

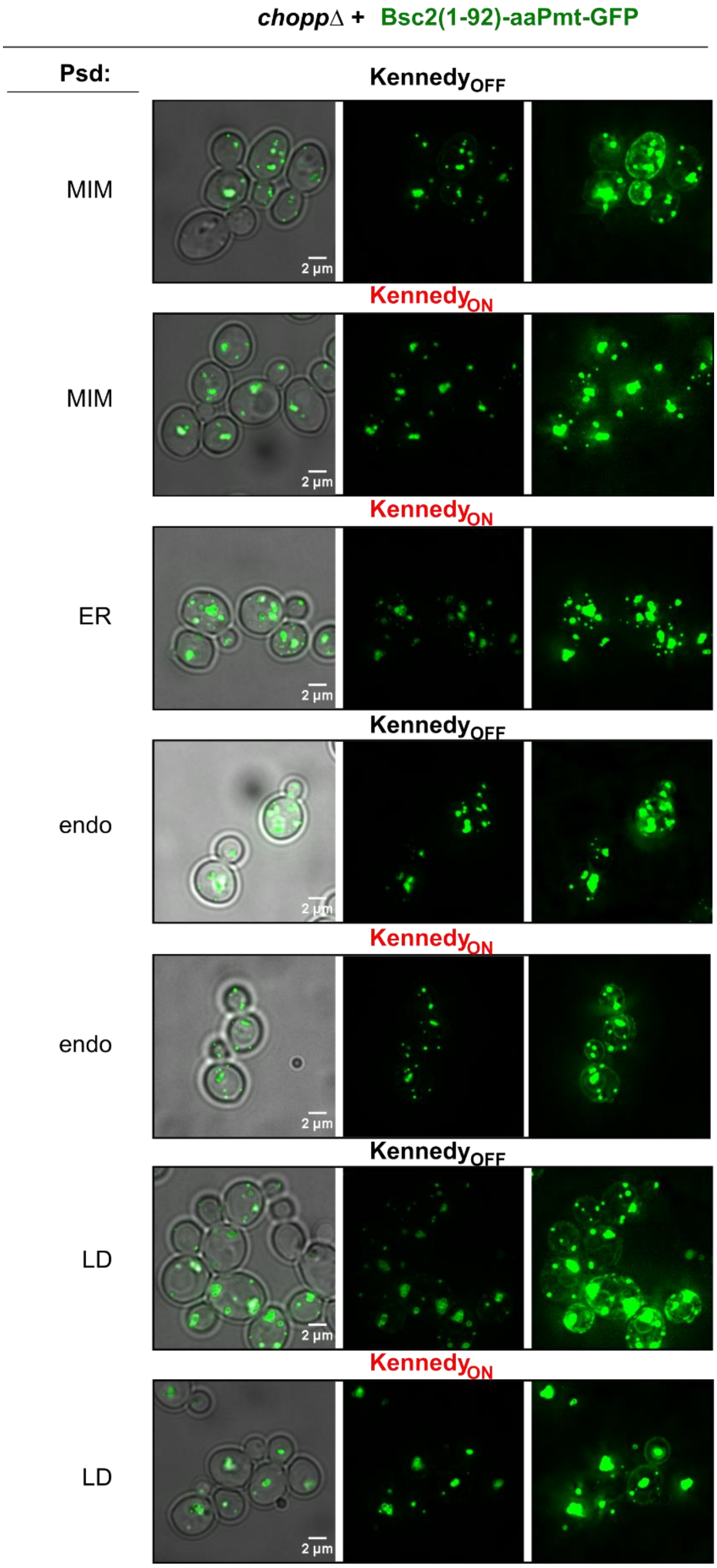

Appendix Figure S8. Localization of Pmt (LD) in different rewired strains.

Localization of the Bsc2(tm)-aaPmt-GFP (Pmt-LD) construct expressed in *choppΔ* cells together with a 'dark' version of one of the Psd constructs, as indicated. Images shown are maximum intensity projections of several Z-sections.

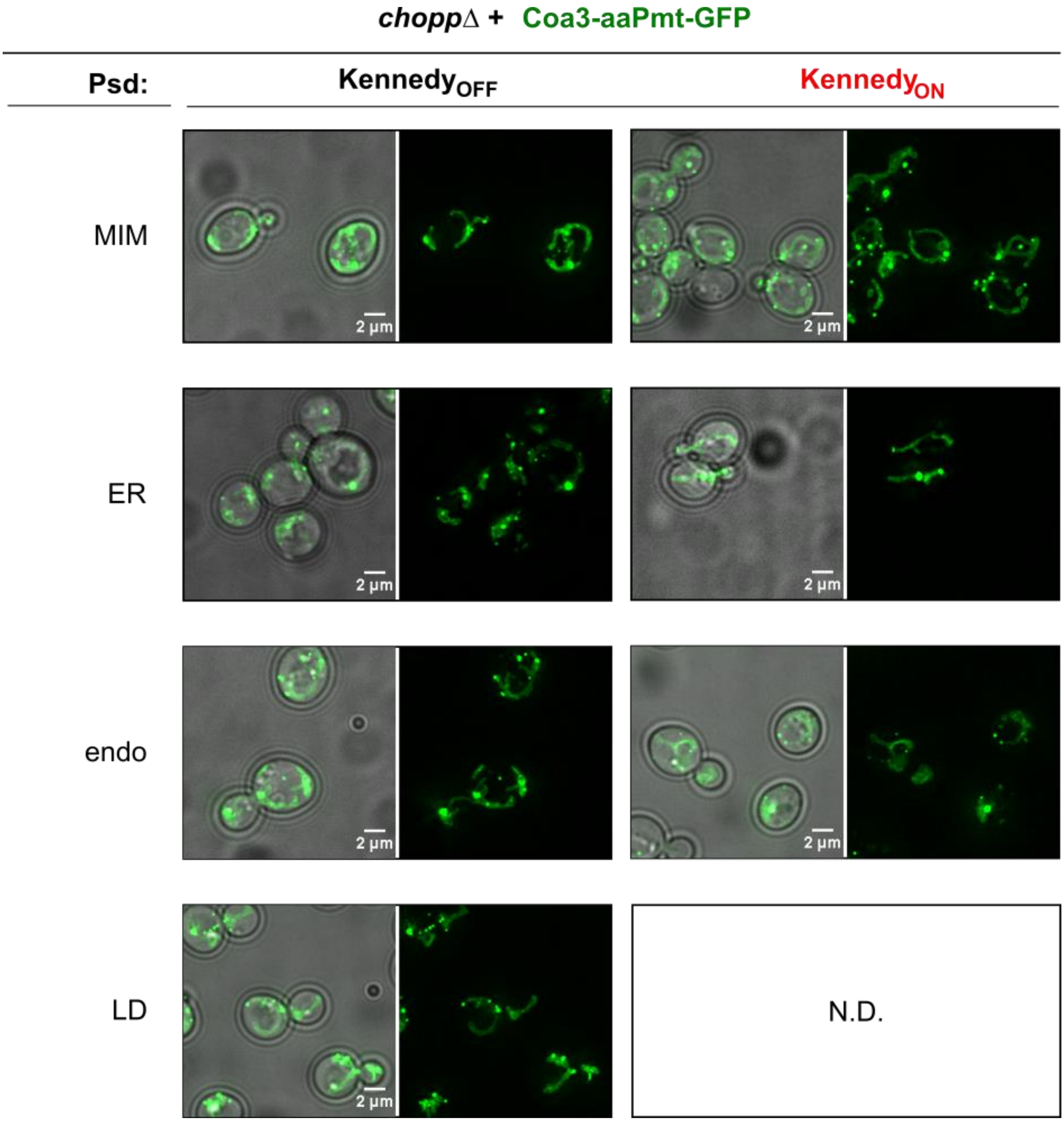

Appendix Figure S9. Localization of Pmt (MIM) in different rewired strains.

Localization of the Coa3(tm)-aaPmt-GFP (Pmt-MIM) construct expressed in *choppΔ* cells together with a 'dark' version of one of the Psd constructs, as indicated. Images shown are maximum intensity projections of several Z-sections.

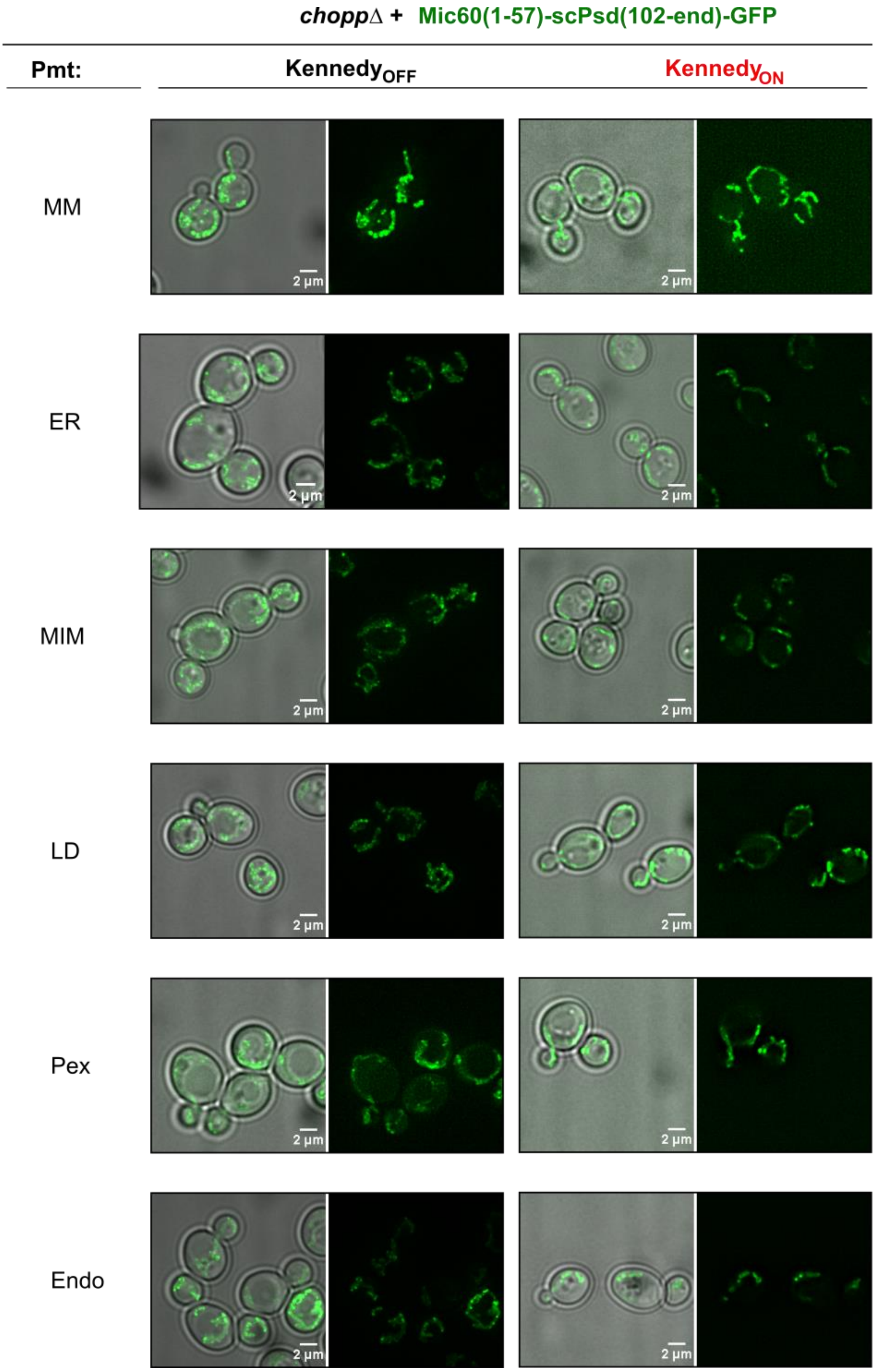

Appendix Figure S10. Localization of Psd (MIM) in different rewired strains.

Localization of the Mic60(tm)-scPsd-GFP (Psd-MIM) construct expressed in *choppΔ* cells together with a 'dark' version of one of the Pmt constructs, as indicated. Images shown are maximum intensity projections of several Z-sections.

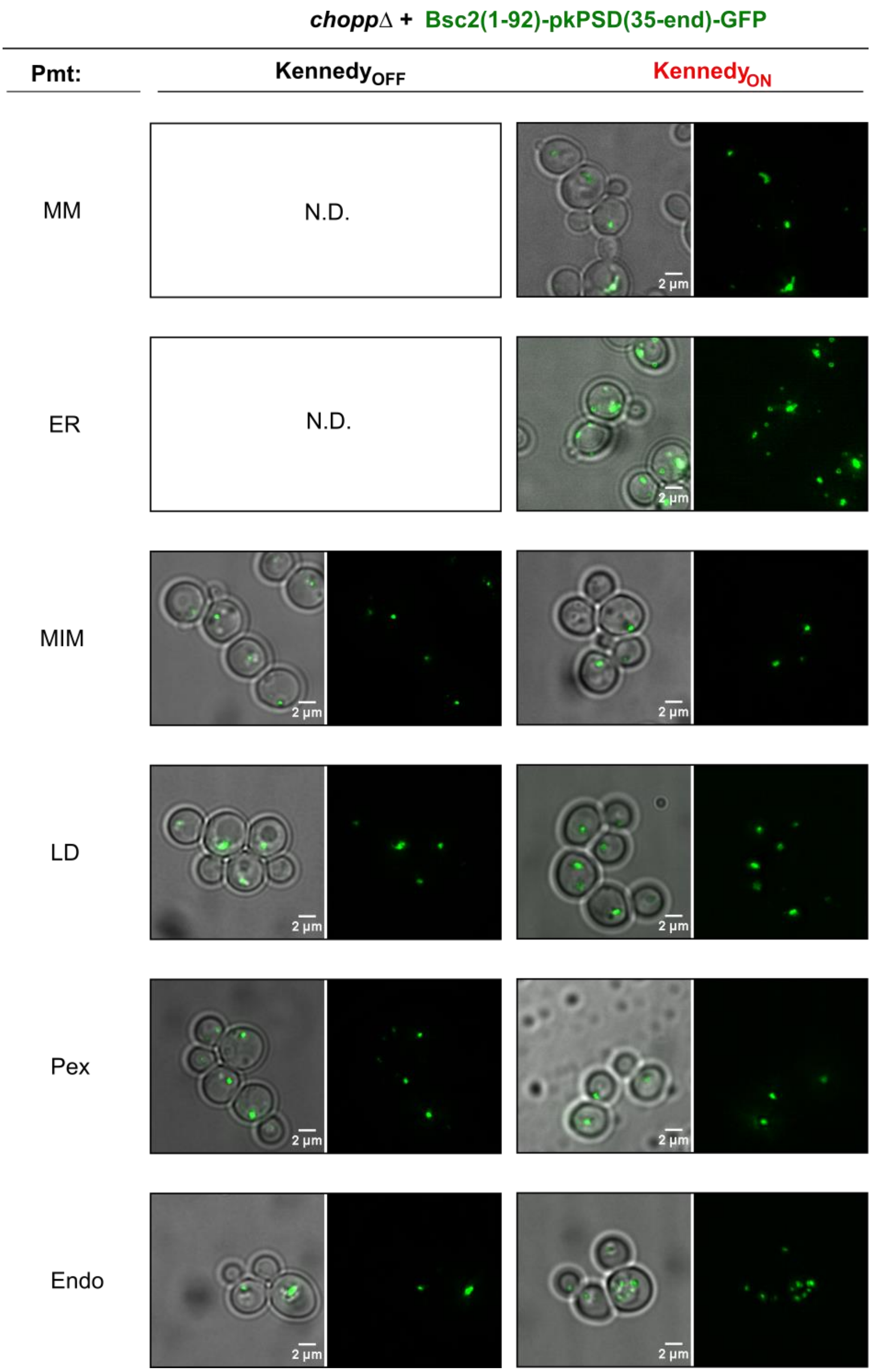

Appendix Figure S11. Localization of Psd (LD) in different rewired strains.

Localization of the Bsc2(tm)-pkPsd-GFP (Psd-LD) construct expressed in *choppΔ* cells together with a 'dark' version of one of the Pmt constructs, as indicated. Images shown are maximum intensity projections of several Z-sections.

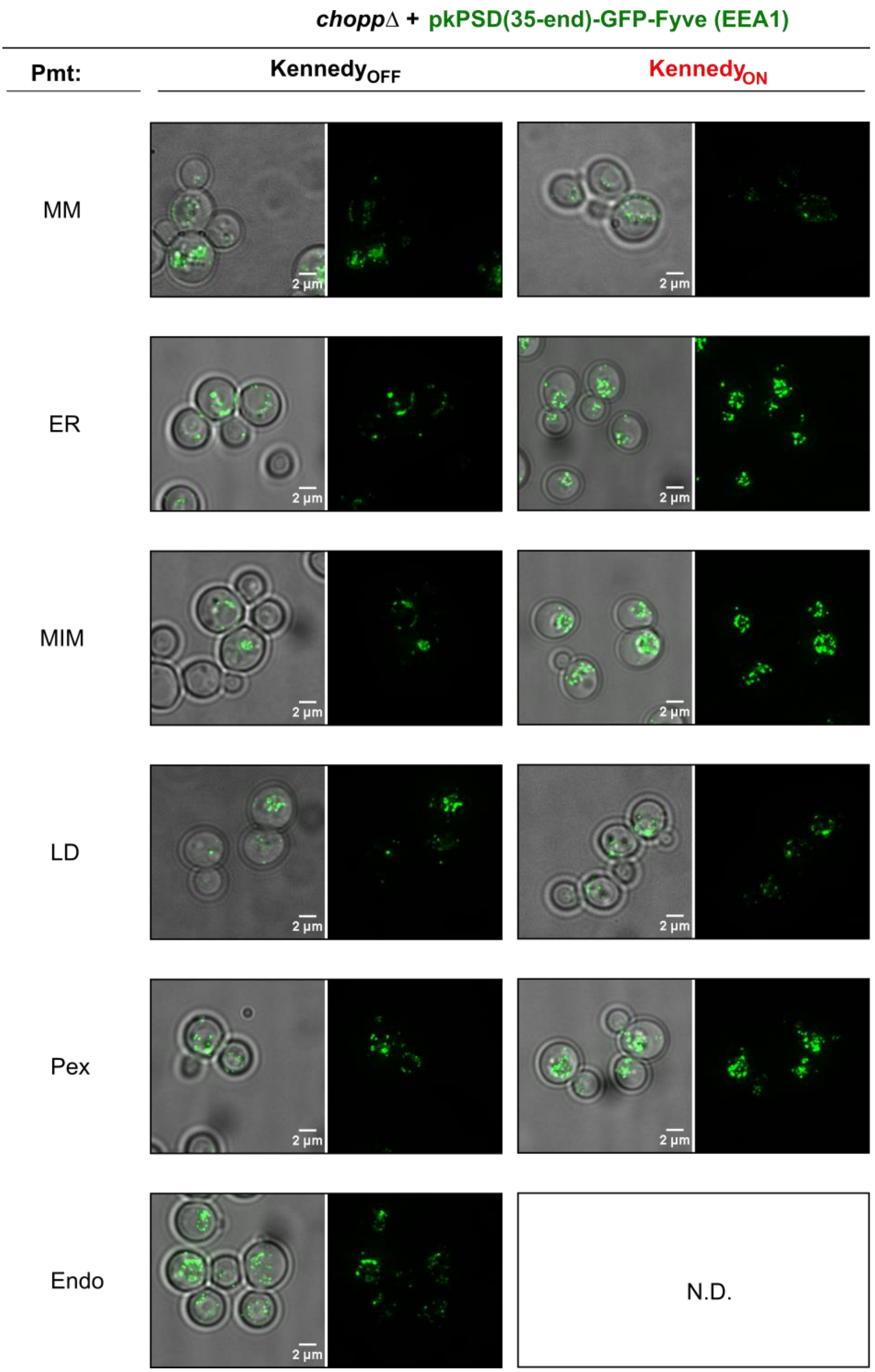

Appendix Figure S12. Localization of Psd (endosome) in different rewired strains.

Localization of the pkPsd-GFP-Fyve (Psd-endo) construct expressed in *choppΔ* cells together with a 'dark' version of one of the Pmt constructs, as indicated. Images shown are maximum intensity projections of several Z-sections.

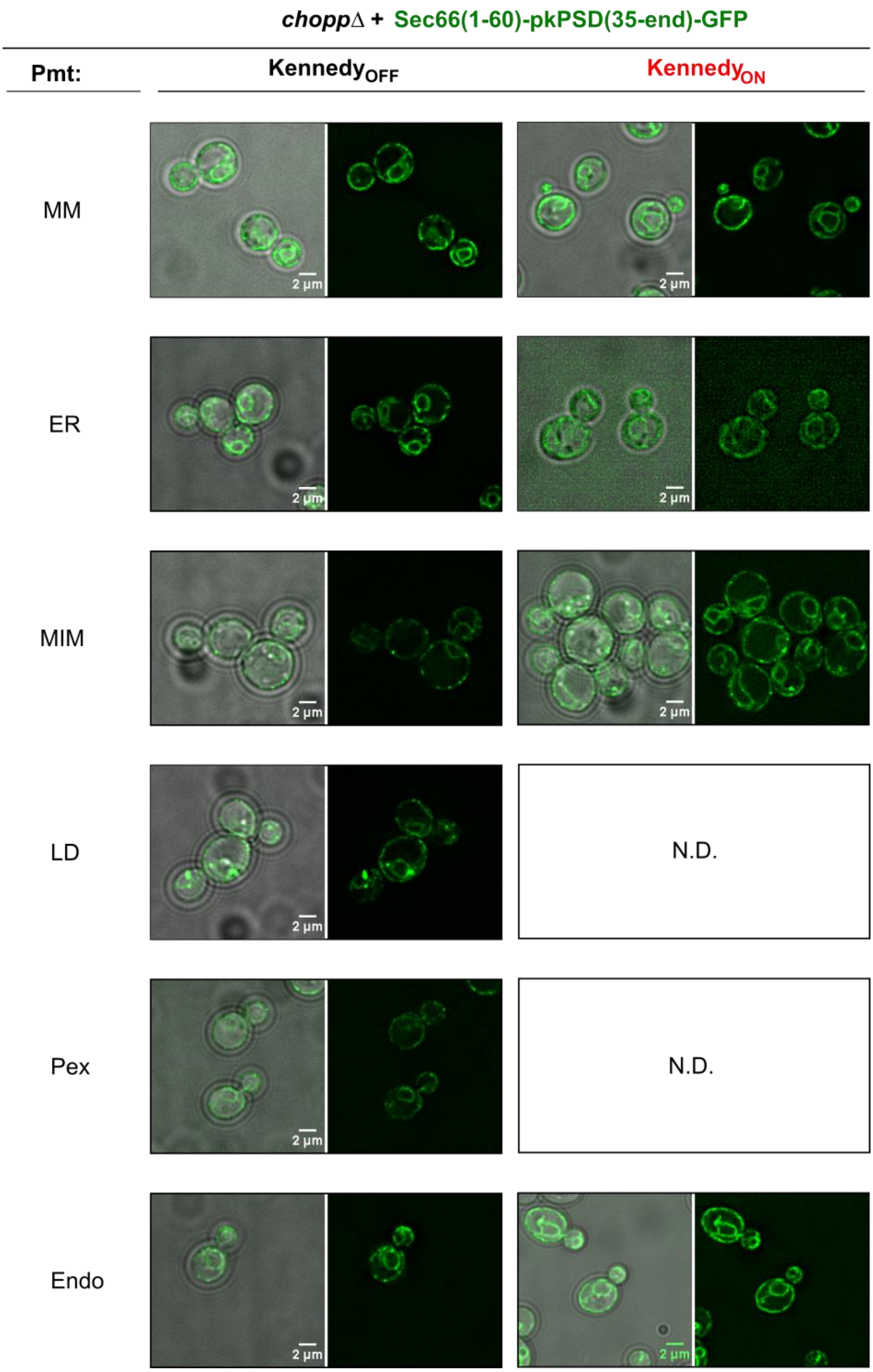

Appendix Figure S13. Localization of Psd (ER) in different rewired strains.

Localization of the Sec66(tm)-pkPsd-GFP (Psd-ER) construct expressed in *choppΔ* cells together with a 'dark' version of one of the Pmt constructs, as indicated. Images shown are maximum intensity projections of several Z-sections.

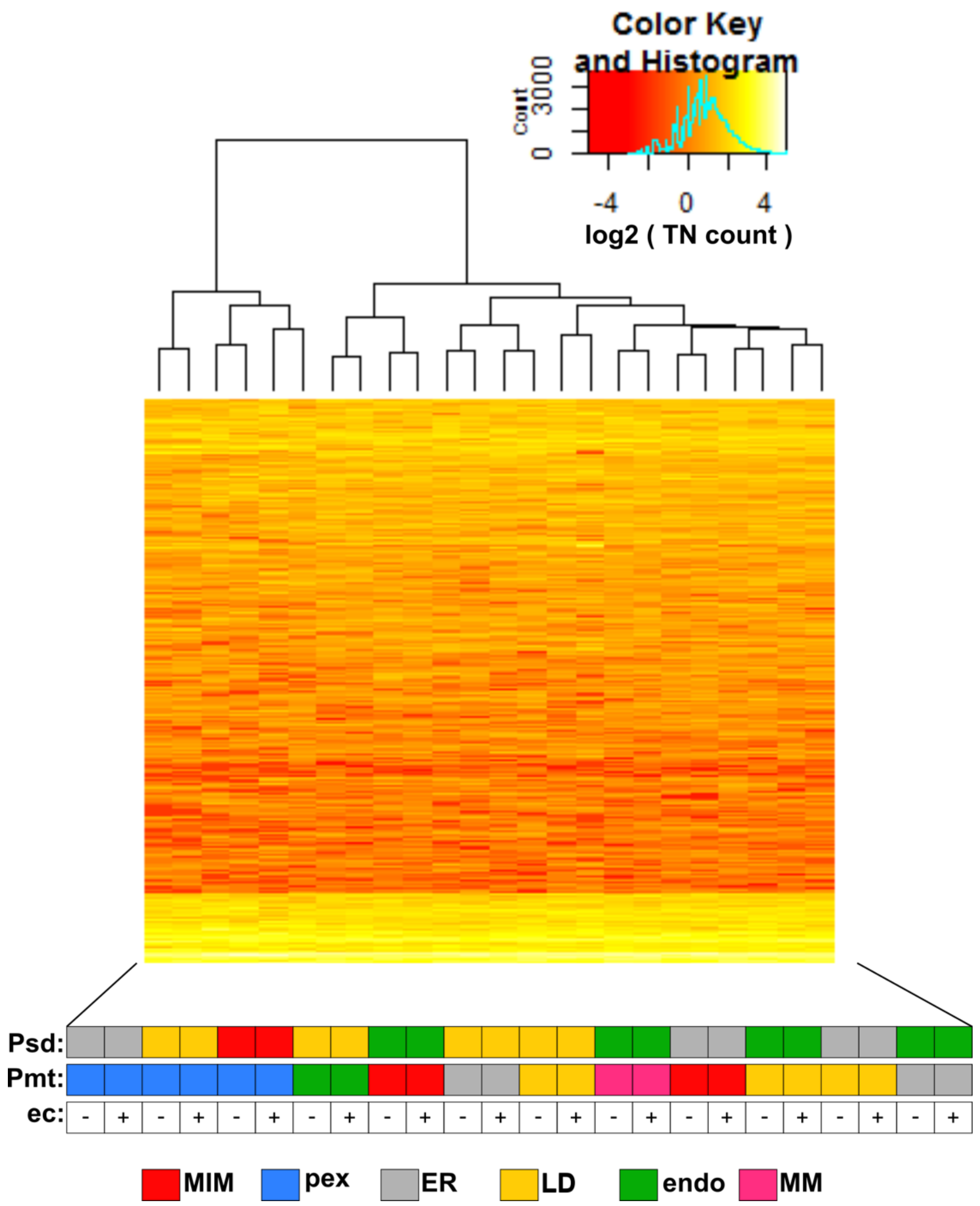

Appendix Figure S14. Hierarchical clustering of gene transposon insertions profiles for all libraries.

Hierarchical clustering of transposon numbers per gene (rows) computed for all libraries (columns). Color key depicts the log2 fold change of transposon count (TN) for each gene in a library with respect to the mean TN count per gene of all libraries. Dendrogram for library clustering is shown on top. The bottom panel depicts the localization of the Psd (PE) and Pmt (PC) enzymes and the growth conditions for each library. Libraries grown in Kennedy$_{OFF}$ and $_{ON}$ conditions are indicated with - and +, respectively.

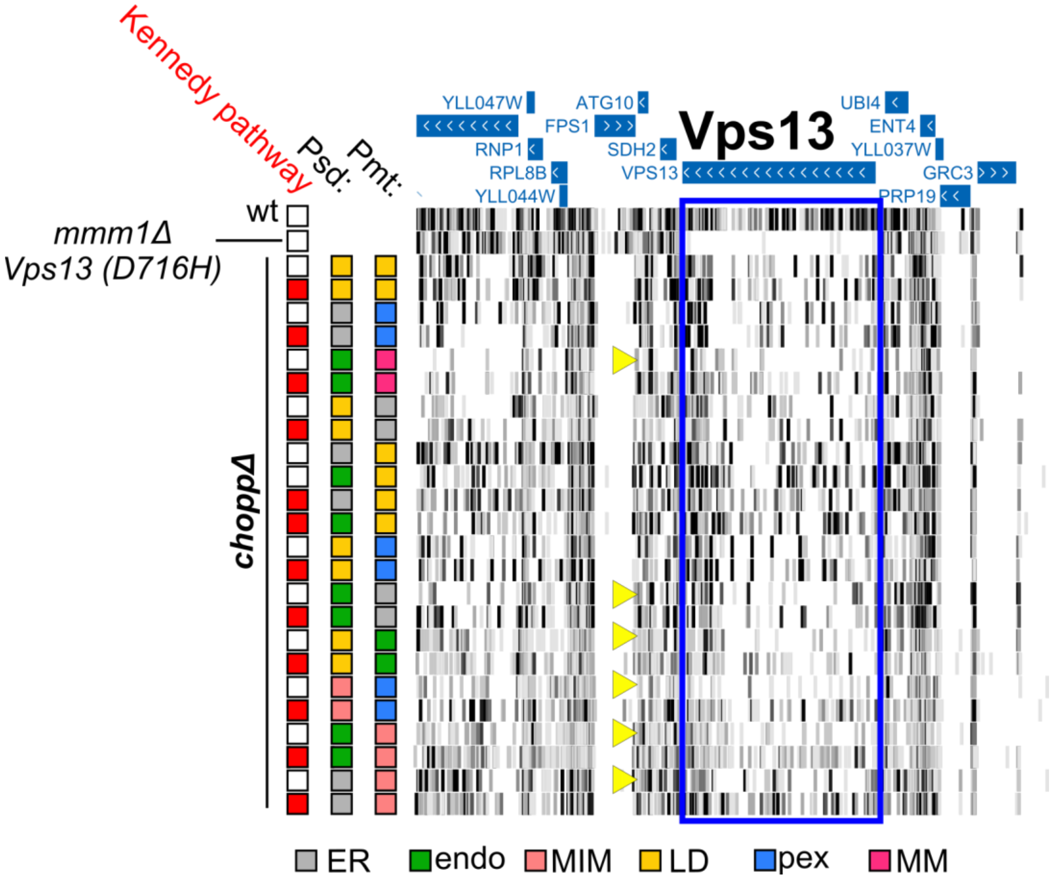

Appendix Figure S15. Vps13 is required in libraries where lipid synthesis occurs at its native locations.

Transposon insertion maps generated in the UCSC genome browser for the *VPS13* genomic locus for the indicated genotypes. Source of PE and PC corresponds to the Psd and Pmt constructs expressed in the *choppΔ* strain and targeted to the indicated locations. Red box = Kennedy$_{ON}$, white box = Kennedy$_{OFF}$. In addition, a library is displayed where Vps13 is not required (wt) and a library where Vps13 is expected to be essential (*mmm1Δ* (ERMESΔ) v*ps13D176H* (ERMES suppressor allele)). Yellow arrowheads highlight rewired libraries grown in Kennedy$_{OFF}$ conditions where the number of transposons is depleted with respect to the same library grown in Kennedy$_{ON}$ conditions.

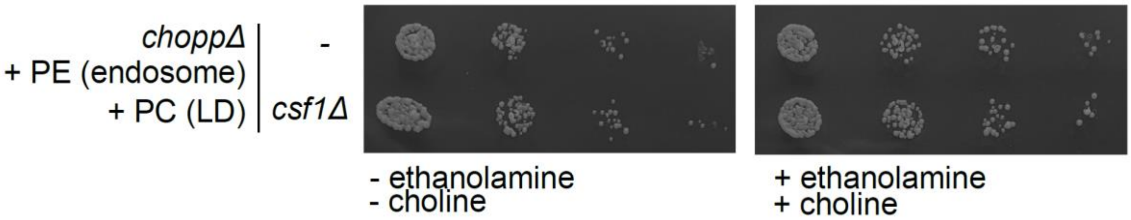

Appendix Figure S16. *CSF1* is not required when PE is targeted to endosomes and PC is targeted to LD.

Five-fold serial dilutions of strains of the indicated genotypes on SD medium without ethanolamine and choline (Kennedy$_{OFF}$), or with ethanolamine and choline (Kennedy$_{ON}$).

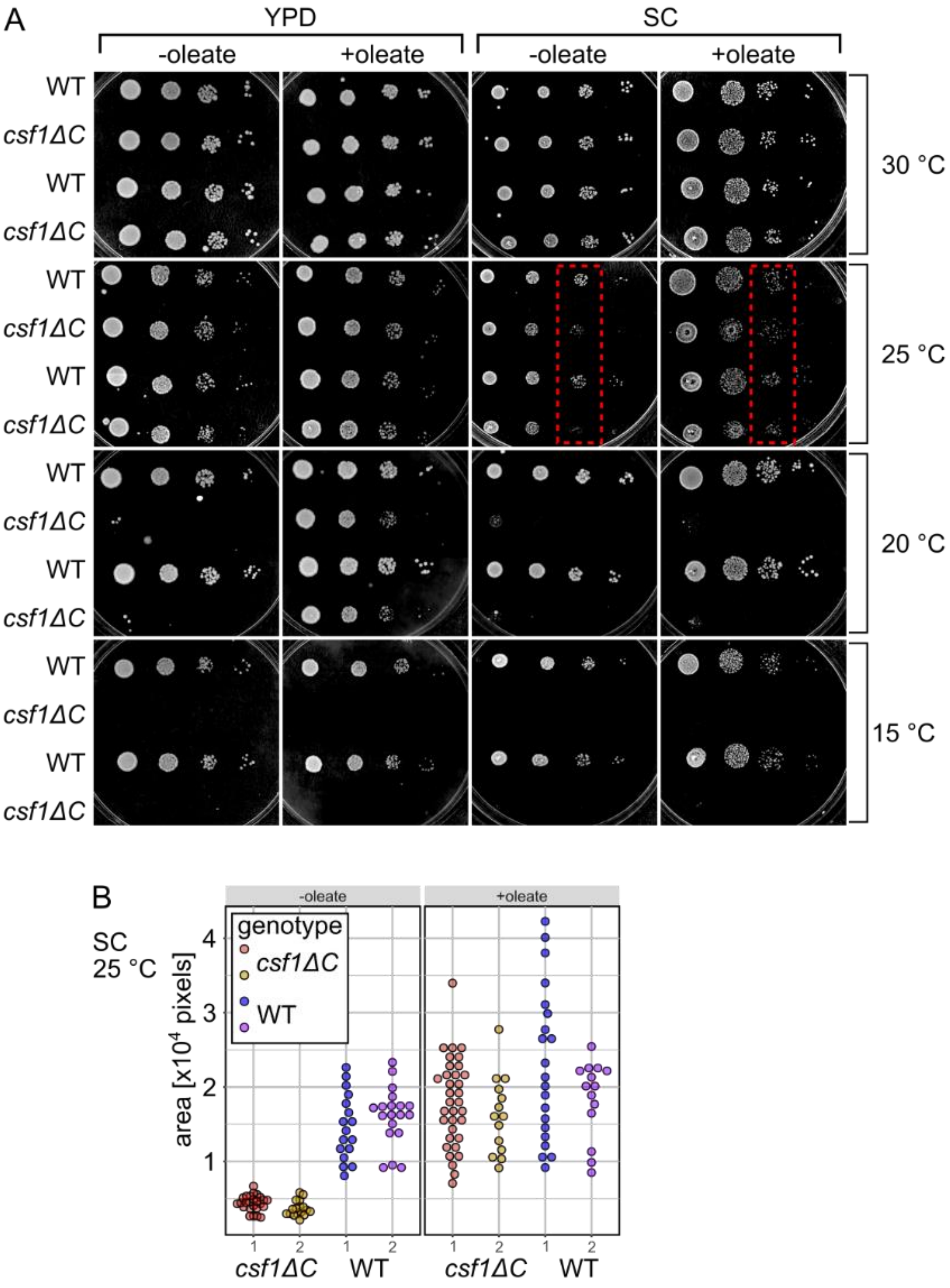

Appendix Figure S17. Oleic acid rescues the cold sensitive phenotype of *csf1ΔC mutants*.

A) As in Figure 7F with expanded range of temperatures and in two different media, complex medium YPD (left) and synthetic medium SC (right). Red boxes are areas quantified in B).

B) Oleic acid rescue also works on synthetic medium at 25 °C. The area of the individual colonies in the red boxes in A) were measured from high-magnification photographs and plotted as arbitrary units.

Appendix Table S1. **Yeast strains used in this study**

Strain	Genotype	Reference
ByK45	BY4741 MATa his3Δ leu2Δ0 met15Δ0 ura3Δ0	Euroscarf
ByK830	w303 MATa	yDF126 - Peter Lab
ByK1148	ByK830 *psd1Δ psd2Δ*::KanMX *cho2Δ*::hphNT1 *opi3Δ*	This study
ByK1149	ByK1148 *csf1Δ*::His3MX6	This study
ByK1418	ByK45 CSF1::GFP- KANMX	This study
ByK1419	ByK45 CSF1::GFP- KANMX PEX10::mCHERRY-NAT	This study
ByK1420	ByK45 *csf1(737aa-endΔ)*	This study

Appendix Table S2. **Plasmids used in this study**

Plasmids	Genotype	Reference
Plasmids for rewiring:		
pBK586	pRS415-TEFpr- Bsc2(1-92)-pkPSD(35-end)-GFP	This study
pBK588	pRS415-TEFpr- pkPSD(35-end)-GFP-fyve (EEA1)	This study
pBK590	pRS415-TEFpr- Sec66(1-60)-pkPSD(35-end)-GFP	This study
pBK785	pRS415-PSDpr-Mic60(1-57)-scPSD(102-end)-GFP	This study
pBK556	pRS415-TEFpr-Su9(1-69)-aaPmt-GFP	This study
pBK557	pRS415-TEFpr-aaPmt-GFP(skl)	This study
pBK558	pRS415-TEFpr-aaPmt-GFP-fyve (EEA1)	This study
pBK559	pRS415-TEFpr-Coa3(1-49)-aaPmt-GFP	This study
pBK561	pRS415-TEFpr- Bsc2(1-92)-aaPmt-GFP	This study
pBK562	pRS415-TEFpr-Sec66(1-60)-aaPmt-GFP	This study
pBK786	pBK586 *Leu2Δ*::HIS3	This study
pBK787	pBK588 *Leu2Δ*::HIS3	This study
pBK788	pBK590 *Leu2Δ*::HIS3	This study
pBK789	pBK590 *Leu2Δ*::HIS3	This study
pBK790	pBK586 *Leu2Δ*::URA3	This study
pBK792	pBK785 *Leu2Δ*::URA3	This study
pBK793	pBK786 GFP(G65T G67A)	This study
pBK794	pBK787 GFP(G65T G67A)	This study
pBK795	pBK788 GFP(G65T G67A)	This study
pBK796	pBK789 GFP(G65T G67A)	This study
pBK797	pBK556 GFP(G65T G67A)	This study
pBK798	pBK557 GFP(G65T G67A)	This study
pBK799	pBK558 GFP(G65T G67A)	This study
pBK800	pBK559 GFP(G65T G67A)	This study

Appendix Table S2. continued **Plasmids used in this study**

Plasmids	Genotype	Reference
pBK801	pBK561 GFP(G65T G67A)	This study
pBK802	pBK562 GFP(G65T G67A)	This study
Other plasmids:		
pBK506	pRS415-TEFpr	[1]
pBK196	pRS424GFP-FYVE(EEA1)	[2] (Addgene plasmid # 36096)
pBK626	pRS316-*trp1*::miniDs GAL1pr-TPase	This study
pBK804	pRS413-ERG6pr-ERG6-mCHERRY	This study
pBK417	pRS413-TEFpr-mCHERRY-Ubc6(TA)	[3]
pBK64	pVTU100-mtBFP	[4]

Appendix Table S3. **Primers used in this study**

Name	Sequence (5' ⟶ 3')
**Primers used for gene deletions**	
CSF1 C' tag pYM F	CAAAAGCTTGTTTATCTTGCAGAAAAGCAGTATGTCAAGATACTAGATGATACGCATcgtacgctgcaggtcgac
CSF1 C' tag pYM R	ACATAAACCAGAAATATGGTATCAAGGACTTTTGAATATAATTAGGAACGAGATTAatcgatgaattcgagctcg
CSF1 KO pYM F	CATTAAAGCCACCTGACTCAAGTCTTCTATTGACGGTAATAAGTTAGCAAGCATGcgtacgctgcaggtcgac
CSF1_C'tag_797aa_f	ACGTCAGAAGAGTACACAGGTGTCCTTGGCGCTAGGGAAGTCGGAGATGTCACcgtacgctgcaggtcgac
CSF1_KO_797aa_f	ACGTCAGAAGAGTACACAGGTGTCCTTGGCGCTAGGGAAGTCGGAGATGTCACTAAcgtacgctgcaggtcgac
Psd2_pringle_F	GATGCTGTATCAATTGGTAAAGAATCCTCGATTTTCAGGAGCATCCAACGcgtacgctgcaggtcgac
Psd2_pringle_R	CTTGTTTGTACACGCTATAGTCTATAATAAAGTCTGAGGGAGATTGTTCATGatcgatgaattcgagctcg
Cho2_S1_knop	CTGAATATTTCGAGTGATTTTCTTAGTGACAAAGCTTTTTCTTCATCTGTAGATGcgtacgctgcaggtcgac
Cho2_S2_knop	TAACTTGAATCCTAGTACTTTTTAAATATATATACTCAAAAAAAAAAACTCAatcgatgaattcgagctcg
**Primers used for CRISPR-Cas9-mediated gene deletions**	
Psd1_gRNA_IntRev	ctagctctaaaacACCCCTTGATGTCTAAGAGTgatcatttatctttcactgcggagaag
Psd1_gRNA_Intfwd	ACTCTTAGACATCAAGGGGTgttttagagctagaaatagcaagttaaaataaggctagtccg
Psd1_link_IntRev	gtccgccggcgttggacgagcgGCTGGCTTTGCTTTTCCTTCTTCTTC
Psd1_link_IntFwd	ctcgtccaacgccggcggacctAAGCAATCATATGTAAAGTTAGCATTTATTTTGCTG
Psd1_-484up_f	GACTGGTACACCTGCAGGTGTAG
Psd1_-500dwSTOP_r	CACCTCTTCGCAACTGGTTGAAAG
Opi3_gRNA_fwd	gtttcggcgttcgaAACTTCTCCGCAGTGAAAGATAAATGATCcccgtgatcagagaacacgtGTTTTAGAGCTAGAAATAGCAAGTTAAAATAAGGCTAGTCCGTTATCAACTTGAAAAAG

Opi3_gRNA_rev	CTTTTTCAAGTTGATAACGGACTAGCCTTATTTTAACTTGCTATTTCTAGCTCTAAAACacgtgttctctgatcacgggGATCATTTATCTTTCACTGCGGAGAAGTTtcgaacgccgaaac
opi3_link_intRev	gtccgccggcgttggacgagcgTGCTTGACTTGCGCTATTCTTGTTG
opi3_link_intfwd	ctcgtccaacgccggcggacctCCTATGCTATTACCGTTTCTATATAGCTCC
opi3_407upATG_f	GGTGGCTAGTCCGTCTTCAAATTC
opi3_402dwSTP_r	CTGGTGACAATGGCACCGTTC

Primers used for colony PCR

Psd2 up	ACGCATGTGCTACTTCAAGG
Psd2 down	AAGGCCGAGAAGTACCTTTG
Psd1_-584up_f	CGG ACA GTT GAG ACA AGA TGG TGG
Psd1_-500dwSTOP_r	CACCTCTTCGCAACTGGTTGAAAG
CSF1_152upATG_f	CCACTATAAAGCTGTTGGCACGG
CSF1_2537dnATG_r	GAGCACCATCCCAAACCGAAAATG
CSF1_2137dnATG_f	CCCCTTGGAATACATTGAACGAATTC
KanMX_twd_5prime	CATGTTGGAATTTAATCGCGGCCTC
natNT2_+26_rev	CTGGTGCGGTACCGGTAAG
Nat -249Rev	GGATGTATGGGCTAAATGTAC

Primers used for plasmid construction

URA3_gr_fwd	GTGCGGTATTTCACACCGCATATCGACGGTCGAGGgagtgcaccataccacagcttttc
URA3_gr_rev	GTTTATGTACAAATATCATAAAAAAAGAGAATCTTTttagttttgctggccgcatcttc
HIS1_gr_fwd	GTGCGGTATTTCACACCGCATATCGACGGTCGAGGatgcgtacgctgcaggtcgac
HIS1_gr_rev	GTTTATGTACAAATATCATAAAAAAAGAGAATCTTTtaaatcgatgaattcgagctcg
G229A_G230C_G236C_f	gtatctcgcaaaacattgaacagcataagtgaaagtagtgactaaggttggc
G229A_G230C_G236C_r	gccaaccttagtcactactttcacttatgctgttcaatgttttgcgagatac
Psd1pr_gr_f	aaccctcactaaagggaacaaaagctggagctcTGAGACAAGATGGTGGTACTAACC
Psd1pr_Mic60_gr_r	aatttttcgtgaggcagtagttcttagcatcattctagaGCTGGCTTTGCTTTTCCTTCTTC
Mic60_f	atgatgctaagaactactgcctcac
mic60_yPSD_r	GATTTTTCTTGTCCTTCTCCCTTTTTTGCCCTCTGTAGCATCCTCCGAATATATGATACCTCCAGCGTAG
yPSD_AA102_f	GAGGATGCTACAGAGGGCAA
yPSD_NOSTOP_R	TTTTAAATCATTCTTTCCAATTATGCC
yPSD_HIND3-GFP-F	GGGACAGAAATTAGGCATAATTGGAAAGAATGATTTAAAAaagcttggagcaggtgctggtgctgg
pRS415_GFP_r	CTAATTACATGACTCGAGGTCGACGGTATCGATAAGCTTttatttgtacaattcatccataccatgggt
Aapmt_f	actagtATGAGTACCTCCAGACAAAGAGAAGATATG
Aapmt_Bsc2_r	CATATCTTCTCTTTGTCTGGAGGTACTCATactagtGATCGCGTCCAGTATGATAATAGGC
pRS415_Aapmt_r	CTAATTACATGACTCGAGGTCGACGGTATCGATAAGCTTtcagacaggcaagttttccaaagttac
Tef_Sec66_f	AAGCATAGCAATCTAATCTAAGTTTTCTAGAatgtccgaatttaatgaaacaaaattctcc
Aapmt_Sec66_r	CATATCTTCTCTTTGTCTGGAGGTACTCATactagtTGGTTGCTCACTAATTTTTTTGGCC
Tef_Aapmt_f	aagcatagcaatctaatctaagttttctagaATGAGTACCTCCAGACAAAGAGAAGATATG

p415_SKL_Aapmt_r	TTACATGACTCGAGGTCGACGGTATCGATaagcttTTAtaatttagaGACAGGCAAGTTTTCCAAAGTTACTAAG
Aapmt_rev	GACAGGCAAGTTTTCCAAAGTTACTAAGG
Aapmt-GFP_f	CCTTAGTAACTTTGGAAAACTTGCCTGTCactagtGGAGCAGGTGCTGGTGCTG
pYM25-GFP-r	tttgtacaattcatccataccatgggt
GFP_FYVE_f	acccatggtatggatgaattgtacaaaTGGCAATCTAGTCAACGGAGAGTTAG
FYVE_pRS415_r	ctaattacatgactcgaggtcgacggtatcgatTTATCCTTGCAAGTCATTGAAACATGCATC
Tef_Su9_f	AAGCATAGCAATCTAATCTAAGTTTTCTAGAatggcctccactcgtgtcctc
Aapmt_Su9_r	CATATCTTCTCTTTGTCTGGAGGTACTCATactagtGGAAGAGTAGGCGCGCTTCTG
Aapmt_Vac8_r	CATATCTTCTCTTTGTCTGGAGGTACTCATactagtATGTAAAAATTGTAAAATCTGTTGAGTAATATTATAC
GFP_AaPmt_r	AGCACCAGCACCAGCACCTGCTCCactagtGACAGGCAAGTTTTCCAAAGTTACTAAGG

Primers used for library preparation

P5_MiniDs	AATGATACGGCGACCACCGAGATCTACtccgtcccgcaagttaaata
P7_indexed_N701	CAA GCA GAA GAC GGC ATA CGA GAT TCG CCT TAA CGA AAA CGA ACG GGA TAA A
P7_indexed_N702	CAA GCA GAA GAC GGC ATA CGA GAT CTA GTA CGA CGA AAA CGA ACG GGA TAA A
P7_indexed_N703	CAA GCA GAA GAC GGC ATA CGA GAT TTC TGC CTA CGA AAA CGA ACG GGA TAA A
P7_indexed_N704	CAA GCA GAA GAC GGC ATA CGA GAT GCT CAG GAA CGA AAA CGA ACG GGA TAA A
P7_indexed_N705	CAA GCA GAA GAC GGC ATA CGA GAT AGG AGT CCA CGA AAA CGA ACG GGA TAA A
P7_indexed_N706	CAA GCA GAA GAC GGC ATA CGA GAT CAT GCC TAA CGA AAA CGA ACG GGA TAA A
P7_indexed_N707	CAA GCA GAA GAC GGC ATA CGA GAT GTA GAG AGA CGA AAA CGA ACG GGA TAA A
P7_indexed_N710	CAA GCA GAA GAC GGC ATA CGA GAT CAG CCT CGA CGA AAA CGA ACG GGA TAA A
P7_indexed_N711	CAA GCA GAA GAC GGC ATA CGA GAT TGC CTC TTA CGA AAA CGA ACG GGA TAA A
P7_indexed_N712	CAA GCA GAA GAC GGC ATA CGA GAT TCC TCT ACA CGA AAA CGA ACG GGA TAA A
P7_indexed_N714	CAA GCA GAA GAC GGC ATA CGA GAT TCA TGA GCA CGA AAA CGA ACG GGA TAA A
P7_indexed_N715	CAA GCA GAA GAC GGC ATA CGA GAT CCT GAG ATA CGA AAA CGA ACG GGA TAA A
P7_indexed_N716	CAA GCA GAA GAC GGC ATA CGA GAT TAG CGA GTA CGA AAA CGA ACG GGA TAA A
P7_indexed_N718	CAA GCA GAA GAC GGC ATA CGA GAT GTA GCT CCA CGA AAA CGA ACG GGA TAA A
P7_indexed_N719	CAA GCA GAA GAC GGC ATA CGA GAT TAC TAC GCA CGA AAA CGA ACG GGA TAA A
P7_indexed_N720	CAA GCA GAA GAC GGC ATA CGA GAT AGG CTC CGA CGA AAA CGA ACG GGA TAA A
P7_indexed_N721	CAA GCA GAA GAC GGC ATA CGA GAT GCA GCG TAA CGA AAA CGA ACG GGA TAA A

P7_indexed_N722	CAA GCA GAA GAC GGC ATA CGA GAT CTG CGC ATA CGA AAA CGA ACG GGA TAA A
P7_indexed_N723	CAA GCA GAA GAC GGC ATA CGA GAT GAG CGC TAA CGA AAA CGA ACG GGA TAA A
P7_indexed_N724	CAA GCA GAA GAC GGC ATA CGA GAT CGC TCA GTA CGA AAA CGA ACG GGA TAA A
P7_indexed_N726	CAA GCA GAA GAC GGC ATA CGA GAT GTC TTA GGA CGA AAA CGA ACG GGA TAA A
P7_indexed_N727	CAA GCA GAA GAC GGC ATA CGA GAT ACT GAT CGA CGA AAA CGA ACG GGA TAA A
P7_indexed_N728	CAA GCA GAA GAC GGC ATA CGA GAT TAG CTG CAA CGA AAA CGA ACG GGA TAA A
P7_indexed_N729	CAA GCA GAA GAC GGC ATA CGA GAT GAC GTC GAA CGA AAA CGA ACG GGA TAA A
**Primers used for sequencing**	
688_minidsSEQ1210	tttaccgaccgttaccgaccgttttcatcccta
Custom_index1	GGT TTT CGA TTA CCG TAT TTA TCC CGT TCG TTT TCG T

References

1. Mumberg, D., Müller, R., & Funk, M. (1995). Yeast vectors for the controlled expression of heterologous proteins in different genetic backgrounds. *Gene*, *156*(1), 119-122.

2. Burd, C. G., & Emr, S. D. (1998). Phosphatidylinositol (3)-phosphate signaling mediated by specific binding to RING FYVE domains. *Molecular cell*, *2*(1), 157-162.

3. John Peter, A. T., Herrmann, B., Antunes, D., Rapaport, D., Dimmer, K. S., & Kornmann, B. (2017). Vps13-Mcp1 interact at vacuole–mitochondria interfaces and bypass ER–mitochondria contact sites. *Journal of Cell Biology*, *216*(10), 3219-3229.

4. Westermann, B., & Neupert, W. (2000). Mitochondria-targeted green fluorescent proteins: convenient tools for the study of organelle biogenesis in Saccharomyces cerevisiae. *Yeast (Chichester, England)*, *16*(15), 1421–1427
