# Supplementary material for: Rewiring phospholipid biosynthesis reveals resilience to membrane perturbations and uncovers regulators of lipid homeostasis
Source: EMBO J. 2022 Feb 21;41(7):e109998. doi: 10.15252/embj.2021109998 (PMC8982615; doi:10.15252/embj.2021109998)
Supplement: Supplementary file 2 — Expanded View Figures PDF [file EMBJ-41-e109998-s003.pdf]

Expanded View Figures

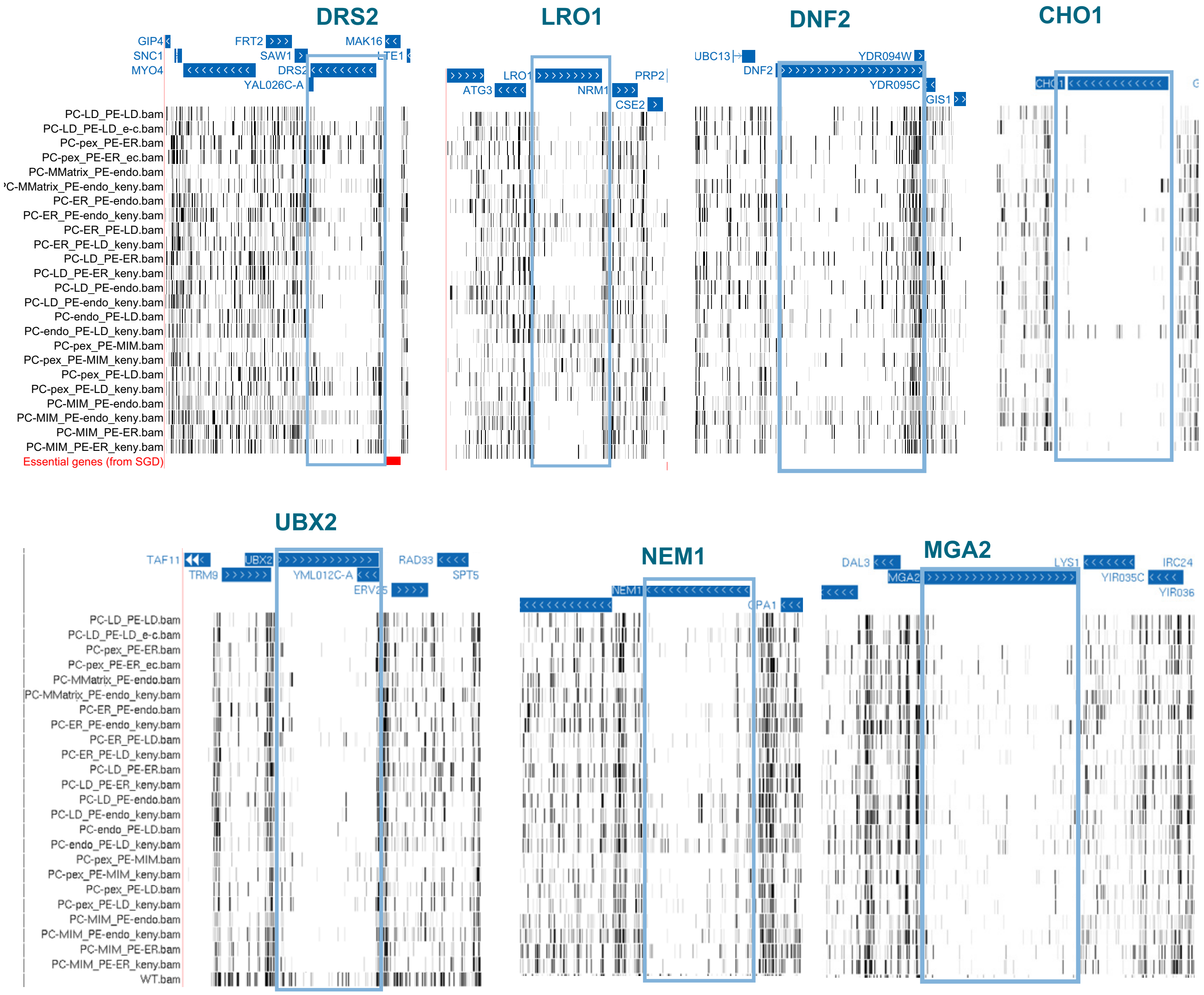

Figure EV1. Lipid metabolism and lipid flippases genes are required for adaptation in specific rewired conditions.

Transposon insertion maps of the *DRS2*, *LRO1*, *DNF2*, *NEM1*, and *MGA2* genomic loci generated in the UCSC genome browser for libraries of strains expressing the chimeric Psd and Pmt enzymes to produce PE and PC at the indicated cellular locations in the *choppΔ* background. "KennedyON" refers to libraries that were grown in media supplemented with 10 mM ethanolamine and choline.

 © 2022 The Authors

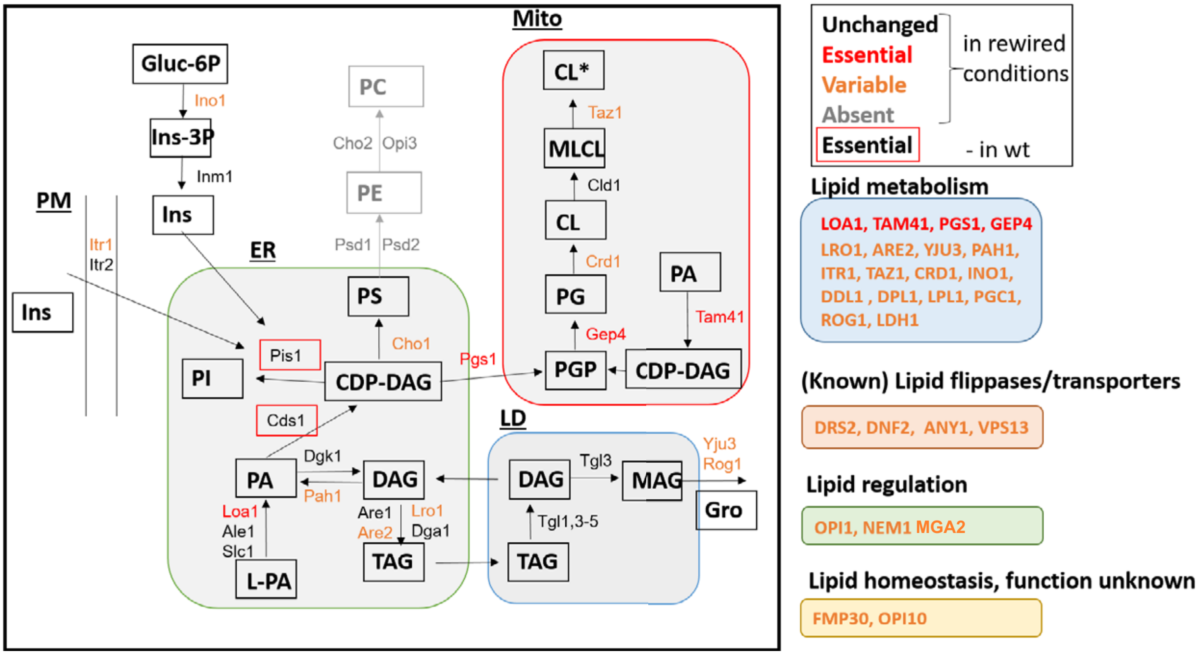

Figure EV2. Requirement for lipid metabolic genes in rewired yeast.

Schematic illustration of lipid synthesis pathways in yeast. Genes that are essential or absent in all libraries are depicted in red or gray, respectively. Genes variably required in one or more rewired libraries, as assessed by manual inspection or volcano plots, are depicted in orange. Genes that are essential in wild-type conditions are boxed in red.

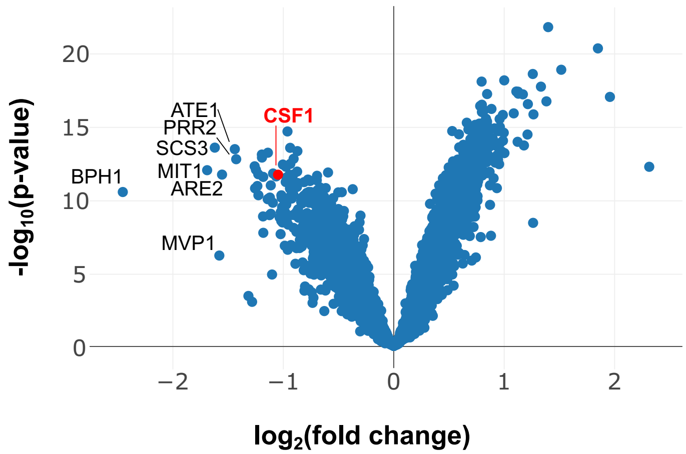

Figure EV3. The requirement for Csf1 is specific to the Psd-MIM/Pmt-pex library grown in Kennedy$_{OFF}$ conditions.

Volcano plot comparing the number of transposon insertions per gene in the Psd-MIM / Pmt-pex Kennedy$_{OFF}$ condition versus all other libraries. *CSF1* is highlighted in red.

© 2022 The Authors

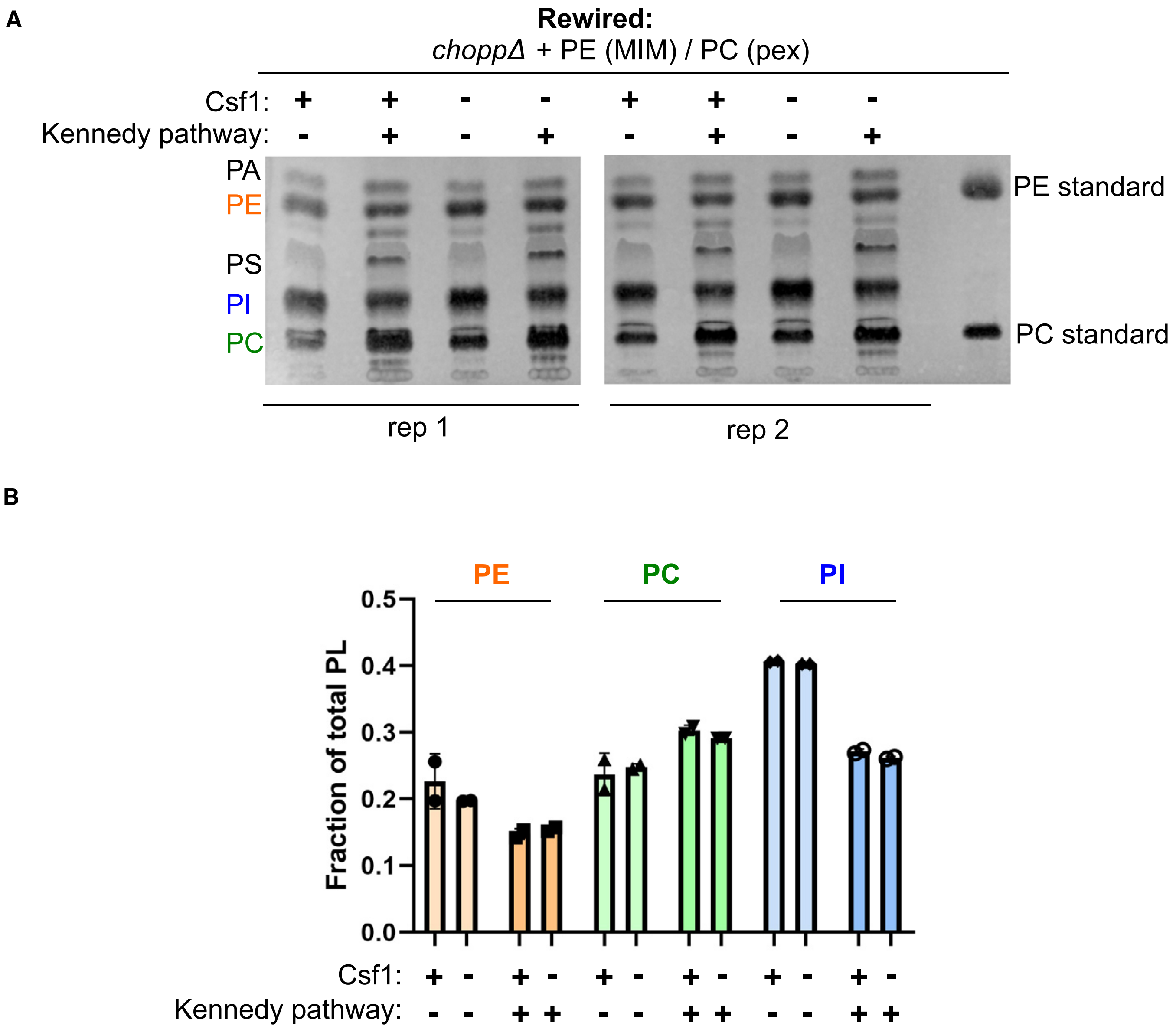

Figure EV4. The absence of Csf1 in rewired conditions does not affect steady state PE or PC production.

A Thin-layer chromatography (TLC) analysis of the steady-state lipid profiles of the indicated strains grown in Kennedy$_{OFF}$ or Kennedy$_{ON}$ conditions (+10 mM ethanolamine and choline).

B Quantification of the fraction of PE, PC, and PI of total phospholipids measured by TLC analysis shown in A. Quantification was done using Fiji/ImageJ software as described in the Materials and Methods.

Figure EV5. Rate of growth and ^{15}N incorporation in PS, PE, and PC during the experiment shown in Fig 6C.

A OD_{600} measurements of the indicated genotypes at different time points taken during the ^{15}N-serine labeling experiment.

B Line plots depict quantification of the amount of indicated ^{15}N-labeled species normalized by the amount of its corresponding unlabeled species.

Source data are available online for this figure.

© 2022 The Authors

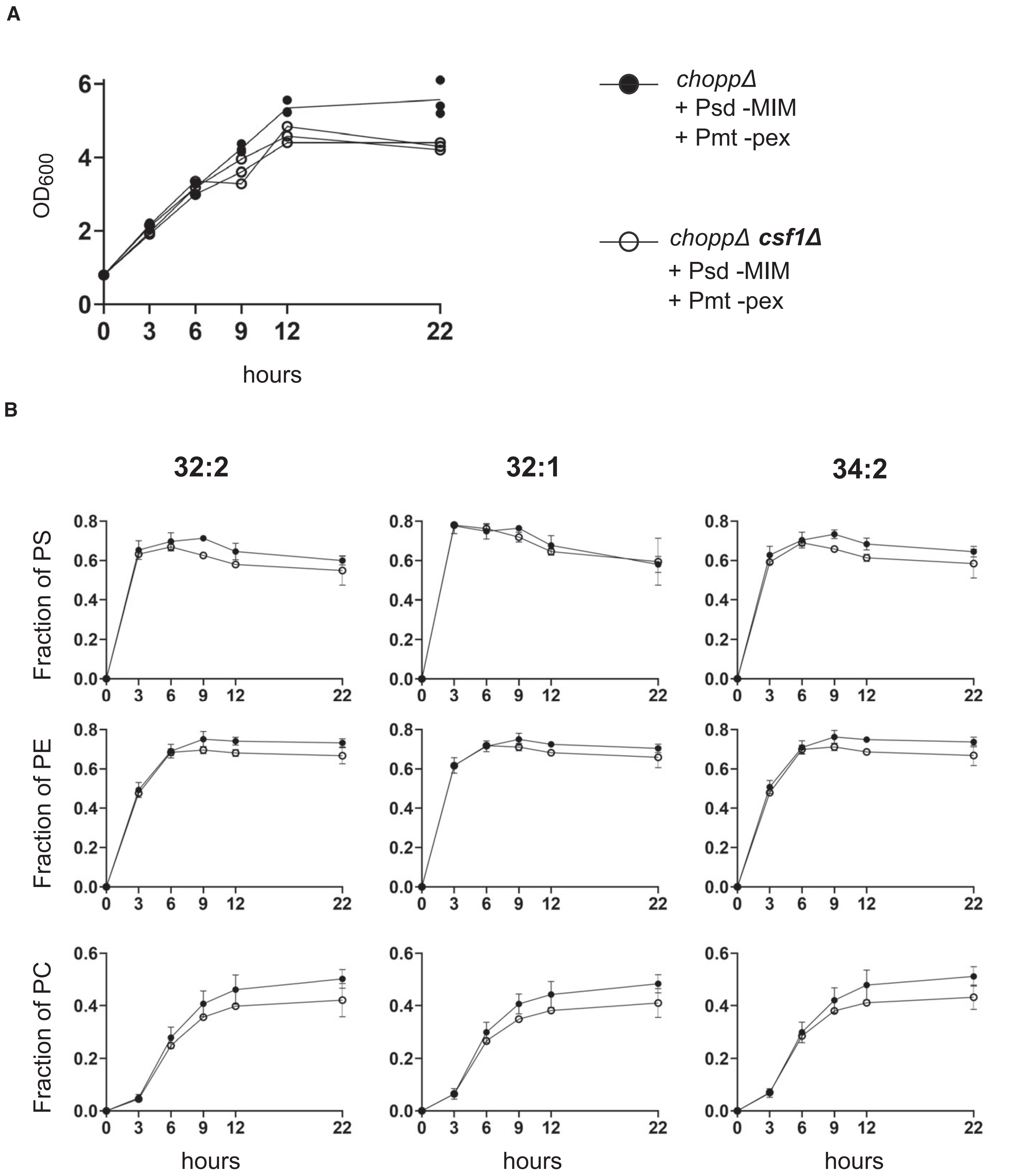
A
6
4
2
0
OD600
0
3
6
9
12
22
hours
choppΔ
+ Psd -MIM
+ Pmt -pex
choppΔ csf1Δ
+ Psd -MIM
+ Pmt -pex
B
32:2
32:1
34:2
Fraction of PS
Fraction of PE
Fraction of PC
0.8
0.6
0.4
0.2
0.0
hours

Figure EV5.
