# Supplementary material for: Rewiring phospholipid biosynthesis reveals resilience to membrane perturbations and uncovers regulators of lipid homeostasis
Source: EMBO J. 2022 Feb 21;41(7):e109998. doi: 10.15252/embj.2021109998 (PMC8982615; doi:10.15252/embj.2021109998)
Supplement: Supplementary file 7 [file EMBJ-41-e109998-s006.pdf]

THE EMBO JOURNAL

Rewiring Phospholipid Biosynthesis Reveals Resilience to Membrane Perturbations

Arun John Peter, Sabine van Schie, Ngaam Cheung, Agnes Michel, Matthias Peter, and Benoit Kornmann
DOI: 10.15252/embj.2021109998

Corresponding authors: Benoit Kornmann (benoit.kornmann@bioch.ox.ac.uk) , Arun John Peter (arun.johnpeter@unifr.fr)

Review Timeline:

Transfer from Review Commons:	20th Oct 21
Editorial Decision:	17th Nov 21
Revision Received:	20th Dec 21
Accepted:	7th Jan 22

Editor: Daniel Klimmeck

Transaction Report:

This manuscript was transferred to The EMBO Journal following peer review at Review Commons.

(Note: With the exception of the correction of typographical or spelling errors that could be a source of ambiguity, letters and reports are not edited. Depending on transfer agreements, referee reports obtained elsewhere may or may not be included in this compilation. Referee reports are anonymous unless the Referee chooses to sign their reports.)

Review #1

1. How much time do you estimate the authors will need to complete the suggested revisions:

Estimated time to Complete Revisions (Required)

(Decision Recommendation)

Less than 1 month

2. Evidence, reproducibility and clarity:

Evidence, reproducibility and clarity (Required)

The manuscript from Peter and Van Schie is clear to me (and I am not a yeast geneticist) and well-written. The introduction presents the current state of knowledge on MCS and lipid fluxes in yeast, introduces the two PL synthesis pathways and explain the different level of redundancy existing in phospholipid biosynthesis and their distribution through different MCS in yeast. The principle of rewiring or perturbing lipid fluxes in cells is not novel per se as the Authors very adeptly mention in their introduction but it is here applied to the two important PL "membrane building blocks" PE and PC in a remarkably systematic and thorough way. To my knowledge a study that systematic is a first.

The Authors from the Peter and Kornmann groups rewire the production of two main phospholipids PE and PC in yeast by redirecting their respective biosynthesis to different subcellular compartments using chimeras of enzymes-organelle targeting signals and characterize the ensuing adjustments and adaptations.

This beautiful study by the is very thorough and represent an impressive accomplishment. 24 rewired yeast strains were engineered where PE and PC synthesis were retargeted in different organelles. The Authors observe that all rewired strains are viable and that PE and PC PLs are still produced following a new rewired pathway, although they also notice difference in overall lipid profiles using bulk TLC at steady state. They conclude that cells have transport mechanisms to redistribute lipids now produced at these 'un-natural' locations towards the rest of the cell membranes. This demonstrates the robustness of yeast cell lipid metabolism and the existence of unsuspected layers of adaptation/lipostasis and compensatory mechanisms.

The Authors next apply saturated transposon-based mutagenesis on 12 pairs of their rewired strains to identify genes necessary to adapt to such lipid rerouting. This is impressive work and extensive analysis. Figure 3 is very clear and explains the principle quite efficiently for non-aficionados like me. Their essential gene identification methodology seems validated with their observation on the Sam5 methyl-(SAM) donor transporter in the inner mitochondrial is required to maintain viability (tri-methylate PE into PC) in rewired conditions (PE-endo/PC-mm). The genes identified are enriched

for lipid and membrane-associated processes. LTP-related genes are expected to be enriched to adapt to rewired conditions, this is validated with the observation made on Vps13, a known tether and LTP.See my comment C2
Several genes are identified in particular a cluster of essential genes involved in protein sorting and vesicular trafficking.

In particular they discuss the role of Csf1 as a protein enabling lipid homeostasis adaption.
The cases of Mdm31 and Csf1 are next discussed. Both contain a Chorein-N motif previously characterized at the functional (lipid transport and inter-organelle tether) and structural levels in the proteins Atg2 and Vps13 involved in the bulk transfer of lipids between ER and phagosome or at certain organelle contact sites. Csf1 is specifically required in some rewired strains PE-mim/PC-pex. Through N15-serine pulse-chase experiments, the authors rule out an LTP function for Csf1 between ER-mitochondria and peroxisomes as it does not localize at these contact sites but do not dismiss that function at other contact sites. The cold-sensitivity phenotype observed is linked to a lack of adaptability of this mutant in remodeling lipid chain-length (lack of shortening) and insaturation levels (lack of increasing) in response to the drop in temperature to adjust membrane fluidity. See my comment C3
The discussion is well presented. The value of this work is double. As a proof of concept to use lipid biosynthesis rewiring combined to SATAY to uncover general requirements for coping with membrane-related stress to identify proteins involved adapting to new conditions. The characterization of Csf1, albeit preliminary, paves the way to future studies.

I have no major comments.

The manuscript is quite clear and well-written.

A few minor comments.

C1: Abstract. The term 'lipid regulatory player' reads a bit awkward to me, though I will leave this to a native English speaker/writer to be a final judge of my comment.

C2: I would suggest adding a Figure in panel 3C to explain the Vsp13 case in a graphical way (like what is done for Sam5 in panel 3D)

C3: The differences analyzed by MS-lipidomics and discussed in figure 7E don't look very striking though; with 3 replicates for csf1delta and 2 replicates for wt cells, the error levels don't seem to be clearly displayed. The Authors may want to quantify the statistic aspect of these measurements a bit more adequately, although I am aware these are sensitive experiments.

C4: Besides the chorein-N motif identified in Csf1, are there any other domains identifiable in this very large protein that could provide some more insights into their discussion about the mechanism of action of Csf1.

C5 more of question actually. Out of pure curiosity, why choose the Psd from such an

awkward and quite 'off-putting' organism such as the primate malaria parasite P. knowlesi?

3. Significance:

Significance (Required)

Membrane contact sites and the mechanisms of lipid distribution between organelle is an area of intense research. The model system here is yeast, but with constant references to metazoans

The study is remarkable by its thorough, extensive and systematic nature.
Perturbing lipid fluxes by rewiring through the specific retargeting of biosynthetic enzymes is performed in a systematic rigourous way to uncover novel regulatory mechanisms and reveal the remarkable robustness in membrane homeostasis

The work is of general interest to cell biologists and biochemists in the field of organelle biogenesis and lipid metabolism/trafficking whether it is by non-vesicular (LTP-based) or vesicular routes. It is also a (very) elegant and smart use of saturation transposon mutagenesis to study, uncover and identify the role of genes (and their products) on cellular processes and the adaptability of cells (yeast) in this case in response to (lipid) homeostatic perturbations.
The availability of these extensive genetic datasets as a source of data for future studies is now made available to the community.

I am a biochemist working on protein and lipid translocation across membranes and between membrane at MCSs in eukaryotic systems in particular at mitochondria-attached membranes. Even for a non-geneticist as me, I think the Authors present their results and data in a quite clear and convincing way.

Review #2

1. How much time do you estimate the authors will need to complete the suggested revisions:

Estimated time to Complete Revisions (Required)

(Decision Recommendation)

Less than 1 month

2. Evidence, reproducibility and clarity:

Evidence, reproducibility and clarity (Required)

Summary:

In this manuscript Arun et al study the functional plasticity of membrane and lipid homeostasis in yeast. They first generate strains the lag the ability to synthesize PE and PC via the PS decarboxylation pathway, thus making the Kennedy Pathway essential. Therefore, the strain can only survive when supplemented with ethanolamine and choline. Next the authors come up with a smart idea to fuse a non-transmembrane containing PS-decarboxylase or a PE methyltransferase to short organelle targeting sequences. In this way the authors can physically separate PE and PC biosynthesis and thus making the biosynthesis depending on lipid transport between the different compartments. To identify such lipid transfer and lipid metabolism genes the use SATAY screens that have been developed and perfected in the Kornmann laboratory. Now the authors compare a variety of conditions where PE and PC biosynthesis are targeted to multiple organelles. They provide very rich datasets showing specific requirements for several proteins under their rewired conditions. In the end the authors focus on one protein Csf1 that was identified under specific conditions in their screen and analyze its role in lipid metabolism.

Major comments:

This a very well written manuscript that will function as an important resource for the growing lipid metabolism and contact site communities. In addition, systematic analysis of the functional plasticity of membranes and lipid metabolism will be essential in the future. The flexibility of lipid metabolism (and metabolism in general) makes it difficult to study with classical cell biological methods. The authors make it very clear that this manuscript will be a starting point for many future studies to come and should thus be published prominently. The generated datasets are very convincing and it is very helpful that all datasets are provided and also accessible on the homepage provided by the Kornmann lab. The statistical analyses appear to be very convincing.
The only weaker part of the manuscript is the characterization of Csf1 in lipid homeostasis. The authors nicely show that CSF1 becomes essential under conditions that require the supplementation of choline and ethanolamine. The generation of the C-terminal deletion allele is also very elegant regarding the surrounding essential genes. However I have some concerns regarding the pulse chase labeling of lipids with heavy labeled serine. Usually the flux analysis using stable isotope containing amino acids is also a smart way to study lipid flux. My concern is the conditions under which the experiment was conducted. The authors show that without supplementation of choline and ethanolamine in csf1Δ (cells are barely growing in spotting assays. Yet, the authors perform the labeling for 22h under these conditions. The following questions thus arise:

-can the authors provide growth curves for the two/three strains (WT csf1ΔC and csf1Δ) under the conditions used

-What are the total amounts of PE and PC synthesized? Can the authors also provide the raw data here to show the total synthesis rates?

-For these experiments the authors should provide the exact composition and amounts of "light" and "heavy" serine present in the medium

-one important control for this experiment should also be the rate of autophagy appearing under the used growth conditions. If csf1Δ cells are starving under the used conditions, this could lead to mitophagy which would then feed heavy labeled PE into other pathways. A quick search in the HIP/HOP databases lists atg17Δ as the highest correlating deletion with csf1Δ.

Figure 7d and e:

-Why are only two replicates shown for some of the conditions?

Minor comments:

I also have a few small suggestions that I think would help to improve the impact of the manuscript. One small suggestions I have is that the labeling of the different conditions in the online searchable data becomes somewhat more easily comprehensible. And, if possible, I would suggest to make genes searchable in the datasets. Many researchers would be interested in a specific gene they are working on. Therefore this would be very beneficial for the study.

Page 11: The gene name DFN2 should probably read DNF2

3. Significance:

Significance (Required)

This manuscript addresses the flexibility of the lipid metabolic network using high throughput genetic screening. The data provided here will be very useful for a diverse community of researchers interested in lipid metabolism, lipid transport and organelle contact sites. As an expert in lipid and membrane homeostasis I am convinced that this will be an extremely useful resource and will be the starting point for many future studies on membrane plasticity and lipid homeostasis.

Review #3

1. How much time do you estimate the authors will need to complete the suggested revisions:

Estimated time to Complete Revisions (Required)

(Decision Recommendation)

Between 1 and 3 months

2. Evidence, reproducibility and clarity:

Evidence, reproducibility and clarity (Required)

John Peter et al. perform a very systematic rewiring of phospholipid metabolism in the budding yeast model. Starting with a strain that is blocked in synthesis of PC and PE via the CDP-DAG pathway, they then introduce a plasmodium PS decarboxylase (to produce PE) and a bacterial PE methyltransferase fused to different targeting sequences to target the two enzymes to different cellular locations (24 combinations in total). It is impressive that in all cases the CDP-DAG pathway is functional. After this sort of metabolic rewiring, they then ask how cells cope with changes in the site of synthesis of PC or PE by performing genetic screens utilizing a transposon approach that was previously developed in the Kornmann lab (screening for genes that become required in different rewired conditions).

Whereas the approach to target a heterologous PE/PC synthase to a different yeast compartment is not new, this study is impressive in the thoroughness of the initial design and the amount of data that is produced and analyzed. However, the large number of conditions tested is in a way also a weakness because the authors make some assumptions that are not verified or that are not confirmed by the results of the study, so the interpretation is less thorough than the approach. The wealth of data produced can be mined by future work but a more thorough and critical assessment of the rewired strains would be beneficial for this and future analyses of these datasets. The example of Csf1 shows that the location of enzymes can have other effects besides where the product is made, or that cells can adapt in different ways to changes in lipid metabolism.

Specific comments:

1.One assumption is that PC and PE are both essential. The production of PC and PE (as well as PS an PI) is tested in different strains in a largely qualitative way, although some differences in phospholipid levels are observed (but this is not so clear from the TLC plates).

-It would be useful to know if all rewired strains require both ethanolamine and choline for growth or if only one supplement might be sufficient in some cases. Similar, are both pkPsd and aaPmt required in all cases or could the supplement of one enzyme be sufficient? For example, work from de Kroon lab has shown that it is possible for yeast to survive without PC and they compensate by acyl chain shortening and saturation; see Bao et al., biorXiv 2021 https://doi.org/10.1101/2021.02.08.429707 and Boumann et al., Mol Biol Cell 2006. This work is also relevant for my comment #2 and for the discussion about the role of Csf1.

2.Another assumption repeated throughout the study is that rewired strains will cope with changes in the location of pkPsd and aaPmt by adapting their lipid transport pathways, specifically by adapting the levels of lipid transport proteins to bring PC and PE to their native location. In other words, that what is different between the strains is only where PC and PE are initially produced, not the membrane composition, which is checked by a single TLC analysis, therefore only for the main phospholipid species for whole cell lysates. The analysis by transposon mutagenesis of the rewired strains does reveal that some lipid transfer proteins become required in some cases, in particular

Vps13, but it doesn't seem that lipid transfer proteins are highly represented in the transposon screens (the requirement for flippases could be due to changes in membrane composition). In particular, the hypothesis that the poorly characterized protein Csf1 is a novel lipid transfer protein is not confirmed. Therefore, the whole manuscript would benefit from being written in a more open-ended manner with regards to how cells could cope with rewiring of their phospholipid metabolism, which could be different in different cases (as demonstrated for Csf1) and additional experiments to better characterize lipid composition/membrane properties of the rewired strains would be highly beneficial. Besides plasticity and redundancy of lipid transport pathways (at least in standard lab growth conditions), there are also compensatory effects between different lipids in membranes.

-A more quantitative lipid analysis of rewired strains would be highly beneficial -- I realize that this is a lot of work, but this could add a lot of value to the subsequent genetic analyses. Less demanding phenotypic analyses of rewired strains could also be very informative, for example growth assays like those shown in Fig. 2A performed at different temperatures, which could be informative of changes in membrane composition (as shown later for csf1 mutants). Also, yeast often adapt to changes in phospholipid composition by producing more or less lipid droplets, so a quantification of lipid droplets by fluorescent microscopy or a quantification of neutral lipids could be informative of changes in membrane composition. These simple assays can be used to screen all rewired strains and then only strains displaying some phenotypic differences could be subjected to quantitative lipid analysis.
- Conclusions regarding lipid transport in the Discussion are too strong.

3.A third assumption is that the steady-state localization of pkPsd and aaPmt constructs (based on the GFP signal) reflects the only sites of activity of the two enzymes. I realize that this assumption would be very difficult to test, but the authors should at least consider the possibility that PC and PE could also be produced at other locations than at the desired destination, in particular at the ER.

-What is the expected localization of pkPsd and aaPmt in different constructs with respect to cytosol/lumen? This should be stated. Is orientation of the active site preserved with respect to the native enzymes in different cases? (Diagrams in Fig. 1A imply that PE and PC would be produced in the lumen of the endosome or peroxisome?)

-Many of the constructs would be expected to traffic through the ER to reach their final destination and one could imagine that a fraction of their enzymatic activity occurs at the ER, even if the GFP signal is not observed at the ER, or that a small ER fraction is responsible for all PE/PC production. We do not know how active these enzymes are compared to yeast Psd and Cho2/Opi3, or how their level of expression compares to the expression of native yeast proteins. Furthermore, since ER is in contact with all other locations except for MIM and MM, some of the pkPsd or aaPmt constructs could be acting in trans and still producing PE/PC on the ER membrane.

-In the labels in Fig 2A, it would be more fair to say 'Psd localization' and 'Pmt localization' instead of 'PE source' and 'PC source', and the authors should address possible caveats.

4.The Csf1 finding and its possible role in acyl chain remodeling is intriguing, although inconclusive and will require future studies.

Minor comments:

-The authors became interested in Csf1 due to the chorein-N domain homology; however it should be noted that the function of this domain is not known and it is not sufficient for lipid transport in Vps13.

-It would be informative to also mention the large size of Csf1; is it predicted to be soluble? Localization?

-Top of page 19: the authors present no evidence that Csf1 function is affecting lipid distribution, so this sentence should be corrected.

-With respect to what was previously known about Csf1, two studies uncovered csf1Δ in screens for genes involved in the maintenance of ER homeostasis/protein folding and secretion (see Copic et al., 2009, Genetics 182, 757 & Jonikas et al., 2009, Science 323, 1693), including a synthetic interaction with hac1Δ. Given the established connections between ER membrane composition/stress and ER homeostasis, these phenotypes seem highly relevant, and the statement on page 19 that the only well-documented phenotype of csf1 mutants is cold sensitivity should be amended.

-Intro, first paragraph: 10-30 nm, not nM

-Intro, last paragraph: correct 'minimalizing'

-scPsd instead of pkPsd in MIM panel of Fig. 1B ?

3. Significance:

Significance (Required)

Whereas the approach to a heterologous PE/PC synthase to a different yeast compartment is not new, this study is impressive in the thoroughness of the initial design and the amount of data that is produced and analyzed. As such, it represents a technical advance. The finding that Csf1 may be involved in acyl chain remodeling is intriguing but preliminary.

The study produces a wealth of data that can be used by other researchers with expertise in yeast genetics and lipid metabolism. Given the intricacy of lipid transport pathways and compensatory effects, a clear-cut conclusion could hardly be expected, which is also why the authors probably finally decided to focus on a single gene (which could be a paper in itself, if they decided to study it further).

(My expertise is in lipid transport and membrane traffic in yeast)

Full Revision

Manuscript number: RC-2021-00962
Corresponding author(s): Benoit, Kornmann
Arun Thomas, John Peter

1. General Statements [optional]

Dear Editor,

We were thrilled to see that all three reviewers found our study “impressive”, “systematic” and “thorough”, and found it to be “of general interest” (Reviewer 1), “an extremely useful resource” (Reviewer 2), producing “a wealth of data that can be used by other researchers” (Reviewer 3). We also note the various points made by the reviewers (addressed below) and agree with the limitations that they see in our study, in particular, the fact that we do not address the mechanism by which Csf1 affects lipid homeostasis, and therefore that the investigations on Csf1 remain exploratory. As stated by reviewer 3 “a clear-cut conclusion could hardly be expected” at this stage. Therefore, we took extra care at wording the study’s limitations and the exploratory nature of our Csf1 investigations. Importantly, we have now obtained new data on Csf1 establishing a causal link between the lipid homeostatic defect and the cold sensitivity in Csf1 mutants. Indeed, our previous manuscript showed a correlation between a defect in lipid remodeling upon cold treatment and an overall cold sensitivity phenotype. We now show that manipulating the mutant’s lipidome via oleic acid supplementation substantially rescues the cold sensitivity, demonstrating that the repercussions of Csf1’s absence on the lipidome lie upstream in the chain of events causing cold-sensitivity.
In terms of additional new data, we have added control quantifications and growth curves related to the ^{15}N-Serine labeling experiments (Reviewer 2, points 1-3). We have tested the necessity of ethanolamine, choline and the expression of both PSD and PMT to allow robust growth (Reviewer 3, point 1).
We have labeled the most important revisions in the text in red.
We think that, with the help of the reviewers, we have been able to write a more rigorous manuscript and are confident that it will have a major influence on lipid biology.

2. Point-by-point description of the revisions

Reviewer 1.

A few minor comments.

C1: Abstract. The term 'lipid regulatory player' reads a bit awkward to me, though I will leave this to a native English speaker/writer to be a final judge of my comment.

We agree (not being native speakers ourselves!) and have changed the title.

C2: I would suggest adding a Figure in panel 3C to explain the Vsp13 case in a graphical way (like what is done for Sam5 in panel 3D)

We followed this sound advice.

C3: The differences analyzed by MS-lipidomics and discussed in figure 7E don't look very striking though; with 3 replicates for csf1delta and 2 replicates for wt cells, the error levels don't seem to be clearly displayed. The Authors may want to quantify the statistic aspect of these measurements a bit more adequately, although I am aware these are sensitive experiments.

Although modest in absolute fold change, these differences are highly reproducible, and in the range of what was observed before for yeast grown at different temperatures (see e.g. https://pubmed.ncbi.nlm.nih.gov/22529973/). We now show each individual measurement point and computed statistical tests for WT vs. Csf1, all of which turned out to be very significant at 16 °C, some remain significant even at 30 °C, even with the limited number of replicates.

C4: Besides the chorein-N motif identified in Csf1, are there any other domains identifiable in this very large protein that could provide some more insights into their discussion about the mechanism of action of Csf1.

There is an N-terminal TM domain (shown in fig 6A), the length of which is consistent with ER insertion. Unfortunately, even AlphaFold could not predict a structure for the rest of the protein. We have developed a coevolution-based approach which predicts that the rest of the protein actually folds into a N-Chorein-like lipid tunnel despite no sequence homology. This finding will be published separately with the computing method (Cheung et al. in preparation).

C5 more of question actually. Out of pure curiosity, why choose the Psd from such an awkward and quite 'off-putting' organism such as the primate malaria parasite P. knowlesi?

For two reasons; first, proof that it works in S. cerevisiae was already provided in Choi et al. (duly referenced in the manuscript). Second, the same study provided evidence that a truncated allele of pkPSD lacking the TM domain remains active in S. cerevisiae, making its redirection easier.

Reviewer 2

This a very well written manuscript that will function as an important resource for the growing lipid metabolism and contact site communities. In addition, systematic analysis of the functional plasticity of membranes and lipid metabolism will be essential in the future. The flexibility of lipid metabolism (and metabolism in general) makes it difficult to study with classical cell biological

methods. The authors make it very clear that this manuscript will be a starting point for many future studies to come and should thus be published prominently. The generated datasets are very convincing and it is very helpful that all datasets are provided and also accessible on the homepage provided by the Kornmann lab. The statistical analyses appear to be very convincing.

We appreciate these very positive comments.

The only weaker part of the manuscript is the characterization of Csf1 in lipid homeostasis. The authors nicely show that CSF1 becomes essential under conditions that require the supplementation of choline and ethanolamine. The generation of the C-terminal deletion allele is also very elegant regarding the surrounding essential genes. However I have some concerns regarding the pulse chase labeling of lipids with heavy labeled serine. Usually the flux analysis using stable isotope containing amino acids is also a smart way to study lipid flux. My concern is the conditions under which the experiment was conducted. The authors show that without supplementation of choline and ethanolamine in csf1Δ (cells are barely growing in spotting assays. Yet, the authors perform the labeling for 22h under these conditions.

This is a fair concern. We are comparing strains with massively different health statuses since one is able to grow while the other isn't. This concern could have invalidated any conclusion, since any difference detected in these conditions could have been due to knock-on effects of the difference in health rather than to differences in lipid transport. However, we did not find any difference, and do not claim to have found any. In fact, lipid conversion curves are completely superimposable, not because, but in spite of the fact that the strains grow at different rates. Therefore, we feel comfortable concluding that Csf1 deletion does not, by and large, abrogate exchange from the Psd to the Pmt enzyme. That said, the reviewer makes an excellent point below, which shows that we have not taken enough care at explaining this experiment. The outcome of the experiment is a negative result, which are always difficult to interpret, but given that this experiment was the logical next step in our approach, we could hardly escape showing the results, albeit negative. But we did not put the necessary effort into explaining the experiment. We amended the text in several places and explain the details below while addressing the reviewer's comment.

The following questions thus arise:

-can the authors provide growth curves for the two/three strains (WT csf1ΔC and csf1Δ) under the conditions used

The growth curves have been added to Fig. S21A. As shown, there is no detectable growth defect in the initial timepoints, but csf1 mutant cells saturate at a slightly lower OD. While this result contrasts with the spotting assays of fig 6B, it is explained by the fact that cells were cultivated in presence of ethanolamine and choline, then immediately pulsed with labeled serine at an intermediate OD of 0.8 in the absence of both ethanolamine and

choline. Therefore, N15-Serine incorporation into PS, and its conversion to PE and PC happens in conditions where the effects of ethanolamine and choline withdrawal are not yet felt. This was done precisely to avoid problems with comparing growing and non-growing cells. The presence of remaining ethanolamine and choline (and residual Kennedy pathway activity) does not however affect the measurements in this experiment, since ^{15}N-serine incorporation exclusively labels PE and PC made through the CDP-DAG pathway.

-What are the total amounts of PE and PC synthesized? Can the authors also provide the raw data here to show the total synthesis rates?

We now provide the raw proportion of labeled lipids (i.e. labeled lipid divided by the sum of labeled and non-labeled lipid). The division is necessary to normalize for total lipid extracted, and ionization efficiency. As shown in Fig. S21B, there is a little difference in the amount of PS, PE and PC made by the CDP-DAG pathway specifically at later timepoints, correlating with the decreased growth of the cell at these time points (Fig S21A), and an overall slower metabolism. These differences however disappear when, as in figure 6, we plot the amount of PE made from PS or the amount of PC made from PE. These metrics are best suited to study exchange as they normalize for changes in the overall metabolic activity of the cell.
In conclusion, we think that with these explanations, the reviewer will agree that, by and large, Csf1 deletion does not abrogate exchange from the Psd to the Pmt enzyme.

-For these experiments the authors should provide the exact composition and amounts of "light" and "heavy" serine present in the medium

This oversight has been corrected in the material and methods.

-one important control for this experiment should also be the rate of autophagy appearing under the used growth conditions. If csf1Δ cells are starving under the used conditions, this could lead to mitophagy which would then feed heavy labeled PE into other pathways. A quick search in the HIP/HOP databases lists atg17Δ as the highest correlating deletion with csf1Δ.

We haven't found HIP/HOP data showing correlation with ATG17 (and have strong reservations about phenotypic analysis of *CSF1* deletions for reasons exposed in the response to reviewer 3, minor point 4). We nevertheless understand and share this concern. In fact, PE generated by the MIM-directed Psd could reach the pex-directed Pmt by any number of routes, direct or indirect, autophagy being just one of them.
However, the existence of various direct or indirect routes does not fundamentally change our conclusion that the loss of Csf1 does not affect the rates of PE to PC conversion, indicating that the rate of transport from the site of PE production to the site of PC production, whether direct or indirect (e.g. via autophagy) is not grossly affected. In support of this, Csf1 appears to be neither enriched at the mitochondria nor at the peroxisome.

We have extensively remodeled the text and the schematic in Fig 6C to phrase the question and its answer in a more inclusive way, considering the possibility of indirect means through which ^{15}N-PE can be converted to ^{15}N-PC.

Figure 7d and e:

-Why are only two replicates shown for some of the conditions?

There are two replicates for the wild type. Because there is only one isolate of wild type, this is a replicate of the same strain. In the case of the mutant, we generated three independent clones from this same wild type to ensure that any effect we observe was due to Csf1 impairment and not to a secondary unrelated passenger mutation in one odd clone. The changes in FA composition observed in the wild type strain are consistent with published literature, and therefore two replicates seem appropriate. We have now tested the statistical significance of the differences between wild type and *csf1Δ* strains (Fig 7D and E). All the meaningful comparisons are significant at 16C, while some are also significant at 30C (Fig. 7E).

Minor comments:

I also have a few small suggestions that I think would help to improve the impact of the manuscript. One small suggestions I have is that the labeling of the different conditions in the online searchable data becomes somewhat more easily comprehensible. And, if possible, I would suggest to make genes searchable in the datasets. Many researchers would be interested in a specific gene they are working on. Therefore this would be very beneficial for the study.

This is a great suggestion. We have extensively remodeled the web page to allow searching by gene (systematic or common name).

Page 11: The gene name DFN2 should probably read DNF2

Thanks for catching this mistake.

Reviewer 3

1.One assumption is that PC and PE are both essential. The production of PC and PE (as well as PS an PI) is tested in different strains in a largely qualitative way, although some differences in phospholipid levels are observed (but this is not so clear from the TLC plates).

Review COMMONS

The reviewer makes a valid point. However, the main reason to carry out the TLC experiments was to show that both PE and PC are being made in appreciable quantities. Differences in amount are bound to exist with foreign enzymes expressed from foreign promoters and localized to foreign compartments. Though it might be interesting to know the lipid composition in a more quantitative manner, we think that it will not change the conclusions on the genes necessary to thrive in the rewired conditions, which is the aim of our study.

-It would be useful to know if all rewired strains require both ethanolamine and choline for growth or if only one supplement might be sufficient in some cases.

Birner et al. (https://pubmed.ncbi.nlm.nih.gov/11294902/) have shown that choline alone can support growth of a mutant lacking both phosphatidylserine decarboxylases, and thus incapable of generating PE from the CDP-DAG pathway. Ethanolamine is not essential because small amounts of ethanolamine-phosphate can be generated through the breakdown of long-chain bases (LCB), which can then be incorporated into PE via the Kennedy pathway. Ethanolamine supply by LCB breakdown is however insufficient to support the biosynthesis of both PE and PC, hence the auxotrophy for either choline or ethanolamine. Ethanolamine is only truly required when LCB breakdown is also inhibited (for instance in dpl1 psd1 psd2 delete cells). The reason we add both ethanolamine and choline here is that they slightly enhance the growth of the choppΔ strain, when compared to supplementation with ethanolamine or choline only. This is now shown in new Fig 2A (gray traces).

Similar, are both pkPsd and aaPmt required in all cases or could the supplement of one enzyme be sufficient?

For example, work from de Kroon lab has shown that it is possible for yeast to survive without PC and they compensate by acyl chain shortening and saturation; see Bao et al., biorXiv 2021 https://doi.org/10.1101/2021.02.08.429707 and Boumann et al., Mol Biol Cell 2006. This work is also relevant for my comment #2 and for the discussion about the role of Csf1.

This is indeed an important point. In fact, even before the spectacular study of the de Kroon lab, it was known that double cho2 opi3 mutants could grow, even if very slowly, in the absence of choline (https://pubmed.ncbi.nlm.nih.gov/3066687/). We always thought that this residual growth might be the result of contaminating choline in the agar, but never tested this assumption. We therefore tested the growth of *choppΔ* strains expressing psd or pmt alone in liquid cultures without or with ethanolamine, choline, or both. We found that ethanolamine supplementation or the expression of any Psd enzyme alone enables some growth in the choppΔ strain (Fig 2A). Therefore, these strains might be able to grow somewhat similarly to what was observed by Ton de Kroon's study. However, the growth is very poor, and all strains do much better with choline. Incidentally, our observations about SAM5 tell us quite exactly how important choline synthesis is to these strains; when Pmt is targeted to the matrix, transposon mutants of sam5 depleting Pmt's cofactor SAM show a strong

Review COMMONS

dependency on the Kennedy pathway for growth, indicating that Pmt activity is required for meaningful growth in these conditions.

2. Another assumption repeated throughout the study is that rewired strains will cope with changes in the location of pkPsd and aaPmt by adapting their lipid transport pathways, specifically by adapting the levels of lipid transport proteins to bring PC and PE to their native location. In other words, that what is different between the strains is only where PC and PE are initially produced, not the membrane composition, which is checked by a single TLC analysis, therefore only for the main phospholipid species for whole cell lysates.
The analysis by transposon mutagenesis of the rewired strains does reveal that some lipid transfer proteins become required in some cases, in particular Vps13, but it doesn't seem that lipid transfer proteins are highly represented in the transposon screens (the requirement for flippases could be due to changes in membrane composition).

We can only agree with this statement and apologize if the reviewer understood that we assumed that rewired strains would cope only by adapting their lipid transport pathways, because it wasn't our view. Indeed, we anticipated that compensation by altering lipid composition would be a major way to cope with the challenge. This is an assertion that we repeat in many places throughout the manuscript including the abstract ("In rewired conditions, viability is expected to depend on **homeostatic adaptation to the ensuing lipostatic perturbations** and on efficient interorganelle lipid transport". Please note that we put adaptation before lipid transport), the introduction ("Importantly, cells are likely to adapt to the absence of one of these pathways by **activating homeostatic responses**. Indeed, mechanisms are known for sensing lipid levels and lipid packing/saturation (Young et al, 2010; Covino et al, 2016), which are coupled to transcriptional, post-transcriptional or allosteric responses."), the result section ("This suggests that cells must have transport mechanisms to re-distribute lipids from these non-native locations to other membranes around the cell, **as well as mechanisms to cope with lipid imbalance**."), the whole subsection of the result section entitled "**Uncovering adaptations in the rewired libraries**" (pp13-15), and the discussion section (the whole paragraph starting with "What molecular components allow yeast cells to survive rewiring and adapt to changes in organelle membrane composition?" and "Taken together, the extensive genetic interaction network reported here uncovers numerous lipid trafficking routes **and genes required to handle lipid imbalances generated by specific lipid rewiring conditions**.".
Moreover, we never mentioned that strains would cope "specifically by adapting the levels of lipid transport proteins", although this could be an interesting possibility, probably better tested by proteomics or transcriptomics.
That said, as our initial aim and broader scientific interest lies in lipid transport, we agree that we might have exaggerated expectations in this area. We therefore toned down the lipid trafficking aspect and toned up the lipid homeostasis aspect.

In particular, the hypothesis that the poorly characterized protein Csf1 is a novel lipid transfer protein is not confirmed. Therefore, the whole manuscript would benefit from being written in a

more open-ended manner with regards to how cells could cope with rewiring of their phospholipid metabolism, which could be different in different cases (as demonstrated for Csf1) and additional experiments to better characterize lipid composition/membrane properties of the rewired strains would be highly beneficial. Besides plasticity and redundancy of lipid transport pathways (at least in standard lab growth conditions), there are also compensatory effects between different lipids in membranes.

As stated above, we rephrased the manuscript in several points in a more open-ended manner and acknowledge that, additionally to plasticity and redundancy in lipid transport, there is also plasticity and diversity in adaptive mechanisms, and that lipid transport is not the whole story.

To the point of the reviewer that the study would benefit from a better characterization of lipid alterations in the rewired strains, we think that it would be difficult to meaningfully interpret a more quantitative knowledge of lipid composition in the context of the present study; we generated strains with exogenous enzymes expressed from non-regulated promoters and directed to ectopic membranes. Our study's scope is to identify genes (hence proteins) necessary for the adaptation to these unnatural conditions. Any change in lipid composition could be a direct effect of the enzymes we express, knock-on effects of these direct effects on other lipids, or the result of a compensatory mechanism. It could happen on a global scale, on defined intracellular membranes or even on parts of these membranes. Untangling these effects in a meaningful way would be far beyond the scope of the study.

-A more quantitative lipid analysis of rewired strains would be highly beneficial -- I realize that this is a lot of work, but this could add a lot of value to the subsequent genetic analyses. Less demanding phenotypic analyses of rewired strains could also be very informative, for example growth assays like those shown in Fig. 2A performed at different temperatures, which could be informative of changes in membrane composition (as shown later for csf1 mutants). Also, yeast often adapt to changes in phospholipid composition by producing more or less lipid droplets, so a quantification of lipid droplets by fluorescent microscopy or a quantification of neutral lipids could be informative of changes in membrane composition. These simple assays can be used to screen all rewired strains and then only strains displaying some phenotypic differences could be subjected to quantitative lipid analysis.

Please see response to previous point.

- Conclusions regarding lipid transport in the Discussion are too strong.

We softened the conclusions on lipid transport in many places in the discussion section.

3. A third assumption is that the steady-state localization of pkPsd and aaPmt constructs (based on the GFP signal) reflects the only sites of activity of the two enzymes. I realize that this assumption would be very difficult to test, but the authors should at least consider the possibility

that PC and PE could also be produced at other locations than at the desired destination, in particular at the ER.

We thought that we had carefully worded this concern first in the introduction section ("Importantly, in this strategy, data interpretation depends on the targeting accuracy of the chimeric enzymes to the intended organelles."), in the results section ("A minor fraction of LD- and peroxisome-targeted Pmt was detected in the ER in some cells (Fig S6 and S8)") and in the discussion section "This conclusion is contingent on the proper targeting of the lipid modifying enzymes."). As pointed by the reviewer, it is very difficult to test the absence of any ectopic activity. There was one condition however where the importance of ectopic activity could be assessed though; when the SAM-requiring Pmt was directed to the mitochondrial matrix, the mitochondrial SAM transporter Sam5 became necessary for growth in medium without ethanolamine and choline, indicating that the Pmt activity that allowed yeast growth was properly localized to mitochondria, and that any potential mislocalized activity was, in any case, insufficient. We now more directly address in the text the possibility of enzymes being partly mislocalized.

-What is the expected localization of pkPsd and aaPmt in different constructs with respect to cytosol/lumen? This should be stated. Is orientation of the active site preserved with respect to the native enzymes in different cases? (Diagrams in Fig. 1A imply that PE and PC would be produced in the lumen of the endosome or peroxisome?)

The topology of the yeast Pmt (Opi3 at least) has only recently been worked out with its active site facing the cytosol (https://pubmed.ncbi.nlm.nih.gov/31932304/). Yeast has two Psds. Psd1 is mostly found in the inner mitochondrial membrane with its active site facing the inter membrane space, with a small fraction on the ER, with the active site facing the cytosol. A very recent preprint also finds it at the lipid droplet, which is an exciting new development (https://www.biorxiv.org/content/10.1101/2021.08.31.458391v1). Psd2 is found on late-Golgi/early endosomes, with its active site facing the cytosol. Our constructs respect the topology of the native enzyme when this concept makes sense (e.g. when targeted to the inner mitochondrial membrane, the active site cannot face the cytosol). Figure 1A shows that we use Sec66 N-anchor to target the ER, with the enzyme in the cytosol. The same is true for LD targeting (Bsc2 N-anchor). For endosome targeting we use a FYVE domain that attaches to PI(3)P on the cytosolic surface of the endosome. Only for mitochondrial and peroxisome localization do we target the lumen. This has now been better explained in the text.

-Many of the constructs would be expected to traffic through the ER to reach their final destination and one could imagine that a fraction of their enzymatic activity occurs at the ER, even if the GFP signal is not observed at the ER, or that a small ER fraction is responsible for all PE/PC production. We do not know how active these enzymes are compared to yeast Psd and Cho2/Opi3, or how their level of expression compares to the expression of native yeast proteins. Furthermore, since ER is in contact with all other locations except for MIM and MM, some of the

pkPsd or aaPmt constructs could be acting in trans and still producing PE/PC on the ER membrane.

As a matter of fact, we explicitly chose targeting motifs that do not involve ER transit for the very reasons laid down by the reviewer, and none but perhaps one would be expected to traffic through the ER to reach their final destination. Mitochondrial localization does not involve ER transit, nor is peroxisomal targeting by the -SKL PTS motif. Endosomal targeting is achieved via a FYVE domain that binds PI(3)P made on the surface of endosomes. Only the LD targeting with Bsc2 TM domain might involve an insertion step in the ER before partitioning to the LDs. Indeed, we explicitly state that we observe some ER localization with this targeting motif in some cells. We now state that we specifically chose targeting motifs that avoided ER transit when possible.
That the enzymes act in trans is also a possibility that cannot be excluded, and might even be one way through which endogenous enzymes work. If *in trans* activity was required for growth, then it would be expected that non-essential tethers might become essential, an additional information to mine for in our data. We now explicitly acknowledge this possibility.

-In the labels in Fig 2A, it would be more fair to say 'Psd localization' and 'Pmt localization' instead of 'PE source' and 'PC source', and the authors should address possible caveats.

Indeed. We had originally chosen PE source and PC source out of clarity (as Pmt and Psd might not mean much to many readers), but we agree that this oversimplification is misleading. We have therefore changed to ‘Psd targeting’ and ‘Pmt targeting’. We chose ‘targeting’ over ‘localization’, since, as pointed by this reviewer and as we have observed in some cases, targeting to one organelle does not imply that 100% of the enzyme is localized to that organelle.

4.The Csf1 finding and its possible role in acyl chain remodeling is intriguing, although inconclusive and will require future studies.

We agree that the finding on Csf1 does not inform on the mechanistic details of this protein’s biochemistry. Nevertheless, as stated by reviewer #1, “the characterization of Csf1, albeit preliminary, paves the way to future studies” and provide proof of principle of the usefulness of our data. We have toned down the conclusions on Csf1’s role as a lipid exchanger. More importantly, our new data provide a causal link between the lipid remodeling defect and csf1 mutant phenotype, as oleic acid supplementation suppresses to a large extent their cold-sensitivity.

Minor comments:

-The authors became interested in Csf1 due to the chorein-N domain homology; however it should be noted that the function of this domain is not known and it is not sufficient for lipid transport in Vps13.

We respectfully disagree with this statement. A fragment of Vps13 containing the chorein-N domain has been crystalized, shown *in vitro* to transport lipids between liposomes, and found to be part of a larger lipid-binding tunnel (Kumar et al. JCB, 2018, Li et al, JCB, 2020). The same has been found for the chorein-N domain of Atg2 (Valverde et al. 2019). While the reviewer is correct that the domain "is not sufficient for lipid transport", it is clear from these high-resolution structures as well as lower-resolution CryoEM structures that the Chorein-N domain is part of a larger lipid-binding groove and folds as such. Below is a superposition of S. pombe ATG2 structure (6A9J, blue) with liganded PE, and C. thermophilum Vps13 (green, 6CBC). The Chorein-N domains are highlighted in orange and red, respectively, and they clearly are part of the lipid-binding grooves, which extend over large expanse of the protein. See also our response to reviewer 1, point C4.

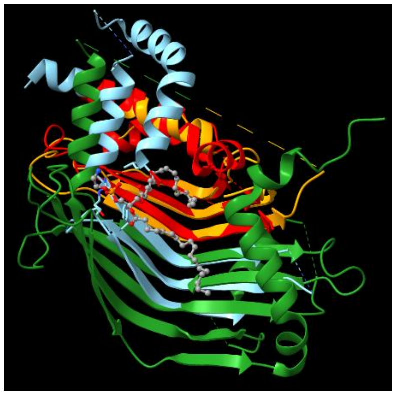

The recent alpha-fold release shows that Chorein-N domain containing proteins from taxa as distant as plants (A0A0R0HDJ1_SOYBN), trypanozomatids (Q4DK87_TRYCC) or metazoans (G5EE16_CAEEL) all fold into similar hydrophobic grooves, the N-terminus of which is constituted by the Chorein-N domain. There is sufficient evidence to say that the Chorein-N domain is part of a lipid-binding groove, and it is therefore logical to hypothesize that a Chorein-N-domain-containing protein such as Csf1 might be a lipid transporter like Vps13 or Atg2.

-It would be informative to also mention the large size of Csf1; is it predicted to be soluble? Localization?

We now mention the large size of Csf1, comparable to that of Vps13. The localization of Csf1 is depicted in figure 7A as peripheral foci that colocalize with the ER. The presence of a TM domain is clearly shown in figure 6A, right panel, therefore, the protein is clearly not predicted to be soluble. The localization we observe is consistent with ER-PM contact sites. In particular the rather short TM domain of Csf1 (21 aa) is unlikely to be inserted in the PM (see Sharpe, Stevens, Munro. 2010), and we never observe Csf1 foci on the nuclear envelope. We cannot however claim here that Csf1 is at ER-PM contacts as it would require identifying and disrupting the interaction(s) that might keep Csf1 at PM contact sites, something beyond the scope of this paper. We have nevertheless commented more thoroughly on Csf1 localization in the text.

-Top of page 19: the authors present no evidence that Csf1 function is affecting lipid distribution, so this sentence should be corrected.

Agreed. We really meant "homeostasis", and not "distribution".

-With respect to what was previously known about Csf1, two studies uncovered csf1Δ in screens for genes involved in the maintenance of ER homeostasis/protein folding and secretion (see Copic et al., 2009, Genetics 182, 757 & Jonikas et al., 2009, Science 323, 1693), including a synthetic interaction with hac1Δ. Given the established connections between ER membrane composition/stress and ER homeostasis, these phenotypes seem highly relevant, and the statement on page 19 that the only well-documented phenotype of csf1 mutants is cold sensitivity should be amended.

We have mentioned a problem in the inconsistent phenotypes of Csf1 full deletions, some of which appeared to grow slowly even at permissive temperature. We have since then dug into the heart of the problem and found out that *CSF1* deletion interferes with the neighboring GAA1 gene, an essential component of the GPI anchoring machinery. This means that all large-scale genetic analyses using deletion libraries cannot be trusted when it comes to *CSF1*. This is strikingly shown in the Cell Map (Costanzo et al.) where *CSF1* is found amidst a cluster of glycosylation and GPI-anchor biosynthesis genes (in green in figure below), showing that the GPI-related phenotype of *GAA1* interference, and not of Csf1 deficiency per se, dominates these analyses.

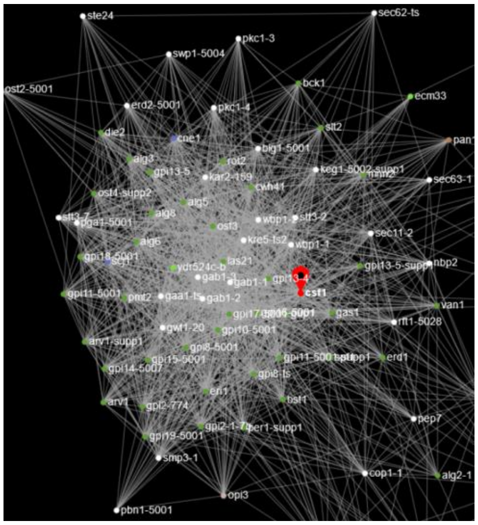

The Copic et al. study referred to by the reviewer finds Csf1 among a series of genes with roles "in protein glycosylation, GPI-anchor attachment, ER quality control, and retrieval of escaped ER residents". The synthetic lethality with components of the UPR pathway likely also boils down to a problem with *GAA1* expression; in the Costanzo dataset, *hac1* is the 21st mutant with strongest negative interaction with a thermosensitive allele of *gaa1*; *ire1* is 8th (preceded only by other hypomorphic alleles of the GPI-anchor machinery).

In addition, we have found that CSF1 full deletion cells grow slowly but rapidly acquire suppressor mutations. These mutations likely involve the inactivation of the *HOG1* pathway, since a SATAY analysis in two *CSF1*

deletion shows a strong selection for mutants in *HOG1*, *PBS2*, *SSK1*, as well as in transcriptional regulators acting downstream of this MAP kinase cascade.
Therefore, all phenotypes, genetic interaction and drug sensitivities reported for full deletions of *CSF1* cannot be trusted. The cold-sensitive phenotype, by contrast, has been identified in a spontaneous mutant, thence unlikely to affect *GAA1* expression, and our insertion mutant confirms this. We will therefore remain with our assertion that cold sensitivity is the only well-documented phenotype of *csf1* mutants, but now provide an explanation for this statement.

-Intro, first paragraph: 10-30 nm, not nM

Many thanks for catching this typo.

-Intro, last paragraph: correct 'minimalizing'

Many thanks again. Corrected.

-scPsd instead of pkPsd in MIM panel of Fig. 1B ?

This is not a typo, but as stated in the text we have used a previously published (duly referenced) construct where scPSD is targeted exclusively to the MIM, while for other constructs we have utilized PSD from P. knowlesi lacking the TM domain for redirecting: ("Specifically, we utilized a PS decarboxylase (Psd) lacking the transmembrane domain, either from yeast (scPSD) or Plasmodium knowlesi (pkPsd) and ..") and ("We expressed the chimeric enzymes on a plasmid under the control of the TEF promoter in the *choppΔ* strain, except for yeast *PSD1*, which was expressed from its own promoter (Friedman et al, 2018).")
For clarity, we now refer to the scPSD explicitly in the text.

Dear Dr Kornmann,

Thank you for submitting your revised manuscript (EMBOJ-2021-109998) to The EMBO Journal, as well as your patience with our response. Your amended study was sent back to the three reviewers for re-evaluation, and we have received comments from all of them, which I enclose below.

The referees stated that their issues have been comprehensively resolved and they are now broadly in favour of publication, pending minor revision.

Thus, we are pleased to inform you that your manuscript has been accepted in principle for publication in The EMBO Journal.

Please address the remaining minor issues stated by referees #2 and #3 carefully by adjusting the nomenclature and data display where appropriate. Further, we need you to consider a number of points related to formatting and data deposition as detailed below, which should be addressed at re-submission.

Please contact me at any time if you have additional questions related to below points.

As you might have noted on our web page, every paper at the EMBO Journal now includes a 'Synopsis', displayed on the html and freely accessible to all readers. The synopsis includes a 'model' figure as well as 2-5 one-short-sentence bullet points that summarize the article. I would appreciate if you could provide this figure and the bullet points.

Thank you for giving us the chance to consider your manuscript for The EMBO Journal. I look forward to your final revision.

Again, please contact me at any time if you need any help or have further questions.

Kind regards,

Daniel Klimmeck

Daniel Klimmeck PhD
Senior Editor
The EMBO Journal

Formatting changes required for the final revised version of the manuscript:

>> Please add up to five keywords to the manuscript.

>> Add a separate 'Statistical analysis' section to your manuscript, detailing the algorithms applied.

>> Please update your funding information in our online portal. Currently, Synapsis Foundation and ETH Zürich, are missing.

>> Define B. K. as corresponding author on the manuscript title page.

>> Add a header to the 'Conflict of Interest' section.

>> Provide main figures and EV figures as individual, high-resolution .tiff files. Add their legends to the manuscript, after the main figure legends.

>> Please introduce a data accessibility section detailing the ENA data sets and related http hyperlinks. Ensure privacy is released.

>> Compile supplemental figures and legends as one 'Appendix' file with a ToC on its first page, save as PDF, rename figures to "Appendix Figure S1" etc.; Add three Suppl. Tables from the other doc, rename to "Appendix Table S1" etc. Current Suppl Tables 1-3 and Material/Fig1-22 should be included in the Appendix.

>> Current Suppl Data zip files and three Excel sheets should be made source data.

>> Recheck the bioRxiv references for updates.

>> Please consider additional changes and comments from our production team as indicated by the .doc file enclosed and leave changes in track mode.

****

Instructions for preparing your revised manuscript:

Please check that the title and abstract of the manuscript are brief, yet explicit, even to non-specialists.

When assembling figures, please refer to our figure preparation guideline in order to ensure proper formatting and readability in print as well as on screen:
https://bit.ly/EMBOPressFigurePreparationGuideline

IMPORTANT: When you send the revision we will require
- a point-by-point response to the referees' comments, with a detailed description of the changes made (as a word file).
- a word file of the manuscript text.
- individual production quality figure files (one file per figure)
- a complete author checklist, which you can download from our author guidelines (https://www.embopress.org/page/journal/14602075/authorguide).
- Expanded View files (replacing Supplementary Information)
Please see out instructions to authors
https://www.embopress.org/page/journal/14602075/authorguide#expandedview

Please remember: Digital image enhancement is acceptable practice, as long as it accurately represents the original data and conforms to community standards. If a figure has been subjected to significant electronic manipulation, this must be noted in the figure legend or in the 'Materials and Methods' section. The editors reserve the right to request original versions of figures and the original images that were used to assemble the figure.

Further information is available in our Guide For Authors: https://www.embopress.org/page/journal/14602075/authorguide

Revision to The EMBO Journal should be submitted online within 90 days, unless an extension has been requested and approved by the editor; please click on the link below to submit the revision online before 15th Feb 2022:

Link Not Available

Referee #1:

As already pointed out in my previous report I think that the manuscript is highly valuable for the community. During the revision process the authors have satisfied all of the critical concerns and the manuscript should be published without delay.

Referee #2:

The authors have addressed my points in a mostly satisfactory manner. Some remaining concerns:

1. The new figure 2A is very informative and it adds very important context to the tests of growth on plates in figure 2B. Frankly, in light of fig 2A, I find 2B a bit misleading; yes, all combinations in 2B grow under Kennedy-off conditions, but so do all strains expressing only Psd enzymes in Kennedy-off conditions (although considerably less well than in Kennedy-on). It is also striking to what extent the growth of Pmt-expressing strains is improved by the addition of choline. The situation is clearly not as black and white as what is suggested by panel 2B.

A lot of work went into this study, so I think that it would be too much at this point to ask that the data in fig. 2B be presented in the form of growth curves so that we could see the relative improvement between the expression of single or double enzymes.

But Fig 2A needs to be properly presented. Please add error bars to all curves to show reproducibility between independent experiments and make the graphs bigger and the colors of different curves more distinct so it is clear which curve represents which enzyme (I cannot tell which Pmt curve is which). Also, Psd-MIM is missing from pannel 2A.

The text added along with Fig 2A is also important; I think it would be useful for the readers to add a reference to these caveats to the Discussion and/or the Intro.

There is a typo in the label for Pmt-MIM in Fig 2A.

2. In the Methods section, please specify the exact composition of media that were used for the growth assays, including the suppliers. This study could be cited: Renne MF, Bao X, de Kroon AIPM. Water soluble lipid precursor contaminants in yeast culture medium ingredients. FEMS Yeast Res. 2020 Aug 1;20(5):foaa029. doi: 10.1093/femsyr/foaa029. PMID: 32592392; PMCID: PMC8260331.

Referee #3:

This manuscript is a revised version from an initial submission that was already of excellent quality. As I said previously, this is an impressive work and a great resource for other researchers in the field. This study paves the way for multitude of studies and discoveries in the field of lipid fluxes, homeostasis and membrane biogenesis in eukaryotes.
All conclusions are well substantiated and discussed.
This is really very well written and clear. The corrections and additions highlighted in red address all previous concerns. The Author responses to Reviewers are clear and well supported.

I recommend publication.

A very minor comment.
Because I have to complain about something I would suggest that the authors stick to Chorein-N 'motif' rather than 'domain' for example in the abstract
'Csf1 -an uncharacterized protein harboring a Chorein-N lipid transport domain- '
maybe say Csf1 -an uncharacterized protein harboring a Chorein-N motif associated with (or found in) bulk lipid transport proteins...

This is not dramatic but the motif (rather short ~100 residues) is only a portion of the domain (as revealed in two crystal structures) while the cryo-EM structures of larger fragments of Vps13 or full length Atg2A also suggest it is part of a much larger structural 'block' based on curved antiparallel -sheets. This would apply also page 4 and page 15 and page 22. As I said not a big deal.

Thank you for graphically clarifying and illustrating the Vps13 case in Figure 3C.

One typo.
Page 15. Small typo for the comma after '10th,' They are respectively the 10th, 5th and 92nd..."

***
Rev_Com_number: RC-2021-00962
New_manu_number: EMBOJ-2021-109998
Corr_author: Kornmann
Title: Rewiring Phospholipid Biosynthesis Reveals Resilience to Membrane Perturbations and Uncovers Regulators of Lipid Homeostasis

1. The new figure 2A is very informative and it adds very important context to the tests of growth on plates in figure 2B. Frankly, in light of fig 2A, I find 2B a bit misleading; yes, all combinations in 2B grow under Kennedy-off conditions, but so do all strains expressing only Psd enzymes in Kennedy-off conditions (although considerably less well than in Kennedy-on). It is also striking to what extent the growth of Pmt-expressing strains is improved by the addition of choline. The situation is clearly not as black and white as what is suggested by panel 2B.

A lot of work went into this study, so I think that it would be too much at this point to ask that the data in fig. 2B be presented in the form of growth curves so that we could see the relative improvement between the expression of single or double enzymes. But Fig 2A needs to be properly presented. Please add error bars to all curves to show reproducibility between independent experiments and make the graphs bigger and the colors of different curves more distinct so it is clear which curve represents which enzyme (I cannot tell which Pmt curve is which). Also, Psd-MIM is missing from pannel 2A.

We have replicated our growth assays for Fig 2A in order to present error bars. We have also increased the graph's size and changed the color scheme to make the data clearer

The text added along with Fig 2A is also important; I think it would be useful for the readers to add a reference to these caveats to the Discussion and/or the Intro.

We added/modified two sentences in the introduction to mention the possibility of growth without PC: "Either pathway alone is necessary and sufficient for optimal growth, indicating that lipids are distributed throughout the cell from different locations." And "A spectacular example is the adaptation to the lack of PC synthesis which involves remodeling of fatty acids in phospholipids."

There is a typo in the label for Pmt-MIM in Fig 2A.

Thanks, fixed.

2. In the Methods section, please specify the exact composition of media that were used for the growth assays, including the suppliers. This study could be cited: Renne MF, Bao X, de Kroon AIPM. Water soluble lipid precursor contaminants in yeast culture medium ingredients. FEMS Yeast Res. 2020 Aug 1;20(5):foaa029. doi: 10.1093/femsyr/foaa029. PMID: 32592392; PMCID: PMC8260331.

We added an Appendix method with media recipes. Thanks for pointing to this paper, which we hadn't seen. This is indeed a valid possibility for residual growth in cho2 opi3 mutants, now duly acknowledged.

Referee #3:

Because I have to complain about something I would suggest that the authors stick to Chorein-N 'motif' rather than 'domain'
for example in the abstract
'Csf1 -an uncharacterized protein harboring a Chorein-N lipid transport domain- '
maybe say Csf1 -an uncharacterized protein harboring a Chorein-N motif associated with (or found in) bulk lipid transport proteins...

This is not dramatic but the motif (rather short ~100 residues) is only a portion of the domain (as revealed in two crystal structures) while the cryo-EM structures of larger fragments of Vps13 or full length Atg2A also suggest it is part of a much larger structural 'block' based on curved antiparallel β-sheets. This would apply also page 4 and page 15 and page 22. As I said not a big deal.

Although the nomenclature in the field is unclear about domain/motif, we agree with the reviewer and have changed all occurrences of Chorein-N domain.

Thank you for graphically clarifying and illustrating the Vps13 case in Figure 3C.
You're very welcome, thanks for the suggestion.

One typo.
Page 15. Small typo for the comma after '10th,' They are respectively the 10th, 5th and 92nd..."

Corrected, thanks!

Dear Benoit, dear Sabine and Arun,

Thank you for submitting the revised version of your manuscript. I have now evaluated your amended manuscript and concluded that the remaining minor concerns have been sufficiently addressed.

Thus, I am pleased to inform you that your manuscript has been accepted for publication in the EMBO Journal.

Please note that it is EMBO Journal policy for the transcript of the editorial process (containing referee reports and your response letter) to be published as an online supplement to each paper. I would thus like to ask for your consent on keeping the additional referee figures included in this file.

Also, in case you might NOT want the transparent process file published at all, you will also need to inform us via email immediately. More information is available here: http://emboj.embopress.org/about#Transparent_Process

----

Please note that in order to be able to start the production process, our publisher will need and contact you regarding the following forms:

- PAGE CHARGE AUTHORISATION (For Articles and Resources)
http://onlinelibrary.wiley.com/journal/10.1002/(ISSN)1460-2075/homepage/tej_apc.pdf

- LICENCE TO PUBLISH (for non-Open Access)

Your article cannot be published until the publisher has received the appropriate signed license agreement. Once your article has been received by Wiley for production you will receive an email from Wiley's Author Services system, which will ask you to log in and will present them with the appropriate license for completion.

- LICENCE TO PUBLISH for OPEN ACCESS papers

Authors of accepted peer-reviewed original research articles may choose to pay a fee in order for their published article to be made freely accessible to all online immediately upon publication. The EMBO Open fee is fixed at $5,200 (+ VAT where applicable).

We offer two licenses for Open Access papers, CC-BY and CC-BY-NC-ND.
For more information on these licenses, please visit: http://creativecommons.org/licenses/by/3.0/ and
http://creativecommons.org/licenses/by-nc-nd/3.0/deed.en_US

- PAYMENT FOR OPEN ACCESS papers

You also need to complete our payment system for Open Access articles. Please follow this link and select EMBO Journal from the drop-down list and then complete the payment process: https://authorservices.wiley.com/bauthor/onlineopen_order.asp

Should you be planning a Press Release on your article, please get in contact with embojournal@wiley.com as early as possible, in order to coordinate publication and release dates.

On a different note, I would like to alert you that EMBO Press is currently developing a new format for a video-synopsis of work published with us, which essentially is a short, author-generated film explaining the core findings in hand drawings, and, as we believe, can be very useful to increase visibility of the work. This has proven to offer a nice opportunity for exposure i.p. for the first author(s) of the study. Please see the following link for representative examples and their integration into the article web page:
https://www.embopress.org/video_synopses
https://www.embopress.org/doi/full/10.15252/embj.2019103932

Please let me know, should you be interested to engage in commissioning a similar video synopsis for your work. According operation instructions are available and intuitive.

Finally, we have noted that the submitted version of your article is also posted on the preprint platform bioRxiv. We would

appreciate if you could alert bioRxiv on the acceptance of this manuscript at The EMBO Journal in order to allow for an update of the entry status. Thank you in advance!

If you have any questions, please do not hesitate to call or email the Editorial Office.

Thank you again for this contribution to The EMBO Journal and congratulations on a successful publication! Please consider us again in the future for your most exciting work.

with
Best regards,

Daniel

Daniel Klimmeck, PhD
Senior Editor
The EMBO Journal
EMBO
Postfach 1022-40
Meyerhofstrasse 1
D-69117 Heidelberg
contact@embojournal.org
Submit at: http://emboj.msubmit.net

***
Rev_Com_number: RC-2021-00962
New_manu_number: EMBOJ-2021-109998R
Corr_author: Kornmann
Title: Rewiring Phospholipid Biosynthesis Reveals Resilience to Membrane Perturbations

EMBO PRESS

YOU MUST COMPLETE ALL CELLS WITH A PINK BACKGROUND ➔

PLEASE NOTE THAT THIS CHECKLIST WILL BE PUBLISHED ALONGSIDE YOUR PAPER

Corresponding Author Name: Benoit Kornmann, Arun T John Peter
Journal Submitted to: EMBO Journal
Manuscript Number: EMBOJ-2021-109998

Reporting Checklist For Life Sciences Articles (Rev. June 2017)

This checklist is used to ensure good reporting standards and to improve the reproducibility of published results. These guidelines are consistent with the Principles and Guidelines for Reporting Preclinical Research issued by the NIH in 2014. Please follow the journal's authorship guidelines in preparing your manuscript.

A- Figures
1. Data
The data shown in figures should satisfy the following conditions:

- ➔ the data were obtained and processed according to the field's best practice and are presented to reflect the results of the experiments in an accurate and unbiased manner.
- ➔ figure panels include only data points, measurements or observations that can be compared to each other in a scientifically meaningful way.
- ➔ graphs include clearly labeled error bars for independent experiments and sample sizes. Unless justified, error bars should not be shown for technical replicates.
- ➔ if n< 5, the individual data points from each experiment should be plotted and any statistical test employed should be justified
- ➔ Source Data should be included to report the data underlying graphs. Please follow the guidelines set out in the author ship guidelines on Data Presentation.

2. Captions

Each figure caption should contain the following information, for each panel where they are relevant:

- ➔ a specification of the experimental system investigated (eg cell line, species name).
- ➔ the assay(s) and method(s) used to carry out the reported observations and measurements
- ➔ an explicit mention of the biological and chemical entity(ies) that are being measured.
- ➔ an explicit mention of the biological and chemical entity(ies) that are altered/varied/perturbed in a controlled manner.

- ➔ the exact sample size (n) for each experimental group/condition, given as a number, not a range;
- ➔ a description of the sample collection allowing the reader to understand whether the samples represent technical or biological replicates (including how many animals, litters, cultures, etc.).
- ➔ a statement of how many times the experiment shown was independently replicated in the laboratory.
- ➔ definitions of statistical methods and measures:
  - common tests, such as t-test (please specify whether paired vs. unpaired), simple $\chi 2$ tests, Wilcoxon and Mann-Whitney tests, can be unambiguously identified by name only, but more complex techniques should be described in the methods section;
  - are tests one-sided or two-sided?
  - are there adjustments for multiple comparisons?
  - exact statistical test results, e.g., P values = x but not P values < x;
  - definition of 'center values' as median or average;
  - definition of error bars as s.d. or s.e.m.

Any descriptions too long for the figure legend should be included in the methods section and/or with the source data.

In the pink boxes below, please ensure that the answers to the following questions are reported in the manuscript itself. Every question should be answered. If the question is not relevant to your research, please write NA (non applicable). We encourage you to include a specific subsection in the methods section for statistics, reagents, animal models and human subjects.

B- Statistics and general methods

Please fill out these boxes ➔ (Do not worry if you cannot see all your text once you press return)

1.a. How was the sample size chosen to ensure adequate power to detect a pre-specified effect size?	Sample size for figure 7D and E was chosen according to the known effect size of temperature on fatty acid composition (Klose C, Surma MA, Gerl MJ, Meyenhofer F, Shevchenko A, Simons K. Flexibility of a eukaryotic lipidome--insights from yeast lipidomics. PLoS One. 2012;7(4):e35063. doi: 10.1371/journal.pone.0035063. Epub 2012 Apr 18. PMID: 22529973; PMCID: PMC3329542.)
1.b. For animal studies, include a statement about sample size estimate even if no statistical methods were used.	Not applicable
2. Describe inclusion/exclusion criteria if samples or animals were excluded from the analysis. Were the criteria pre-established?	Not applicable
3. Were any steps taken to minimize the effects of subjective bias when allocating animals/samples to treatment (e.g. randomization procedure)? If yes, please describe.	Not applicable
For animal studies, include a statement about randomization even if no randomization was used.	Not applicable
4.a. Were any steps taken to minimize the effects of subjective bias during group allocation or/and when assessing results (e.g. blinding of the investigator)? If yes please describe.	Not applicable
4.b. For animal studies, include a statement about blinding even if no blinding was done	Not applicable
5. For every figure, are statistical tests justified as appropriate?	Yes
Do the data meet the assumptions of the tests (e.g., normal distribution)? Describe any methods used to assess it.	Not applicable
Is there an estimate of variation within each group of data?	Not applicable

USEFUL LINKS FOR COMPLETING THIS FORM

http://www.antibodypedia.com
http://1degreebio.org
http://www.equator-network.org/reporting-guidelines/improving-bioscience-research-repor
http://grants.nih.gov/grants/olaw/olaw.htm
http://www.mrc.ac.uk/Ourresearch/Ethicsresearchguidance/Useofanimals/index.htm
http://ClinicalTrials.gov
http://www.consort-statement.org
http://www.consort-statement.org/checklists/view/32-consort/66-title
http://www.equator-network.org/reporting-guidelines/reporting-recommendations-for-tumo
http://datadryad.org
http://figshare.com
http://www.ncbi.nlm.nih.gov/gap
http://www.ebi.ac.uk/ega
http://biomodels.net/
http://biomodels.net/miriam/
http://jjj.biochem.sun.ac.za
http://oba.od.nih.gov/biosecurity/biosecurity_documents.html
http://www.selectagents.gov/

Is the variance similar between the groups that are being statistically compared?	Not applicable

C- Reagents

6. To show that antibodies were profiled for use in the system under study (assay and species), provide a citation, catalog number and/or clone number, supplementary information or reference to an antibody validation profile. e.g., Antibodypedia (see link list at top right), 1DegreeBio (see link list at top right).	Not applicable
7. Identify the source of cell lines and report if they were recently authenticated (e.g., by STR profiling) and tested for mycoplasma contamination.	Not applicable

* for all hyperlinks, please see the table at the top right of the document

D- Animal Models

8. Report species, strain, gender, age of animals and genetic modification status where applicable. Please detail housing and husbandry conditions and the source of animals.	Not applicable
9. For experiments involving live vertebrates, include a statement of compliance with ethical regulations and identify the committee(s) approving the experiments.	Not applicable
10. We recommend consulting the ARRIVE guidelines (see link list at top right) (PLoS Biol. 8(6), e1000412, 2010) to ensure that other relevant aspects of animal studies are adequately reported. See author guidelines, under 'Reporting Guidelines'. See also: NIH (see link list at top right) and MRC (see link list at top right) recommendations. Please confirm compliance.	Not applicable

E- Human Subjects

11. Identify the committee(s) approving the study protocol.	Not applicable
12. Include a statement confirming that informed consent was obtained from all subjects and that the experiments conformed to the principles set out in the WMA Declaration of Helsinki and the Department of Health and Human Services Belmont Report.	Not applicable
13. For publication of patient photos, include a statement confirming that consent to publish was obtained.	Not applicable
14. Report any restrictions on the availability (and/or on the use) of human data or samples.	Not applicable
15. Report the clinical trial registration number (at ClinicalTrials.gov or equivalent), where applicable.	Not applicable
16. For phase II and III randomized controlled trials, please refer to the CONSORT flow diagram (see link list at top right) and submit the CONSORT checklist (see link list at top right) with your submission. See author guidelines, under 'Reporting Guidelines'. Please confirm you have submitted this list.	Not applicable
17. For tumor marker prognostic studies, we recommend that you follow the REMARK reporting guidelines (see link list at top right). See author guidelines, under 'Reporting Guidelines'. Please confirm you have followed these guidelines.	Not applicable

F- Data Accessibility

18: Provide a "Data Availability" section at the end of the Materials & Methods, listing the accession codes for data generated in this study and deposited in a public database (e.g. RNA-Seq data: Gene Expression Omnibus GSE39462, Proteomics data: PRIDE PXD000208 etc.) Please refer to our author guidelines for 'Data Deposition'.<br><br>Data deposition in a public repository is mandatory for:<br>a. Protein, DNA and RNA sequences<br>b. Macromolecular structures<br>c. Crystallographic data for small molecules<br>d. Functional genomics data<br>e. Proteomics and molecular interactions	Data Availability<br>Sequencing data are deposited in the European Nucleotide Archive (ENA) under accession number PRJEB48777. Data analyses are available in the supplementary data, at http://genome-euro.ucsc.edu/s/benjou/rewiring_paper and https://kornmann.bioch.ox.ac.uk/satay/rewiring.
19. Deposition is strongly recommended for any datasets that are central and integral to the study; please consider the journal's data policy. If no structured public repository exists for a given data type, we encourage the provision of datasets in the manuscript as a Supplementary Document (see author guidelines under 'Expanded View' or in unstructured repositories such as Dryad (see link list at top right) or Figshare (see link list at top right).	Sequencing data are deposited in the European Nucleotide Archive (ENA) under accession number PRJEB48777
20. Access to human clinical and genomic datasets should be provided with as few restrictions as possible while respecting ethical obligations to the patients and relevant medical and legal issues. If practically possible and compatible with the individual consent agreement used in the study, such data should be deposited in one of the major public access-controlled repositories such as dbGAP (see link list at top right) or EGA (see link list at top right).	Not applicable
21. Computational models that are central and integral to a study should be shared without restrictions and provided in a machine-readable form. The relevant accession numbers or links should be provided. When possible, standardized format (SBML, CellML) should be used instead of scripts (e.g. MATLAB). Authors are strongly encouraged to follow the MIRIAM guidelines (see link list at top right) and deposit their model in a public database such as Biomodels (see link list at top right) or JWS Online (see link list at top right). If computer source code is provided with the paper, it should be deposited in a public repository or included in supplementary information.	Not applicable

G- Dual use research of concern

22. Could your study fall under dual use research restrictions? Please check biosecurity documents (see link list at top right) and list of select agents and toxins (APHIS/CDC) (see link list at top right). According to our biosecurity guidelines, provide a statement only if it could.	No
